# The Metaphosphite (PO_2_^−^) Anion as a Ligand

**DOI:** 10.1002/anie.202011750

**Published:** 2020-10-25

**Authors:** Josh Abbenseth, Florian Wätjen, Markus Finger, Sven Schneider

**Affiliations:** ^1^ Universität Göttingen Institut für Anorganische Chemie Tammannstrasse 4 37077 Göttingen Germany

**Keywords:** metaphosphite, oxygen atom transfer, phosphorous, pincer complex, rhenium

## Abstract

The utilization of monomeric, lower phosphorous oxides and oxoanions, such as metaphosphite (PO_2_^−^), which is the heavier homologue of the common nitrite anion but previously only observed in the gas phase and by matrix isolation, requires new synthetic strategies. Herein, a series of rhenium(I–III) complexes with PO_2_^−^ as ligand is reported. Synthetic access was enabled by selective oxygenation of a terminal phosphide complex. Spectroscopic and computational examination revealed slightly stronger σ‐donor and comparable π‐acceptor properties of PO_2_^−^ compared to homologous NO_2_^−^, which is one of the archetypal ligands in coordination chemistry.

## Graphical abstract

Oxygenation of a terminal phosphide complex allowed the synthesis of rhenium(I–III) complexes with the unknown metaphosphite (PO_2_^−^) ligand. Spectroscopic, crystallographic, and computational examinations revealed slightly stronger σ‐donating and comparable π‐accepting properties as compared to the archetypal nitrite ligand.

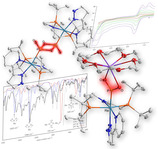

The nitrogen oxides NO, NO_2_ and N_2_O are of high environmental importance as key species in atmospheric nitrogen chemistry.^[1]^ Their extensive use as neutral or charged ligands dates back to the founding days of coordination chemistry and is currently stimulated by biological transformations of the global nitrogen cycle, where the nitrite anion (NO_2_^−^) stands out as a connecting intermediate.[^2^, ^3^] In contrast, PO, PO_2_ and P_2_O are highly reactive species, e.g., as intermediates in the combustion of phosphorous‐based flame retardants.^[4]^ For example, the electron affinity of PO_2_ (3.4 eV) is close to that of atomic fluorine and strongly exceeds that of NO_2_ (2.3 eV).^[5]^ The resulting metaphosphite anion (PO_2_^−^),^[6]^ which is isoelectronic with SO_2_, readily oligomerizes to cyclohexametaphosphite (P_6_O_12_^6−^).^[7]^ Examinations of monomeric lower phosphorous oxides and oxoanions generally require matrix isolation or gas phase techniques.^[8]^ Strategies for controlled access and stabilization are therefore desirable to exploit them as synthetic building blocks.

Lewis‐base stabilization was utilized for the isolation of P_2_O_4_ (Figure 1 a) and cationic PO_*n*_^+^ (*n*=1, 2) species.[^9^, ^10^] In addition, some transition metal clusters with bridging P_*x*_O_*y*_ building blocks were reported (e.g. Figure 1 b), e.g., via oxygen atom transfer (OAT) reactions to (di‐)phosphide ligands.[^8^, ^11^] Cummins’ PO‐complex (Figure 1 d) stands out as a unique mononuclear example.^[12]^ The only known PO_2_‐containing cluster (Figure 1 c) features trianionic, side‐on bound hypophosphite (PO_2_^3−^).^[11b]^ In contrast, authentic monometaphosphite (PO_2_^−^) ligands, as phosphorous analogues of the common nitrite anion, remain elusive.

**Figure 1:**
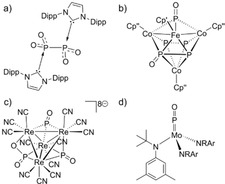
Selected examples of isolable, low‐molecular phosphorus oxide compounds (Cp′=C_5_Me_5_, Cp′′=1,3‐^*t*^Bu_2_C_5_H_3_, Dipp=2,6‐^*i*^Pr_2_C_6_H_3_).

The introduction of 2‐phosphaethynolate as P‐atom transfer reagent recently gave access to more electron rich (d^2^) terminal phosphide complexes, such as [ReP(κ‐*N* ^2^‐PyrPz)(PNP)] (**1**, ^H^PyrPz=2‐(1*H*‐pyrazol‐5‐yl)pyridine, PNP=N(CH_2_CH_2_P*t*Bu_2_)_2_; Scheme 1).[^13^, ^14^] In continuation, we here report OAT reactivity as synthetic entry to lower phosphorous oxide ligands. Facile oxygenation of **1** enabled the synthesis of rhenium(I–III) metaphosphite complexes and examination of M‐PO_2_ bonding.

**Scheme 1:**
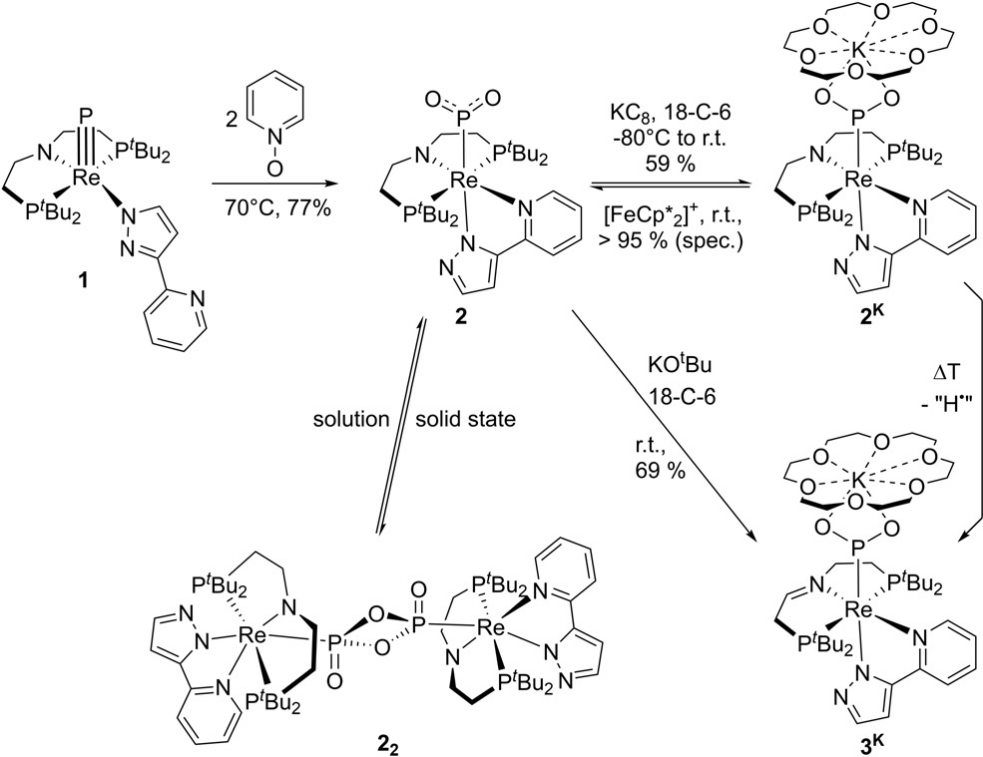
Synthesis of metaphosphite complexes **2**, **2^K^**, and **3^K^** via oxygen atom transfer (OAT) to phosphide **1** (Cp*=C_5_Me_5_).

Heating the terminal phosphide complex **1** in benzene at 70 °C in the presence of pyridine‐*N*‐oxide results in the formation of a new diamagnetic rhenium species, which could be isolated in 77 % yield (Scheme 1).^[15]^ Full conversion of **1** requires two equivalents of the OAT reagent. Intermediates were not observed. Other OAT reagents like Me_3_NO or (IMes)N_2_O (IMes=3‐dimesitylimidazol‐2‐ylidene)^[16]^ led to unselective decomposition. The product exhibits *C*_S_ symmetry on the NMR timescale. The ^31^P NMR signal of the phosphide‐derived fragment (*δ*_P_=246 ppm) is distinctly upfield shifted with respect to parent **1** (*δ*_P_=1069 ppm), close to Cummins’ PO‐complex (Figure 1 d; *δ*_P_=270 ppm).^[12]^ The ^1^H NMR spectrum indicates bidentate κ^2^‐*N* ^1^,*N* ^3^ coordination of the pyrazolpyridine ligand as judged from comparison with **1** vs. [ReX(PyrPz)(PNP)] (X=I, NCO).^[14c]^ These results support the dioxygenation of the phosphide ligand, which is associated with a rearrangement of the hemilabile PyrPz ligand.

Single crystal X‐ray diffraction confirmed phosphide to metaphosphite dioxygenation and crystallization as the dimeric product [(P_2_O_4_){Re(PyrPz)(PNP)}_2_] (**2_2_**; Figure 2). The bridging μ‐*P* ^1^,*P* ^2^‐dimetaphosphite (P_2_O_4_^2−^) ligand exhibits a planar P_2_O_2_ core with P‐O single bonding (1.685(5) Å) and shorter bonds to the terminal oxygen atoms (1.484(5) Å). The bond length of the rhenium ions to the dimetaphosphite P‐atoms (2.4564(18) Å) is significantly elongated with respect to parent **1** (Δ*d*=0.36 Å), in line with reduction of Re‐P triple to single bonding. As in solution, the PyrPz ligand of **2_2_** adopts a κ^2^‐*N* ^1^,*N* ^3^ binding mode, which is attributed to reduced σ‐donation of bridging P_2_O_4_^2−^ vs. P^3−^. P_2_O_4_ was previously observed from P_2_ oxidation in a cryogenic matrix and could be stabilized with N‐heterocyclic carbenes (Figure 1 a).[^6e^, ^9^, ^17^] In contrast, the dimetaphosphite dianion or its nitrogen analog are unknown.

**Figure 2:**
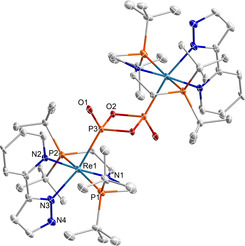
Molecular structure of **2_2_** in the solid state (thermal ellipsoids set at the 50 % probability level); solvent molecules and hydrogen atoms are omitted for clarity. Selected bond lengths [Å] and angles [°]: Re1–N1 1.900(5), Re1–N2 2.263(5), Re1–N3 2.143(6), Re1–P1 2.4457(19), Re1–P2 2.4528(18), Re1–P3 2.4564(18), P3–O1 1.484(5), P3–O2 1.685(5); N1‐Re1‐P3 82.75(16), P1‐Re1‐P2 163.84(16), O2‐P3‐O2# 82.8(2).^[15]^

Interestingly, computational evaluation by density functional theory (DFT) indicated that the equilibrium between monomeric **2** and dimeric **2_2_** (Scheme 1) is close to thermoneutral.[^15^, ^18^] ^31^P NMR chemical shifts were computed to clarify solution speciation. The calculated PO_2_ chemical shift for monomeric **2** (*δ*_P_(PO_2_)=260 ppm, *δ*_P_(PNP)=46 ppm) distinctly differs from dimeric **2_2_** (*δ*_P_(P_2_O_4_)=122 ppm, *δ*_P_(PNP)=43 ppm) and closely resembles the experimental data (*δ*_P_=246 and 13 ppm). Diffusion coefficients obtained by ^31^P{^1^H} DOSY NMR in benzene (*D*_benzene_=7.3×10^−10^ m^2^ s^−1^; *r*_0_=4.9 Å) ^[19]^ and THF (*D*_THF_=8.1×10^−10^ m^2^ s^−1^, *r*_0_=5.9 Å) compare well with related mononuclear complexes, like **1** (*D*_benzene_=7.1×10^−10^ m^2^ s^−1^, *r*_0_=5.1 Å), [ReI(PyrPz)(PNP)] (*D*_benzene_=8.4×10^−10^ m^2^ s^−1^, *r*_0_=4.3 Å) or [ReCl_3_(^H^PNP′)] (*D*_THF_=8.2×10^−10^ m^2^ s^−1^, *r*_0_=5.8 Å; ^H^PNP′=HN(CH_2_CH_2_P*i*Pr_2_)_2_) and significantly differ, for example, from the N_2_ bridged, dinuclear complex [(N_2_){ReCl_2_(^H^PNP′)}_2_] (*D*_THF_=6.4×10^−10^ m^2^ s^−1^, *r*_0_=7.5 Å). Despite the large computed chemical shift difference of the mono‐ and dimetaphosphite ligands (Δ*δ*_P_=138 ppm), the ^31^P NMR spectra of **2** exhibit negligible temperature dependence (Δ*δ*_P_(PO_2_)=8.0 ppm; Δ*δ*_P_(PNP)=5.0 ppm) over a wide range (−80 to +100 °C). Similar invariance was found for variation of the concentration (5–40 mM). The computational and experimental data therefore support that monomeric **2** is the predominant species in solution.

Speciation was further examined by vibrational spectroscopy. The IR spectrum (ATR) of a slowly crystallized, yellowish green solid sample features strong bands at around 550 and 700 cm^−1^ (Figure 3), which can be assigned to in‐plane deformation modes of the P_2_O_4_‐ring of dimeric **2_2_** by comparison with a computed spectrum (DFT: 526, 704 cm^−1^). Dissolving this sample in benzene followed by rapid freezing and sublimation of the solvent gives a brown powder, where these signals are absent. Instead, strong bands at 1079 and 1245 cm^−1^ are observed (Figure 3), which are assigned to the symmetric and asymmetric P‐O stretching vibrations of a terminal monometaphosphite ligand upon comparison with the computational values for **2** (*ν*_s_=1086 cm^−1^, *ν*_as_=1263 cm^−1^) and reported data for free PO_2_^−^ in a KCl matrix (ν_s_=1097 cm^−1^, ν_as_=1207 cm^−1^), respectively.^[6a]^ These results are in line with solution speciation as monomer **2** vs. crystallization as dimer **2_2_**. Notably, a solid sample that was obtained from rapid evaporation of a THF/benzene solution showed both sets of signals for the monomer and the dimer (see ESI, Figure S16). In KBr matrix, only the IR signature for **2** was found (Figure S16). This observation might indicate monomer stabilization by interaction of the PO_2_ ligand with potassium cations, as was found for the anionic rhenium(I) metaphosphite complex **3^K^**, which is presented below.

**Figure 3:**
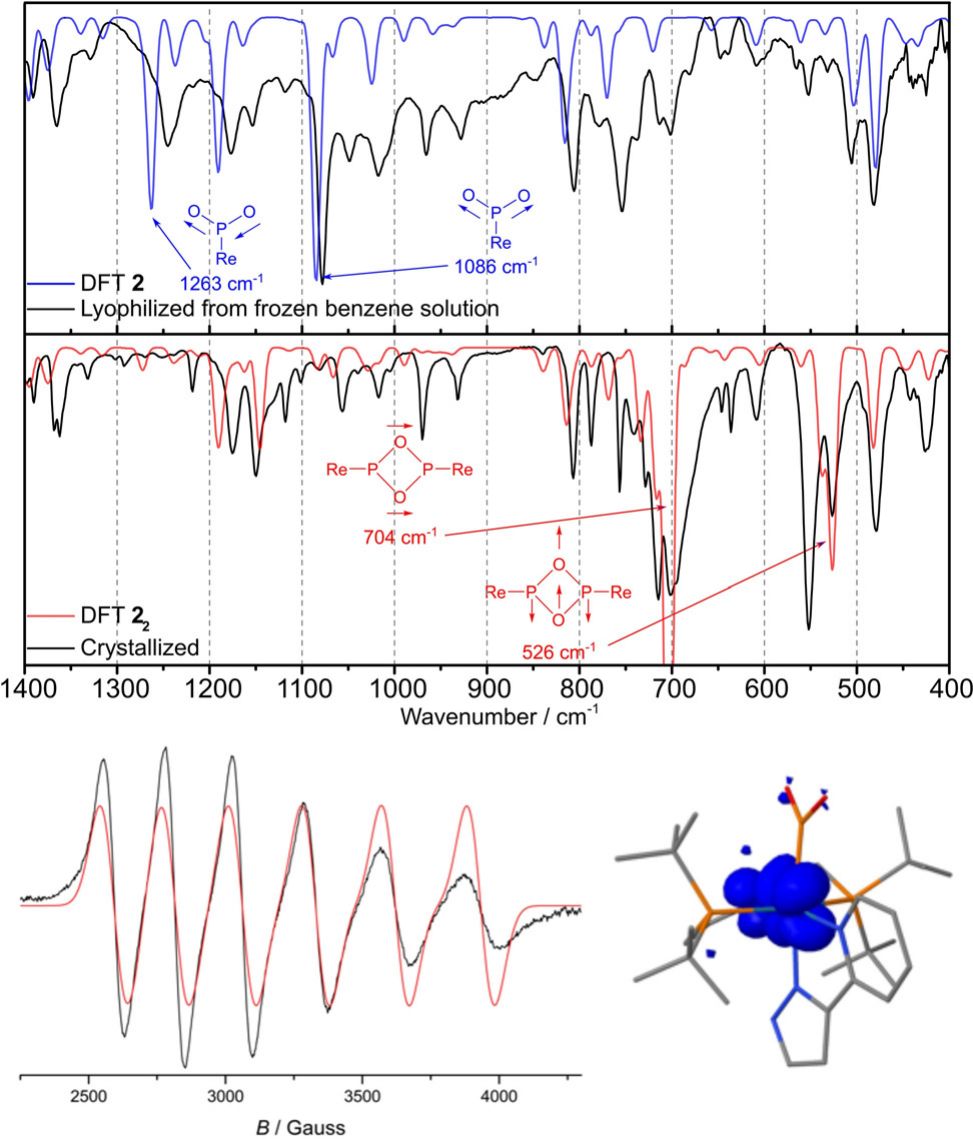
Top: Mid‐IR spectra (ATR) of solid **2** and **2_2_** obtained by lyophilization of a frozen benzene solution or slow crystallization, respectively, and the computed spectra of **2** (blue) and **2_2_** (red) and selected vibrational modes of the {PO_2_} moieties. Bottom left: EPR spectrum of **2^K^** (THF, 300 K, 9.4169 GHz); simulation parameters: *g*_iso_=2.046, *A*_iso_(^185/187^Re)=770 MHz. Bottom right: Computed spin density distribution of **2^−^** (D3BJ‐PBE0/def2‐TZVP).

Electrochemical characterization of **2** by cyclic voltammetry (CV) features a reversible one‐electron reduction at *E*_1_^0^=−1.81 V (potentials are reported vs. FeCp_2_^+/0^).^[15]^ Another reduction at very low potential (*E*_2,pc_=−2.9 V), which is irreversible at all scan rates (*v*=0.05‐3 V s^−1^) and associated with an irreversible feature in the reverse scan at *E*_pa_=−1.57 V, indicates rapid chemical conversion upon overreduction. Controlled potential electrolysis at *E*_1_ over 16 h gave several new features in the CV hinting at slow decomposition of anionic **2^−^**. Chemical reduction was therefore carried out with KC_8_/18‐C‐6 (18‐C‐6=1,4,7,10,13,16‐hexaoxacyclooctadecane) to offer a potentially stabilizing counter cation. A distinct color change from red‐brown to purple was observed and paramagnetic [K(18‐C‐6)Re(PO_2_)(PyrPz)(PNP)] (**2^K^**; Scheme 1) could be isolated in 59 % yield. Near quantitative re‐oxidation of **2^K^** was obtained with [FeCp*_2_][Al{OC(CF_3_)_3_}_4_]. The EPR spectrum of **2^K^** at room temperature in solution (Figure 3) exhibits a 6‐line signal, which could be satisfactorily simulated with *g*_iso_=2.046 and large hyperfine interaction (HFI) with a single low‐spin rhenium(II) ion (*A*_iso_(^185/187^Re)=770 MHz).^[20]^ The absence of resolved ^31^P HFI suggests little spin delocalization onto the metaphosphite ligand.[^21^, ^22^] DFT computations confirm this notion locating the spin density mainly at the rhenium (59 %) and pincer nitrogen (30 %) atoms (Figure 3). This distribution resembles the computed SOMO of **2^−^** and the LUMO of **2** (Figure 5), which are orthogonal to the Re‐PO_2_ σ‐ and π‐interactions (see below) and feature predominant Re‐N π*‐character.

At room temperature, **2^K^** decays over several days in solution, which prevented crystallographic characterization. As one of several products, the rhenium(I) metaphosphite complex **3^K^** (Scheme 1) was obtained with an imine pincer ligand that results from formal hydrogen atom loss. Alternatively, **3^K^** is easily prepared by deprotonation of parent **2** with KO^t^Bu in the presence of 18‐C‐6 (Scheme 1). The ^1^H NMR spectrum of **3^K^** confirms *C*_1_ symmetry on the NMR timescale and the presence of an imine group (*δ*_H_=7.86 ppm). The ^31^P{^1^H} NMR signal at *δ*_P_(PO_2_)=273 ppm indicates that the monometaphosphite ligand is preserved. The solution structural assignment was corroborated by single crystal X‐ray diffraction (Figure 4), which features **3^K^** as contact ion pair with a trigonal‐planar PO_2_^−^ anion that is κ(*P*)‐bound to rhenium and κ(*O*,*O*′)‐coordinated to the potassium counter cation. The P−O bond lengths (1.517(2)/1.512(2) Å) resemble those of free PO_2_^−^ in the gas phase (1.50(1) Å).^[5b]^ Comparison with Lewis‐base stabilized PO_2_^+^ species (1.46–1.47 Å)[^10b^, ^10c^] points at weaker P−O bonding in the anion. In turn, the reported PO_2_^3−^ ligand (Figure 1 c) features one considerably longer, bridging P−O bond (1.58(3) Å).

**Figure 4:**
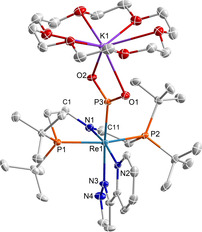
Molecular structure of **3^K^** in the solid state (thermal ellipsoids set at the 50 % probability level); solvent molecules and H atoms are omitted for clarity. Selected bond lengths [Å] and angles [°]: Re1–N1 2.082(2), Re1–N2 2.141(2), Re1–N3 2.162(2), Re1–P1 2.3977(7), Re1–P2 2.4332(7), Re1–P3 2.2545(7), N1–C1 1.468(4), N1–C11 1.327(4), P3–O1 1.517(2), P3–O2 1.512(2), O1–K1 2.730(2), O2–K1 2.731(2); N1‐Re1‐P3 94.31(9), P1‐Re1‐P2 155.70(2), O1‐P3‐O2 110.26(12).^[15]^

The considerably shorter Re‐PO_2_ bond length of **3^K^** (2.2545(7) Å) with respect to dimeric **2_2_** (2.4564(18) Å) reflects the lower phosphorous coordination number but might also indicate some Re→PO_2_ π back‐bonding contribution to the monometaphosphite ligand. For the nitrite homologue, haem‐based nitrite reductase activity was associated with Fe→NO_2_ π back‐bonding in the nitro binding mode.[^3c^, ^3e^, ^23^] Donation into the N‐O π‐antibonding LUMO of NO_2_ results in a redshift of the symmetric NO_2_ stretching vibration of, e.g., around Δ*ν*_s_(NO_2_) ≈60 cm^−1^ for a low‐spin {Fe^III/II^(NO_2_)} redox couple.^[3c]^ The same trend was found for the rhenium(III‐I) metaphosphite complexes **2**/**2^K^**/**3^K^**. Reduction of **2** to **2^K^** is also associated with a bathochromic shift of Δ*ν*_s_(PO_2_)=60 cm^−1^. A smaller shift was found for **2^K^** vs. **3^K^** (Δ*ν*_s_(PO_2_)≤17 cm^−1^). However, the lower formal metal oxidation state coincides with the replacement of a π‐donor (amide) by a competing π‐acceptor (imine) ligand, which precludes direct comparison.

In the MO scheme of **2** (Figure 5), Re→PO_2_ back bonding is reflected by the HOMO‐1, which features contributions with P‐O π‐antibonding character. Bonding of the metaphosphite ligand to the metal was further examined by natural bond orbital (NBO) analysis.[^15^, ^24^] The Re‐PO_2_ interaction is dominated by the σ‐bonding natural localized molecular orbital (NLMO), which is slightly polarized towards phosphorus (**2**: 36 % Re, 61 % P; **2^−^**: 35 % Re, 62 % P), yet more covalent with respect to the computed nitro analogue [Re(κ‐*N*‐NO_2_)(κ‐*N* ^2^‐PyrPz)(PNP)] (**2^NO2^**: 21 % Re, 78 % N). Re→PO_2_ back donation in **2** is expressed by small contributions (approx. 3 %) of the metaphosphite ligand to the NLMOs that represent the two occupied metal d orbitals. The energetic stabilization due to this donor‐acceptor π‐interaction (Δ*E*_π_) can be estimated by second order perturbation theory within the NBO scheme.^[25]^ A cumulative stabilization energy for π donation from the Re lone pairs of **2** into the metaphosphite π‐orbitals of Δ*E*_π_=17 kcal mol^−1^ was obtained. As expected, the free anion **2^−^** exhibits slightly increased Re→PO_2_ back bonding (Δ*E*_π_=23 kcal mol^−1^). For the nitro complex **2^NO2^**, Δ*E*_π_=21 kcal mol^−1^ was computed indicating that the nitro ligand bound to the {Re(PyrPz)(PNP)} fragment is only a slightly stronger π‐acceptor than metaphosphite.

**Figure 5:**
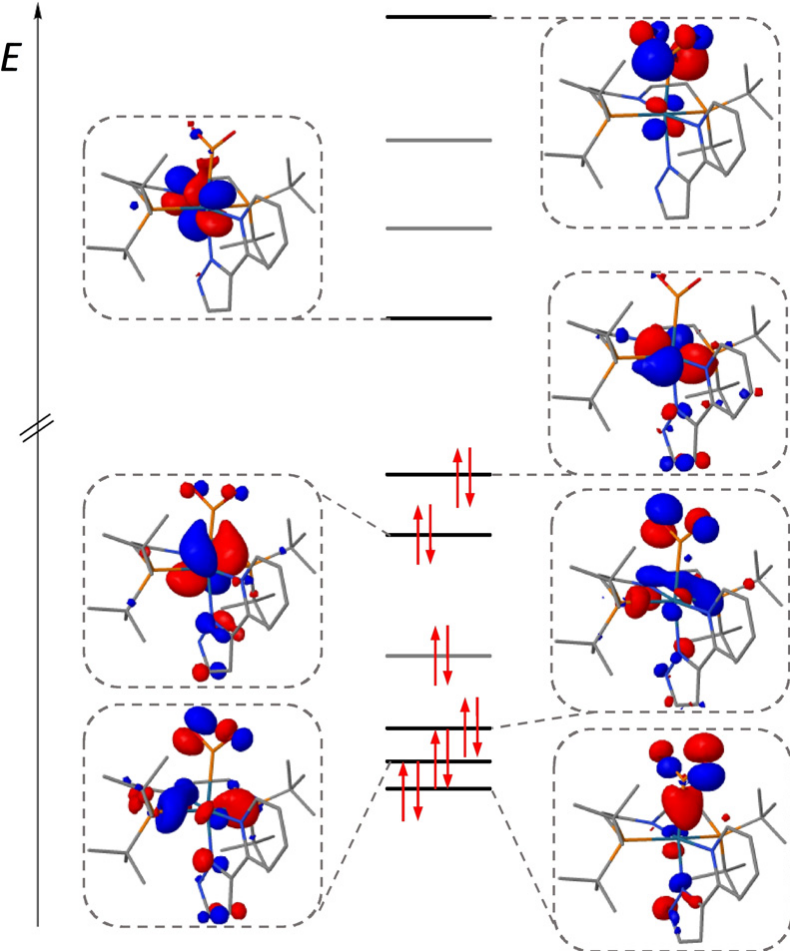
Molecular orbital scheme of **2** (D3BJ‐PBE0/def2‐TZVP). For simplicity, pure pyrazolpyridine ligand based π‐type orbitals are not displayed.

In summary, oxygenation of the terminal rhenium phosphide complex **1** gave access to a series of complexes with the unknown metaphosphite (PO_2_^−^) ligand, that is, the phosphorous analogue of the biologically important nitrite anion. In the solid state, the rhenium(III) compound **2** also exists as dimer **2_2_** with a bridging P_2_O_4_^2−^ dianion. Facile synthesis of rhenium(II) and rhenium(I) complexes by reduction and pincer deprotonation, respectively, demonstrates the chemically robust nature of the metaphosphite ligand. Vibrational spectroscopy data and computational analysis indicate that the P‐bound metaphosphite ligand is a slightly stronger σ‐donor and comparable π‐acceptor ligand compared with the nitrite homologue.

## Conflict of interest

The authors declare no conflict of interest.

## Supporting information

As a service to our authors and readers, this journal provides supporting information supplied by the authors. Such materials are peer reviewed and may be re‐organized for online delivery, but are not copy‐edited or typeset. Technical support issues arising from supporting information (other than missing files) should be addressed to the authors.

Supplementary
