# Supplementary material for: The Metaphosphite (PO2 −) Anion as a Ligand
Source: Angew Chem Int Ed Engl. 2020 Oct 25;59(52):23574–8. doi: 10.1002/anie.202011750 (PMC7756739; doi:10.1002/anie.202011750)
Supplement: Supplementary file 1 — Supplementary [file ANIE-59-23574-s001.pdf]

Angewandte Chemie
Eine Zeitschrift der Gesellschaft Deutscher Chemiker

Supporting Information

The Metaphosphite (PO_2^-) Anion as a Ligand

*Josh Abbenseth[+], Florian Wätjen[+], Markus Finger, and Sven Schneider**

anie_202011750_sm_miscellaneous_information.pdf

Table of Contents

Materials and Methods 2
Syntheses 2
Spectroscopic Results 4
Determination of the Diffusion Constant of **2** 12
Controlled potential electrolysis of **2** 17
Crystallographic Details 18
X-ray Single-Crystal Structure Analysis of $\mathbf{2_2}$ 18
X-ray Single-Crystal Structure Analysis of $\mathbf{3^K}$ 22
DFT Calculations 23
Computational Details 23
Computed geometries and electronic properties 24
Thermodynamics of the equilibrium of **2** and $\mathbf{2_2}$ 29
Computed IR and NMR Data of **2**, $\mathbf{2_2}$, $\mathbf{2^K}$ and $\mathbf{3^K}$ 30
IR 30
NMR 32
XYZ coordinates 33
References 45

Materials and Methods

All experiments were carried out using Schlenk (argon atmosphere) and glovebox (argon atmosphere) techniques. All solvents were dried by passing through columns packed with activated alumina. Deuterated solvents were obtained from Euriso-Top GmbH, dried over Na/K distilled by trap-to-trap transfer *in vacuo*, and degassed by three freeze–pump–thaw cycles, respectively. Silica gel 60 silanized was purchased from Merck KGaA and heated at 120°C *in vacuo* for 5 days prior to use. Pyridine-*N*-oxide (TCI) and $[Fe(C_5Me_5)_2]$ (abcr) were sublimed prior to use. 18-crown-6 (Sigma Aldrich) was recrystallized prior to use. $Ag[Al(O(C(CF_3)_3)_4]$ (Iolitec) was used as purchased. KC_8, [ReI(κ^2-*N*^{1},*N*^{3}-PyrPz)(PNP)] and **1** were synthesized according to literature procedures.[1] **Elemental analyses** were obtained from the analytical laboratories at the Georg-August University on an Elementar Vario EL 3. **NMR spectra** were recorded with Bruker Avance III HD 500 and a Bruker Avance Neo 400 spectrometers and were calibrated to the residual solvent proton resonance (C_6D_6 δ_H = 7.16 ppm, THF-d_8 δ_H = 3.58 ppm). **Cyclic voltammograms (CVs) and electrolysis experiments** were carried out with a GAMRY 1010E potentiostat using Ag/Ag$^+$ reference-, glassy-carbon working- and Pt-wire counter electrodes. The CVs were referenced to the $[Fe(C_5H_5)_2]^+/[Fe(C_5H_5)_2]$ redox couple, using $[Fe(C_5Me_5)_2]$ as internal reference (E_0 = –0.427 V).[2] **EPR spectra** were recorded on a Bruker ELEXSYS-II E500 CW-EPR spectrometer and simulated with EasySpin. **IR spectra** were measured in the solid state (neat) or KBr pellet using a BRUKER ALPHA FT-IR spectrometer with Platinum ATR module or transmission module, respectively.

Syntheses

Synthesis of 2. Complex **1** (10 mg, 14 µmol, 1.0 eq) and pyridine-N-oxide (2.6 mg, 28 µmol, 2.0 eq) are dissolved in benzene (0.5 ml) and heated to 70°C for 2 h. After cooling to room temperature, the solution is filtered off and the residue is extracted with benzene (2 x 0.5 mL). The volume is reduced to 0.5 mL and the crude product is purified by column chromatography over silanized silica (C_6H_6/THF). The solvent is removed and the residue is extracted with benzene. Evaporation of the solvent and subsequent lyophilization yields **2** as a red powder (8.1 mg, 10.8 µmol, 77%).

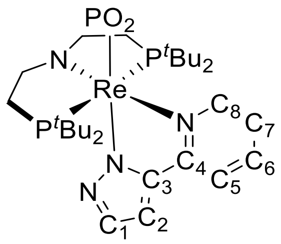

Anal. Calcd for $C_{28}H_{50}N_4O_2P_3Re$ (753.9): C, 44.6; H, 6.69; N, 7.43. Found: C, 44.8; H, 6.80; N, 7.33. NMR (C_6D_6, 25°C): ^{1}H (500 MHz): δ = 11.3 (d, $^3J_{HH}$ = 5.9 Hz, 1H, C_8H), 7.77 (d, $^3J_{HH}$ = 1.6 Hz, 1H, C_1H), 7.33 (ddd, $^3J_{HH}$ = 8.0 Hz, $^4J_{HH}$ = 1.5 Hz, $^5J_{HH}$ = 0.8 Hz, 1H, C_5H), 6.81 (ddd, $^3J_{HH}$ = 7.5 Hz, $^3J_{HH}$ = 6.0 Hz, $^4J_{HH}$ = 1.5 Hz, 1H, C_7H), 6.42 (ddd, $^3J_{HH}$ = 7.5 Hz, ^3J = 6.0 Hz, ^4J = 1.5 Hz, 1H, C_6H), 6.36 (m, $^3J_{HH}$ = 1.8 Hz, 1H, C_2H), 3.74 - 3.38 (m, 4H, NCH_2), 2.28 - 2.08 (m, 4H, PCH_2), 1.23 ($A_9XX'A_9'$, $N = |^2J_{AX} + {}^4J_{AX}|$ = 12.6 Hz, 18H, $P(C(CH_3)_3)_2$), 0.51 ($A_9XX'A_9'$, $N = |^2J_{AX} + {}^4J_{AX}|$ = 12.1 Hz, 18H, $P(C(CH_3)_3)_2$). $^{13}C\{^1H\}$ (125.8 MHz): δ = 155.8 (s, 1C, C_8), 150.7 (d, $^3J_{CP}$ = 2.8 Hz, 1C, C_4), 149.1 (d, $^3J_{CP}$ = 4.3 Hz, 1C, C_3), 143.1 (d, $^4J_{CP}$ = 4.1 Hz, 1C, C_1), 138.6 (s, 1C, C_6), 117.9 (s, 1C, C_7), 115.9 (s, 1C, C_5), 104.4 (d, $^4J_{CP}$ = 1.2 Hz, 1C, C_2), 96.6 (vt, $N = |^2J_{PC} + {}^3J_{PC}|$ = 7.6 Hz, $^3J_{CP}$ = 1.0 Hz, 2C, NCH_2), 41.5 (vt, $N = |^1J_{CP} + {}^3J_{CP}|$ = 18.9 Hz, 2C, $P(C(CH_3)_3)_2$), 40.7 (vt, $N = |^1J_{CP} + {}^3J_{CP}|$ = 14.2 Hz, 2C, $P(C(CH_3)_3)_2$), 31.1 (vt, $N = |^2J_{CP} + {}^4J_{CP}|$ = 3.9 Hz, 6C, $P(C(CH_3)_3)_2$), 29.6 (vt, $N = |^2J_{CP} + {}^4J_{CP}|$ = 4.5 Hz, 6C, $P(C(CH_3)_3)_2$), 28.2 (vt, $N = |^1J_{PC} + {}^3J_{PC}|$ = 15.3 Hz, 2C, PCH_2). $^{31}P\{^1H\}$ (204.5 MHz) δ = 245.7 (t, $^2J_{PP}$ = 20.7 Hz, 1P; PO_2), 13.2 (d, $^2J_{PP}$ = 20.7 Hz, 2P, $P(C(CH_3)_3)_2$).

Synthesis of 2^K. Complex **2** (10 mg, 13 µmol, 1.0 eq) and 18-crown-6 (3.5 mg, 13 µmol, 1.0 eq) are dissolved in THF and cooled to –80°C. KC_8 (2.0 mg, 14.6 µmol, 1.1 eq) is added in one portion and the solution is slowly allowed to warm to room temperature. After stirring for 30 minutes at room temperature the solution is filtered off. The solvent is removed *in vacuo* and the residue is extracted with benzene (3 x 1 mL). The solvent is removed *in vacuo* and the product is crystallized from toluene/pentane at –80°C for 3 days. The supernatant is decanted off and the residue is extracted with benzene. Lyophilization affords **2^K** as of a purple powder (8.3 mg, 7.9 µmol, 59%).
Anal. Calcd for $C_{40}H_{74}KN_4O_8P_3Re$ (1057.3): C,45.4; H, 7.06; N, 5.30. Found: C, 45.8; H, 7.23; N, 5.28. NMR (C_6D_6, 25°C): ^{1}H (300 MHz): δ = 3.25 (s, br, 24H, OCH_2), 2.52 - −0.15 (br).

Synthesis of $[Fe(C_5Me_5)_2][Al(O(C(CF_3)_3)_4]$. $[Fe(C_5Me_5)_2]$ (25.0 mg, 76.9 µmol, 1.00 eq.) and $Ag[Al(O(C(CF_3)_3)_4]$ (80.0 mg, 74.2 µmol, 0.97 eq.) are dissolved in DCM (3 mL) at –0°C and stirred for 30 minutes. The solution is filtered, concentrated *in vacuo* and layered with pentane (10 mL). After crystallization at –35°C for 1 day the precipitate is filtered off, washed with pentane (3 x 1 mL) and extracted with THF (3 x 1 mL). The solvent is removed and $[Fe(C_5Me_5)_2][Al(O(C(CF_3)_3)_4]$

(78.0 mg, 59.7 µmol, 78%) is obtained as a turquoise powder. Anal. Calcd for $C_{36}H_{30}AlF_{36}FeO_4$ (1293.4): C, 33.4; H, 2.34. Found: C, 33.6; H, 2.16.

Oxidation of 2^K^. Complex **2^K^** (8.1 mg, 7.7 µmol, 1.0 eq.) and $[Fe(C_5Me_5)_2][Al(O(C(CF_3)_3)_4]$ (9.9 mg, 7.7 µmol, 1.0 eq.) are placed in a *J*-Young NMR tube. THF-d_8 (0.5 mL) is added, the solution is shaken for 1 minute and analyzed by NMR spectroscopy. The yield of **2** was calculated from the relative ratios of **2** and $[Fe(C_5Me_5)_2]$.

Synthesis of 3^K. Complex **2** (7.0 mg, 9.3 µmol, 1.0 e) and 18-crown-6 (2.5 mg, 9.3 µmol, 1.0 eq) are mixed in benzene (1 mL) and stirred for 5 min. Then KO^{t}Bu (1.0 mg, 9.3 µmol, 1.0 eq) is added and the reaction is stirred for 20 min at RT. The deep red solution is evaporated *in vacuo* to dryness and the crude product is washed with pentane (3 x 1 mL) and extracted with Et_2O (4 x 0.5 mL). Removal of the solvent and lyophilization from benzene yields **3^K** as deep red powder (6.8 mg, 69 %).

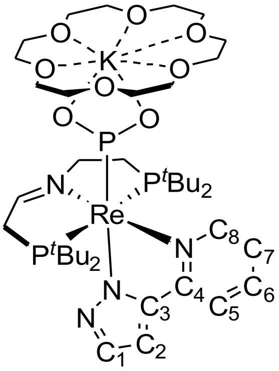

Anal. Calcd for $C_{40}H_{73}KN_4O_8P_3Re$ (1056.3): C, 45.48; H, 6.97; N, 5.30. Found: C, 45.78; H, 7.05; N, 5.52. NMR (THF-d_8, 25°C): ^{1}H (400 MHz): δ = 11.62 (d, $^3J_{HH}$ = 6.2 Hz, 1H, C_8H), 7.86 (m, $^3J_{HP}$ = 16.8 Hz, 1H, N=C*H*-), 7.31 (d, $^3J_{HH}$ = 1.9 Hz, 1H, C_1H), 7.19 (dd, $^3J_{HH}$ = 7.8 Hz, $^4J_{HH}$ = 1.7 Hz, 1H, C_5H), 6.77 (dd, $^3J_{HH}$ = 7.4 Hz, $^4J_{HH}$ = 1.4 Hz, 1H, C_6H), 6.40 (d, $^3J_{HH}$ = 1.9 Hz, 1H, C_2H), 6.34 (dd, $^3J_{HH}$ = 6.6 Hz, $^4J_{HH}$ = 1.8 Hz, 1H, C_2H), 4.09-3.89 (m, 2H, superimposed NCH_2 and CH-CH_2), 3.85-3.65 (m, 1H, NCH_2), 3.65 (s, 24H, O-CH_2), 3.03 (m, 2JHP = 9.7 Hz, 1H, CH_2-CH), 2.97 – 2.85 (m, 1H, PCH_2-CH_2), 2.07 – 1.96 (m, 1H, PCH_2-CH_2), 1.37 (d, $^3J_{HP}$ = 10.7 Hz, 6H, $P(C(CH_3)_3)$), 1.09 (d, $^3J_{HP}$ = 10.7 Hz, 6H, $P(C(CH_3)_3)$), 0.55 (d, br, $^3J_{HP}$ = 10.4 Hz, 6H, $P(C(CH_3)_3)$), 0.45 (d, $^3J_{HP}$ = 11.2 Hz, 6H, $P(C(CH_3)_3)$). $^{13}C\{^1H\}$ (100.7 MHz): δ = 157.5 (s, 1C, C_8), 154.0 (s, 2C, superimposed N=*C*H, C_3), 138.7 (s, 1C, C_4), 138.7 (s, 1C, C_1), 126.6 (s, 1C, C_6), 117.8 (s, 1C, C_7), 117.2 (s, 1C, C_5), 102.2 (s, 1C, C_2), 71.2 (s, 12C, OCH_2), 66.7 (s, 1C, NCH_2), 42.1 (d, $^1J_{CP}$ = 13.5 Hz, 1C, $PC(CH_3)_3$), 40.6 (d, $^1J_{CP}$ = 11.5 Hz, 1C, $PC(CH_3)_3$), 39.4 (s, 1C, $PC(CH_3)_3$), 38.7 (d, 1JCP = 8.7 Hz, 1C, PCH_2CH), 32.6 (d, $^2J_{CP}$ = 4.2 Hz, 1C, $PC(CH_3)_3$), 30.9 (d, $^2J_{CP}$ = 4.8 Hz, 1C, $PC(CH_3)_3$), 30.6 (s_{br}, 1C, $PC(CH_3)_3$), 30.3 (d, $^2J_{CP}$ = 3.3 Hz, 1C, $PC(CH_3)_3$), 28.5 (d, $^1J_{CP}$ = 18.9 Hz, 1C, PCH_2CH_2). $^{31}P\{^1H\}$ (162.0 MHz) δ = 273.2 (t, $^2J_{PP}$ = 27.1 Hz, 1P; PO_2), 63.8 (dd, $^2J_{PP}$ = 200.8, 28.4 Hz, 1P, $P(C(CH_3)_3)_2$), 48.2 (dd, $^2J_{PP}$ = 200.8, 25.4 Hz, 1P, $P(C(CH_3)_3)_2$).

Spectroscopic Results

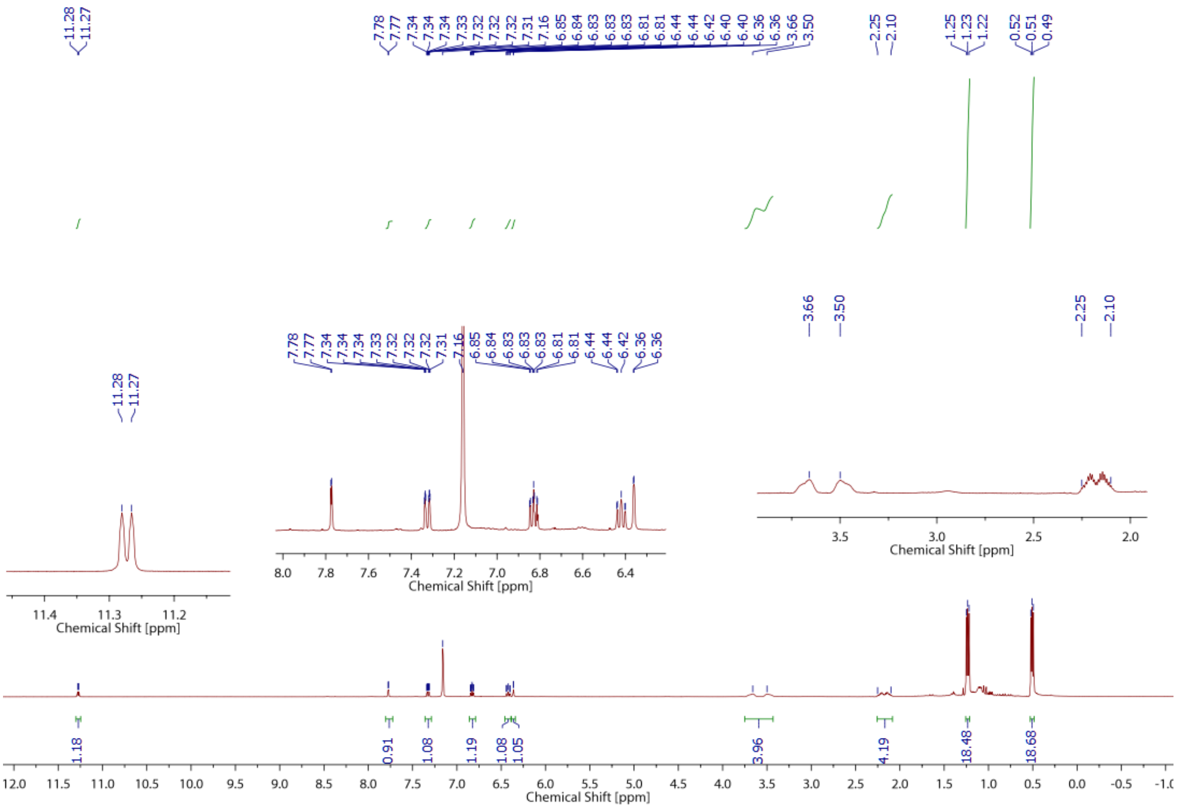

Figure S1. ^{1}H NMR spectrum of complex **2** in C_6D_6 at room temperature.

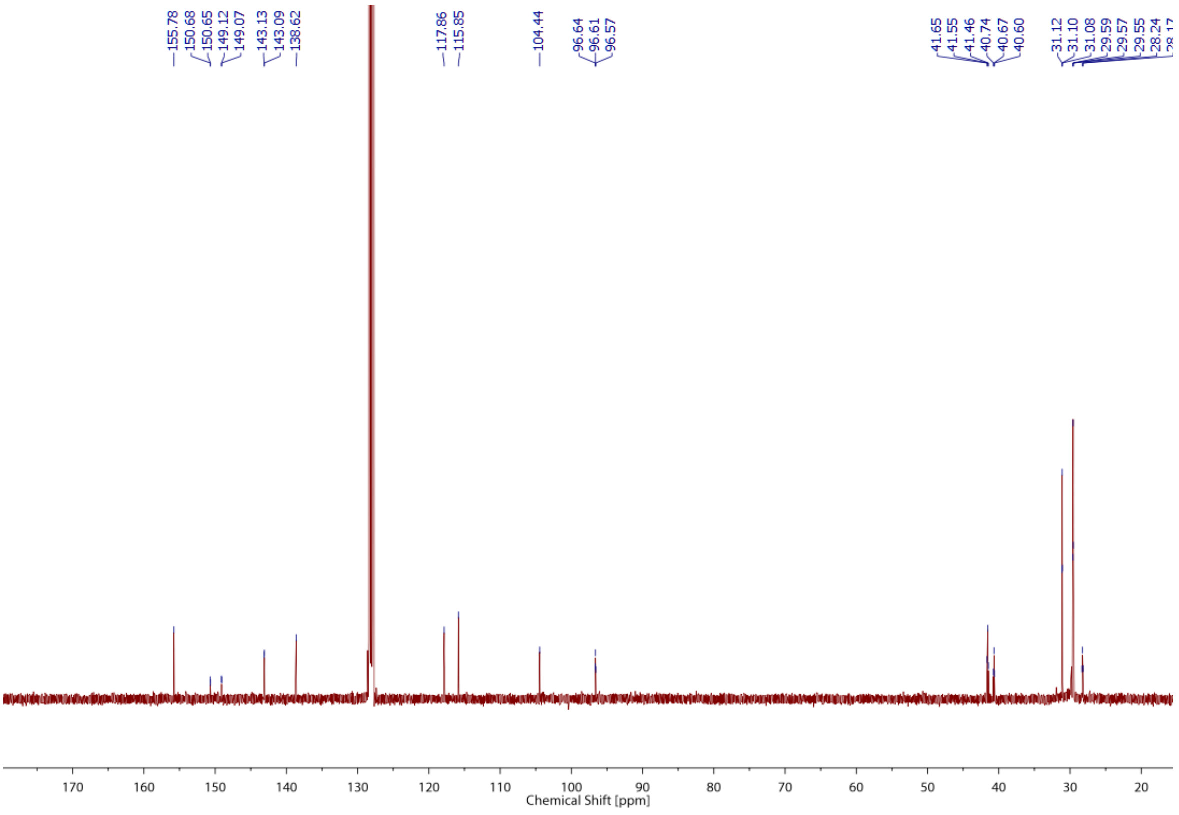

Figure S2. $^{13}C\{^{1}H\}$ NMR spectrum of complex **2** in C_6D_6 at room temperature.

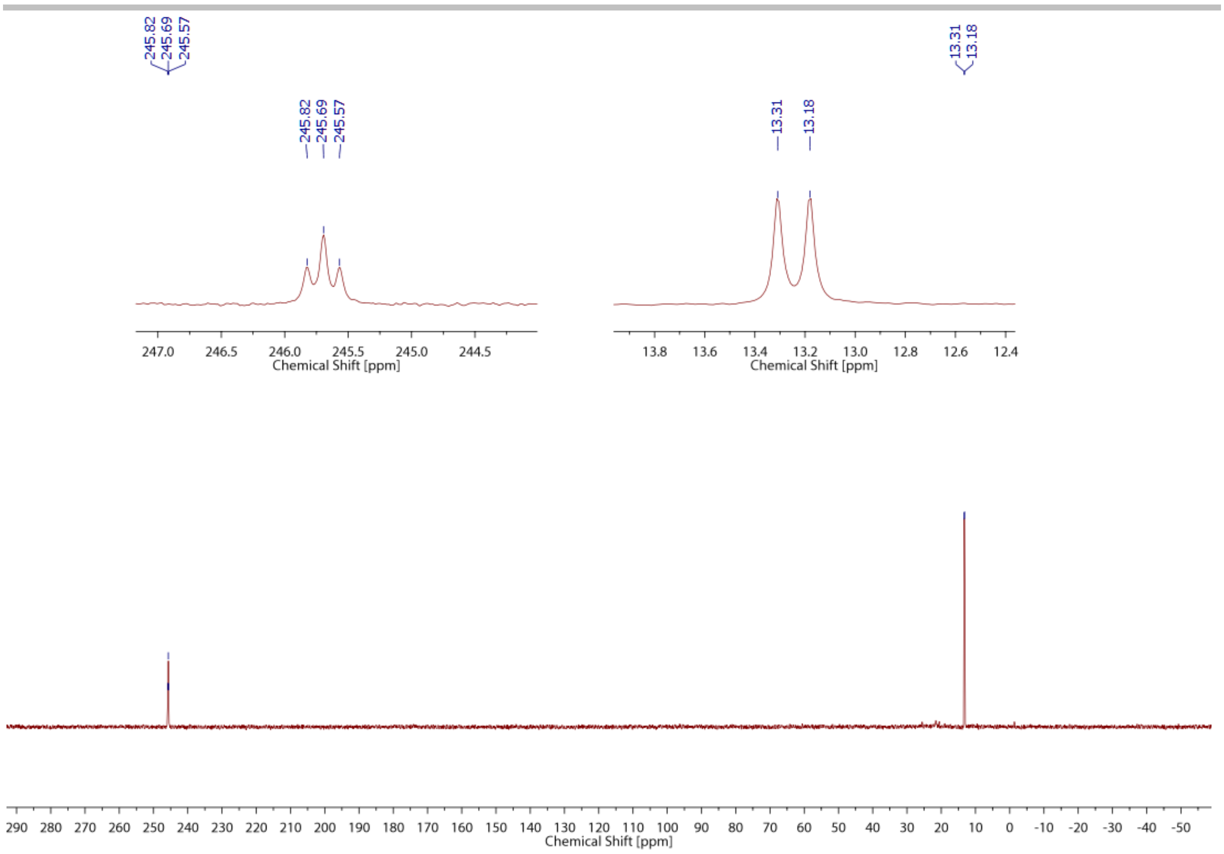

Figure S3. $^{31}P\{^{1}H\}$ NMR spectrum of complex **2** in C_6D_6 at room temperature.

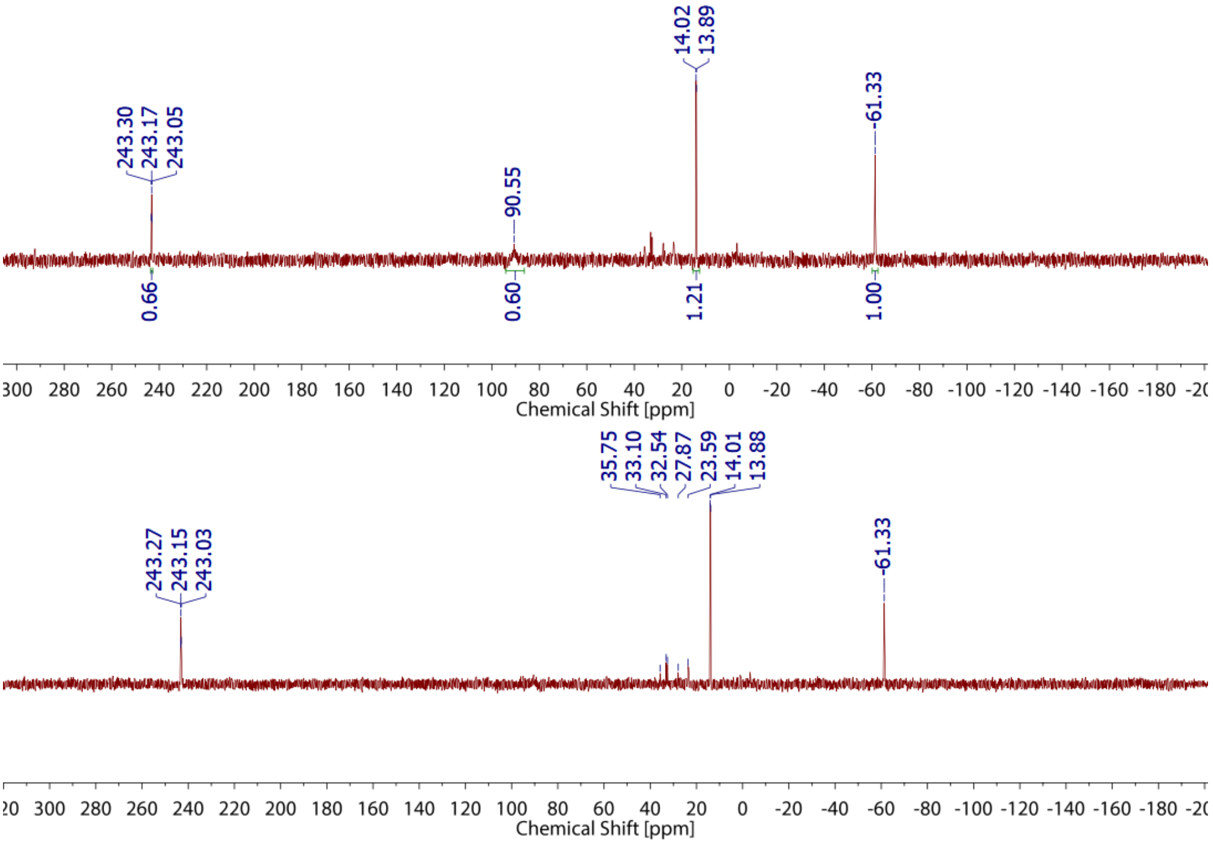

Figure S4. $^{31}P\{^{1}H\}$ NMR spectra of the reaction of complex **1** with pyridine-*N*-oxide (C_6D_6, 70°C) during the reaction (top) and after full conversion (bottom). PMe_3 (δ_P = –61.3 ppm) was used as internal standard in a sealed capillary.

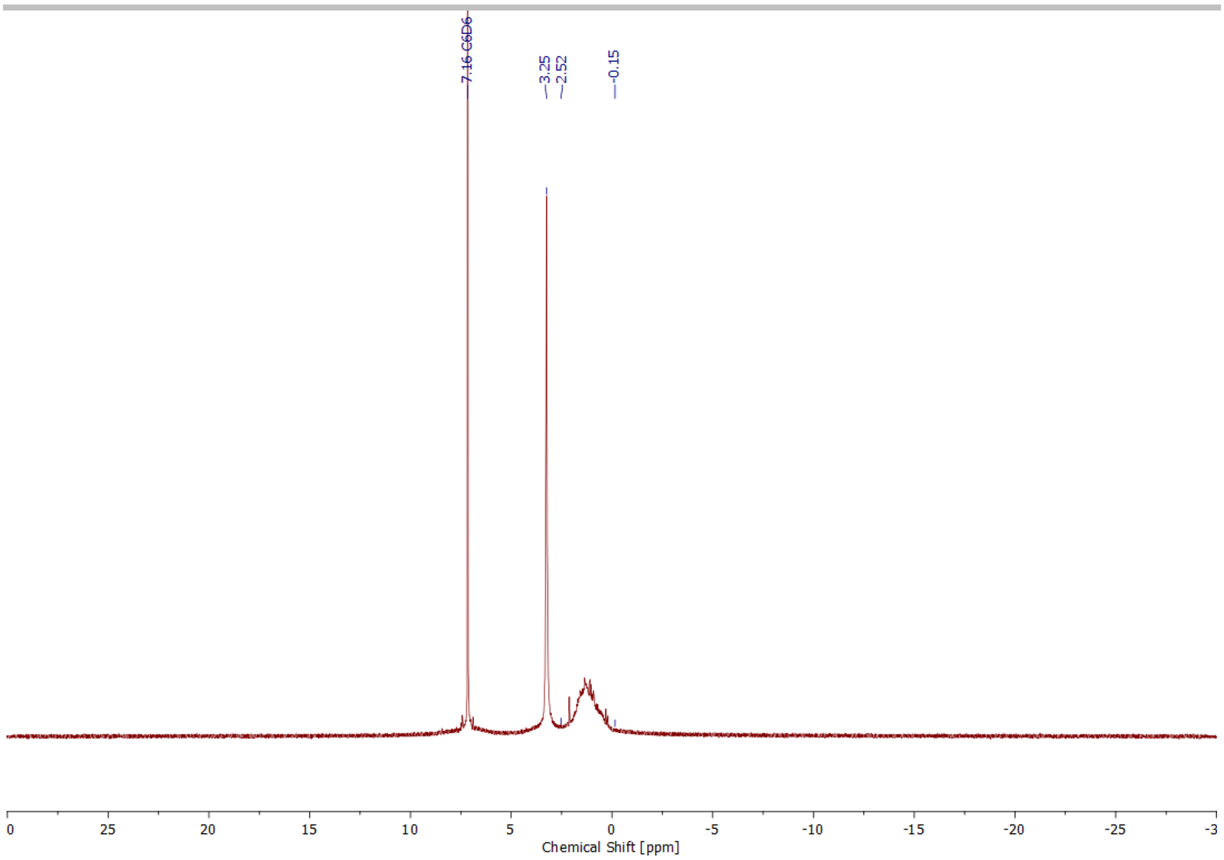

Figure S5. ^{1}H NMR spectrum of complex $\mathbf{2^K}$ in C_6D_6 room temperature.

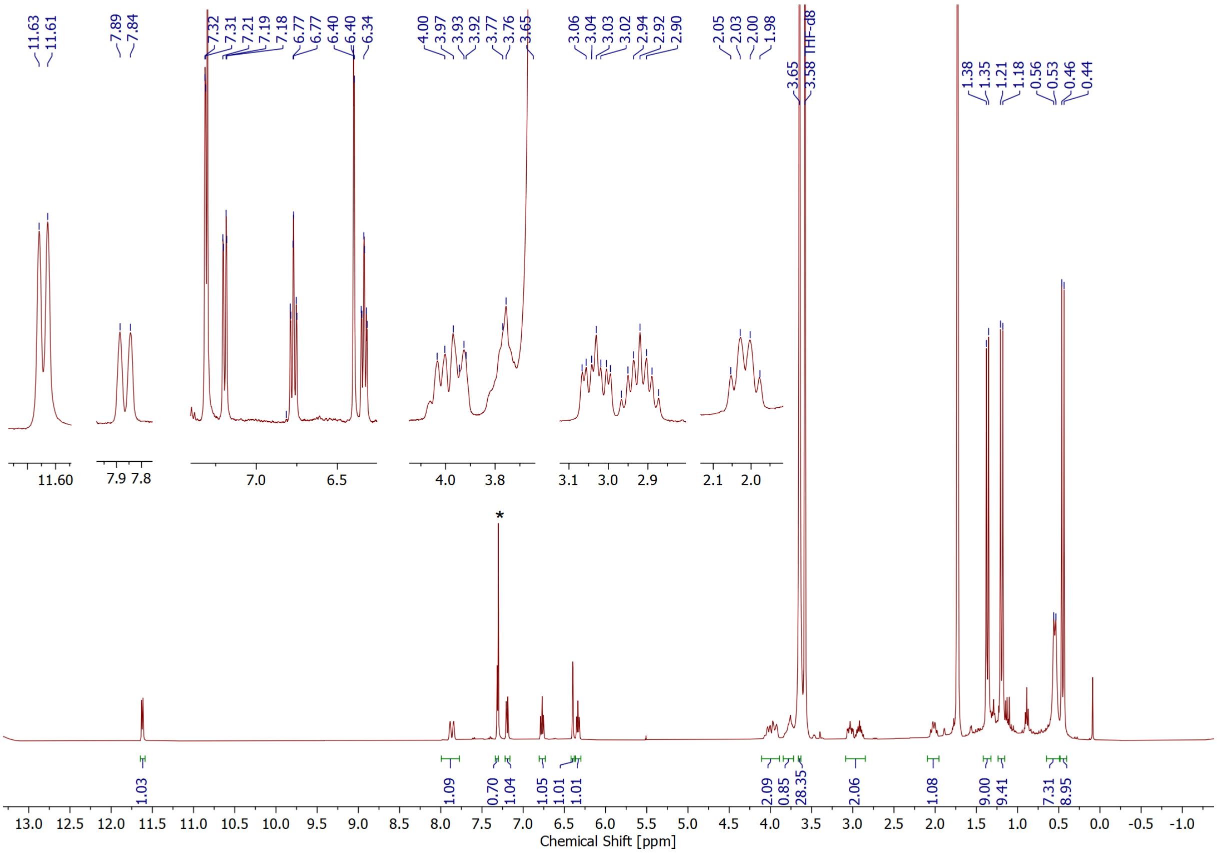

Figure S6. ^{1}H NMR spectrum of complex $\mathbf{3^K}$ in THF-d_8 at room temperature.

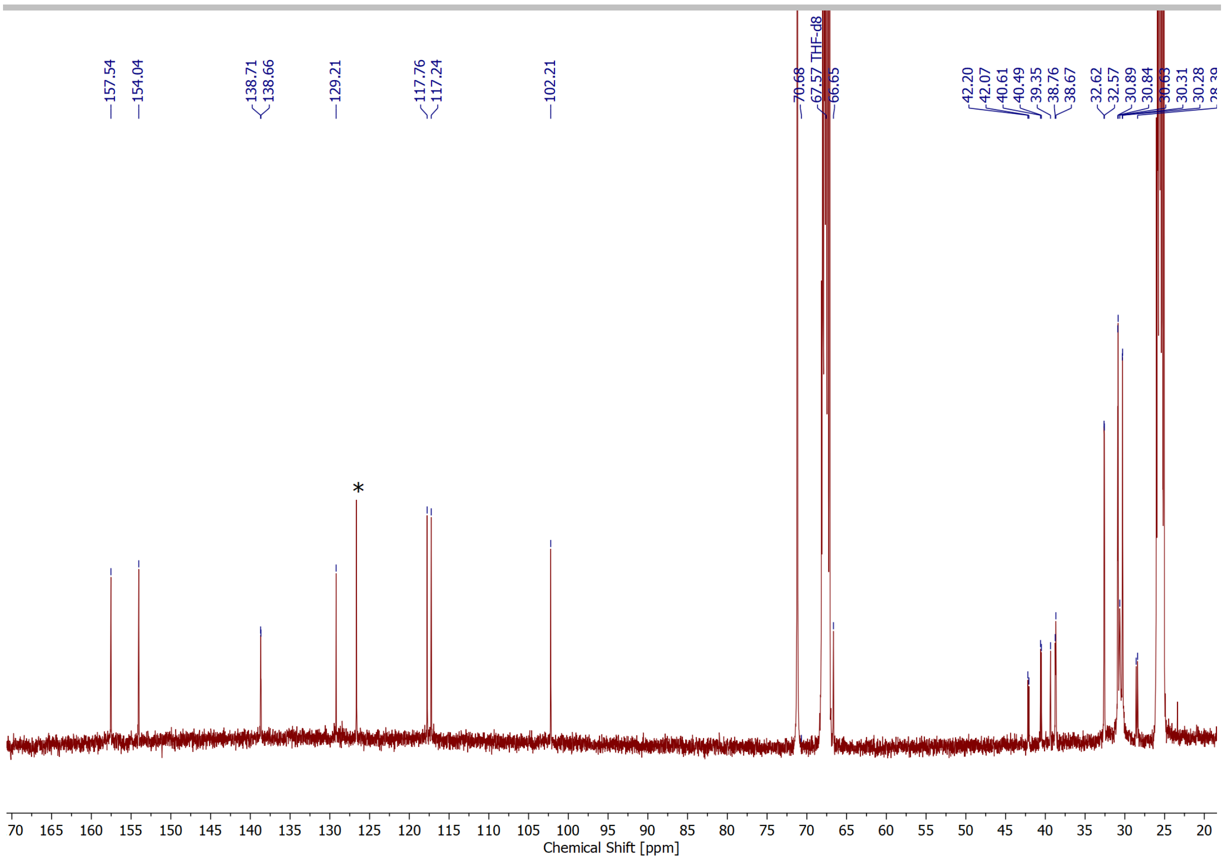

Figure S7. $^{13}C\{^{1}H\}$ NMR spectrum of complex **3^{K}** in THF-d_8 at room temperature.

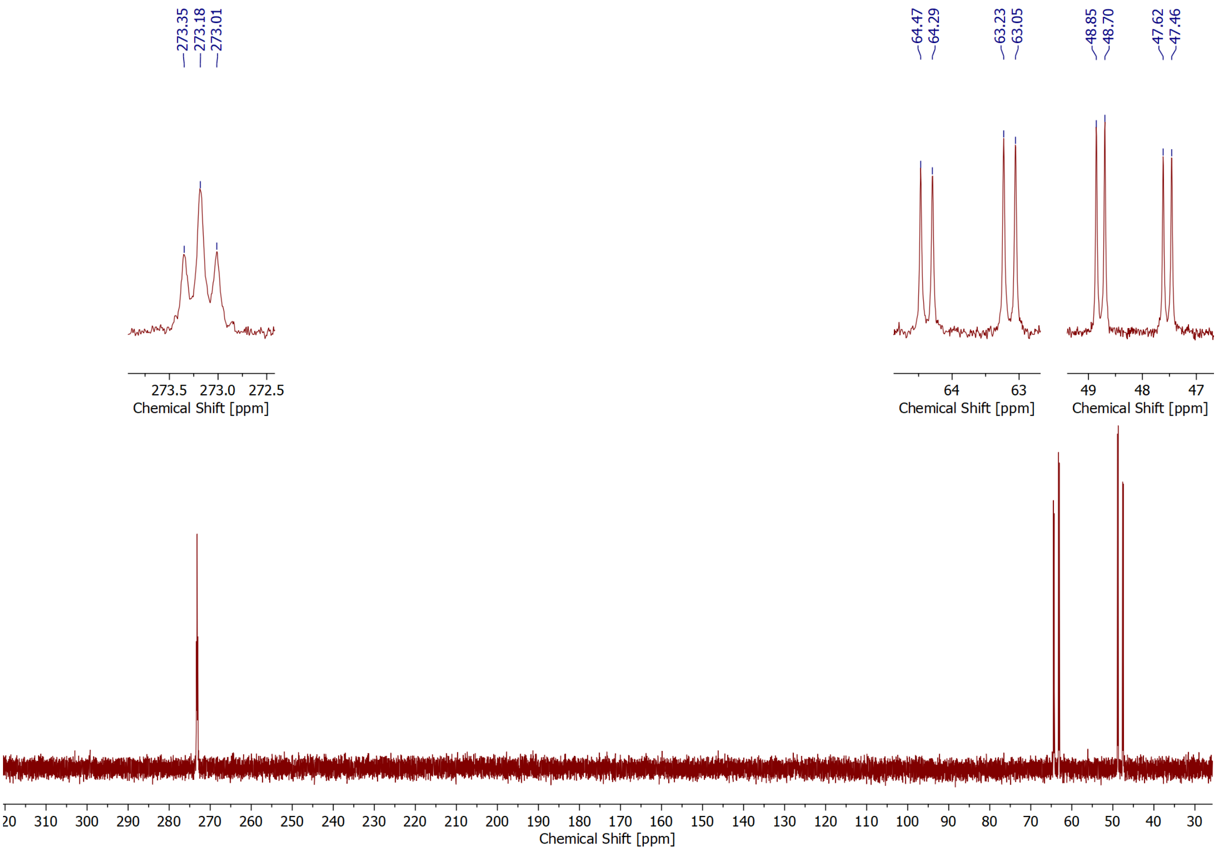

Figure S8. $^{31}P\{^{1}H\}$ NMR spectrum of complex **3^{K}** in THF-d_8 at room temperature.

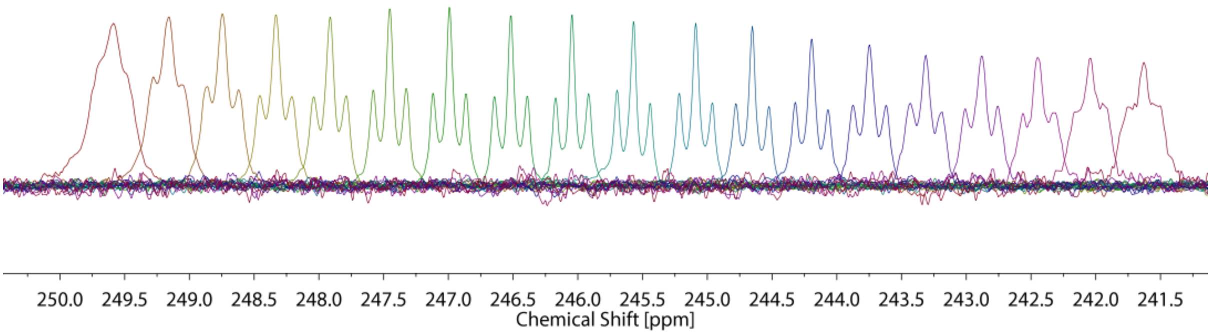

Figure S9. Superimposed VT $^{31}P\{^1H\}$ NMR spectra of complex **2** (PO_2^- signal) in toluene over the temperature range of –80 °C (left) to +100 °C (right) using PPh_3O as internal standard (sealed capillary, not shown); $\Delta\delta_P$ = 7.97 ppm.

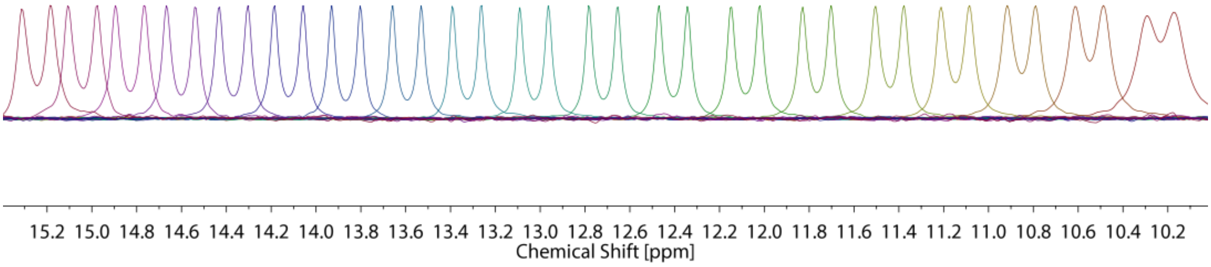

Figure S10. Superimposed $^{31}P\{^1H\}$ NMR spectra of complex **2** (PNP signal) in toluene over the temperature range–80°C (left) to +100°C (right) using PPh_3O as internal standard (sealed capillary, not shown); $\Delta\delta_P$ = 5.01 ppm.

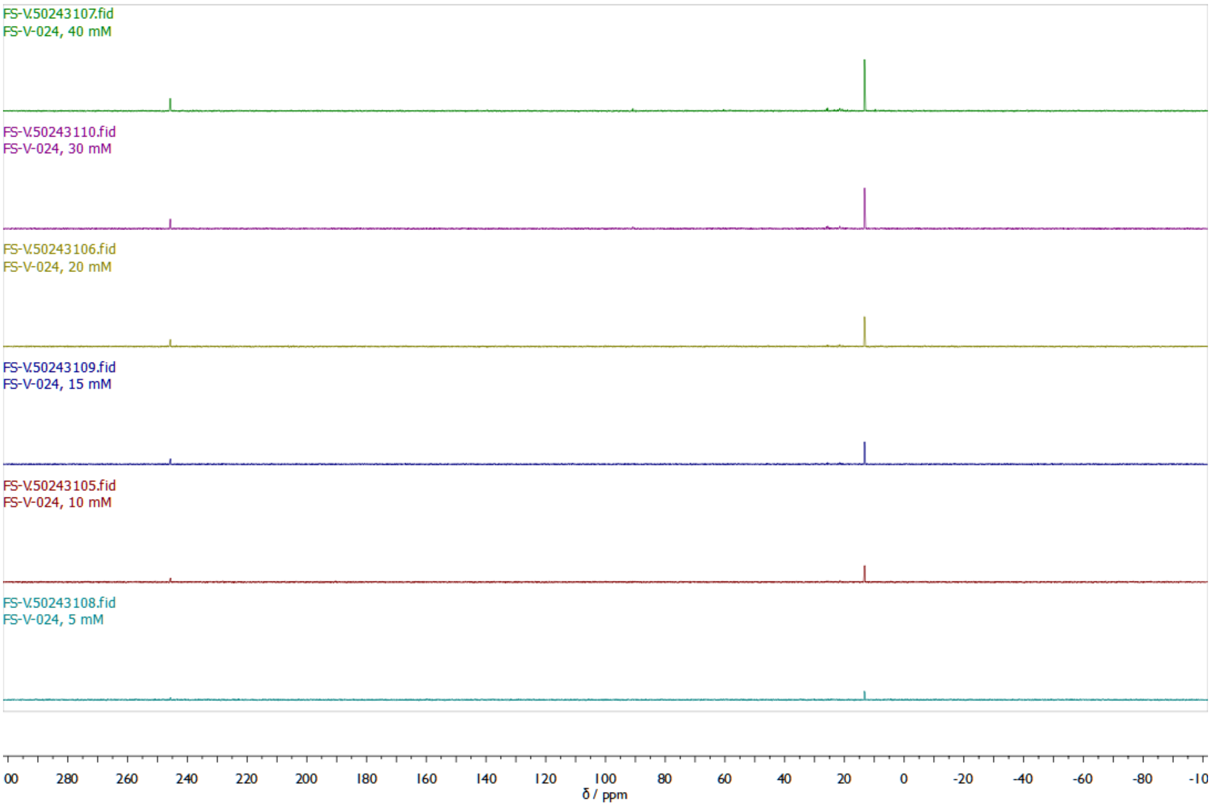

Figure S11. Concentration dependent $^{31}P\{^1H\}$ NMR spectra of complex **2** in C_6D_6 at room temperature.

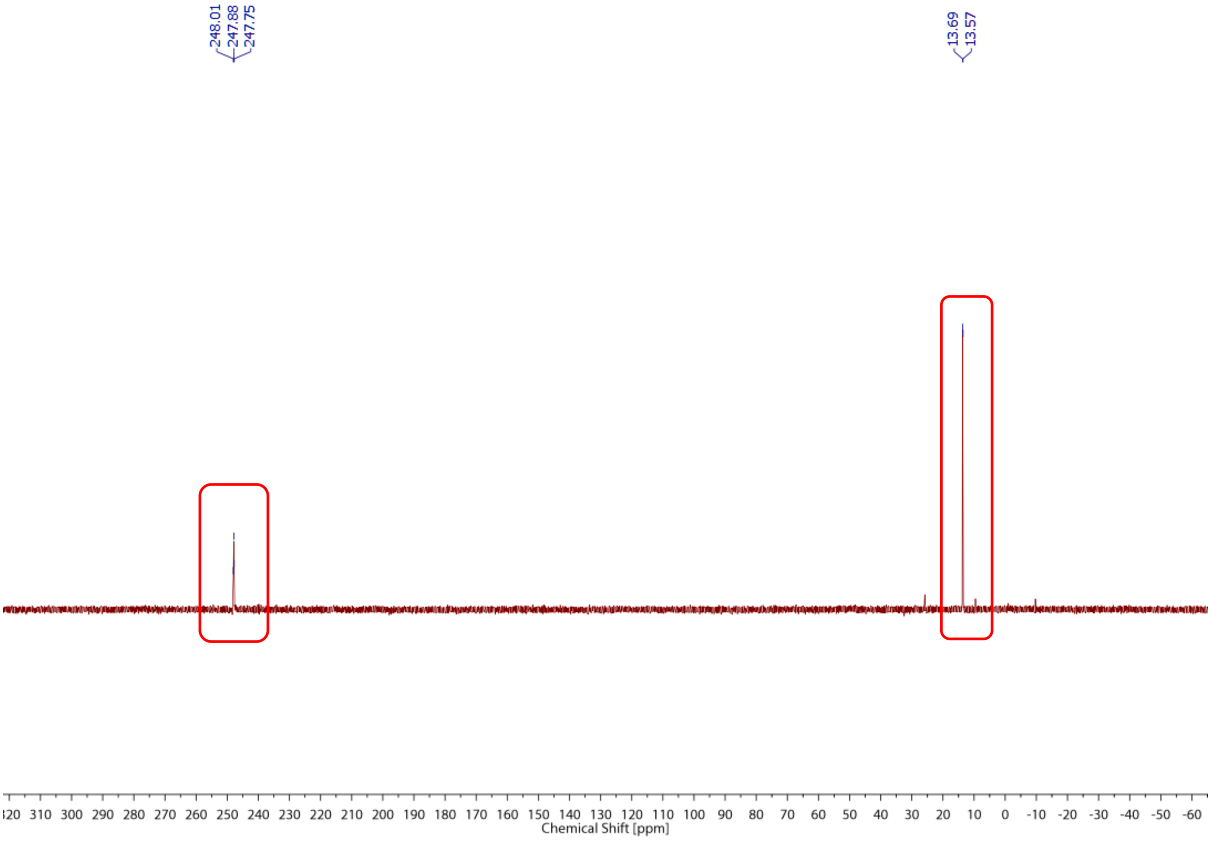

Figure S12. $^{31}P\{^1H\}$ NMR spectrum of the oxidation of complex $\mathbf{2}^{K}$ with $[Fe(C_5Me_5)_2][Al(O(C(CF_3)_3)_4]$ in THF at room temperature.

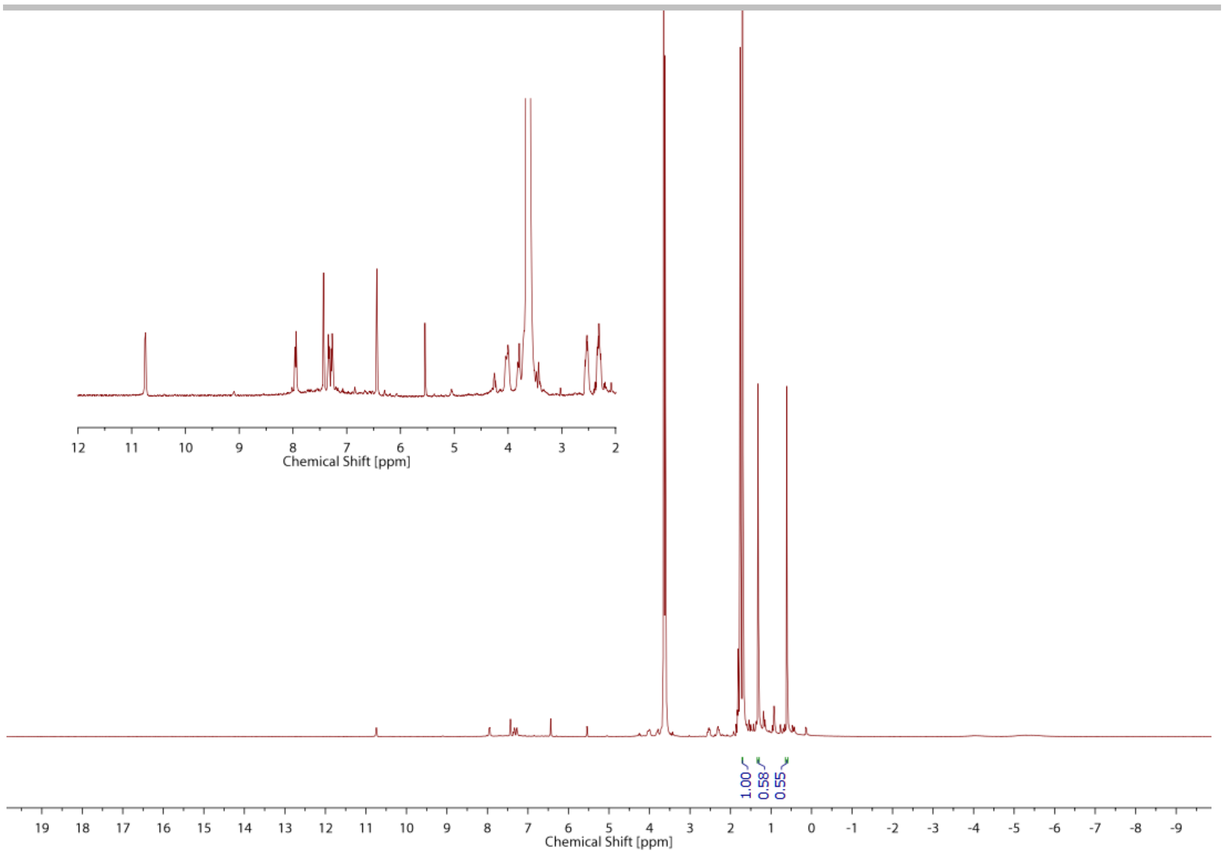

Figure S13. $^{1}H\{^{31}P\}$ NMR spectrum of the oxidation of complex **2**^{K} with $[Fe(C_5Me_5)_2][Al(O(C(CF_3)_3)_4]$ in THF–d_8 at room temperature.

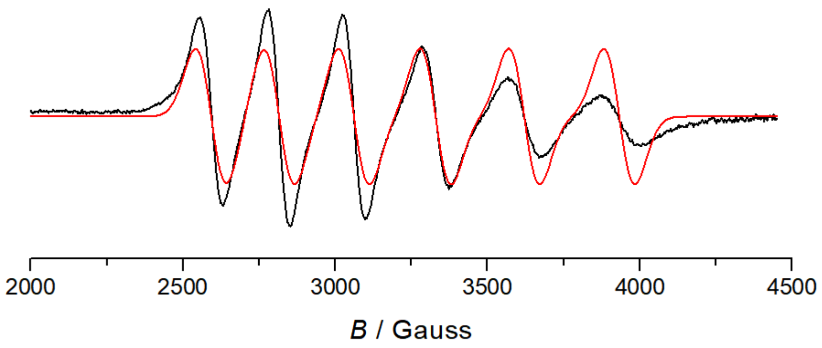

Figure S14. CW X-band EPR spectrum of complex **2**^{K} in THF at 300 K (black) and simulated spectrum in red (9.4169 GHz; simulation parameters: g_{iso} = 2.046, A_{iso}($^{185/187}$Re) = 770 MHz).

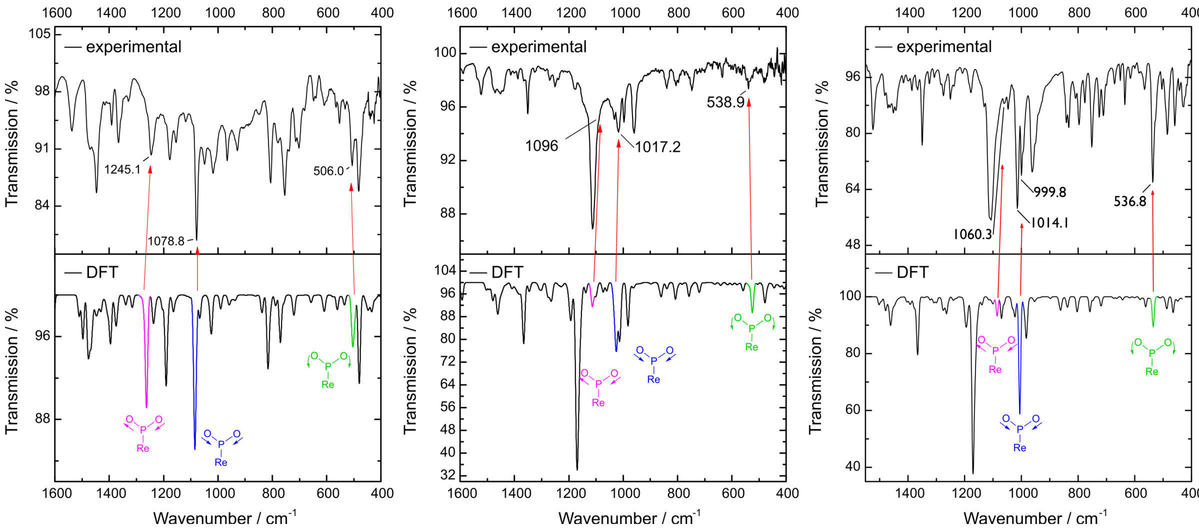

Figure S15 Comparison of experimental (*top*) and DFT calculated (RI-M06L/def2-SVP, *bottom*) IR spectra of complexes **2** (*left*), $\mathbf{2^K}$ (*middle*) and $\mathbf{3^K}$ (*right*) and assignments of the asymmetric (*magenta*) and symmetric (*blue*) stretching and symmetric bending (*green*) modes of the PO_2 ligands.

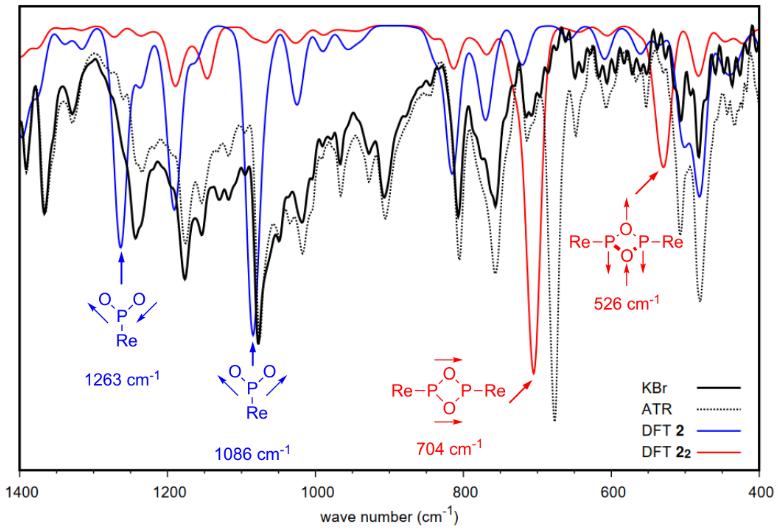

Figure S16 IR transmission spectra of a solid sample of **2**/$\mathbf{2_2}$ obtained from rapid evaporation of a benzene/THF solution (ATR, dotted line) and a sample of **2** as KBr pellet (black line) in comparison with DFT computed spectra for **2** (blue line) and $\mathbf{2_2}$ (red line).

Determination of the Diffusion Constant of 2

According to the Stokes-Einstein equation the hydrodynamic radius of the complex was estimated from the diffusion coefficient *D*:

$$r_0 = \frac{k_B * T}{6\pi * \eta * D}$$

$\eta_{THF,25\,°C}$ = 0.456 mPa·s;[3] $\eta_{Benzene,25\,°C}$ = 0.604 mP a·s.[4]

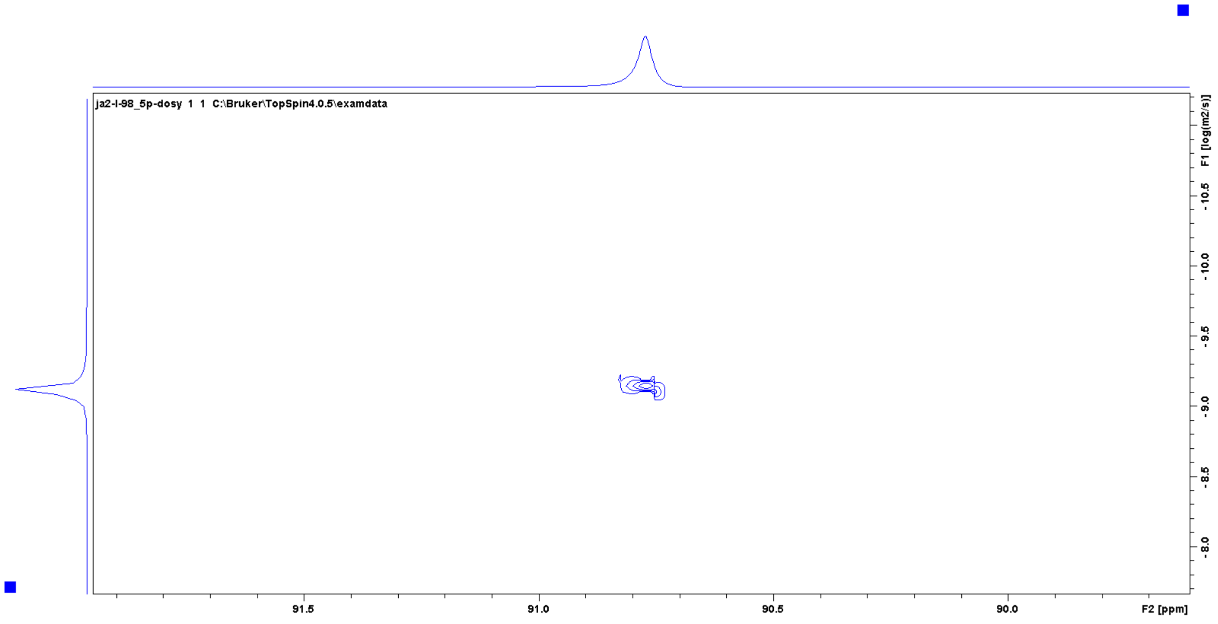

Figure S17. ^{31}P{^{1}H}-DOSY NMR spectrum of complex **1** in C_6D_6 at room temperature (*D* = 7.1 x 10^{-10} m²·s^{-1}, r_0 = 5.1 Å).

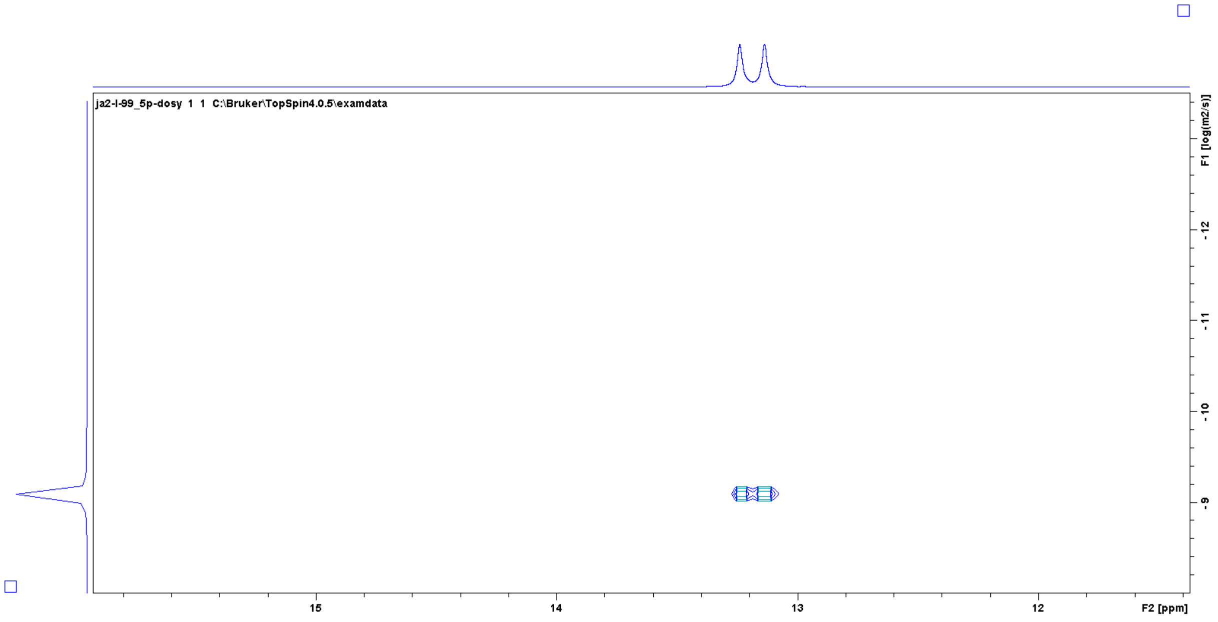

Figure S18. ^{31}P{^{1}H}-DOSY NMR spectrum of complex **2** in C_6D_6 at room temperature (*D* = 7.3 x 10^{-10} m²·s^{-1}, r_0 = 4.9 Å).

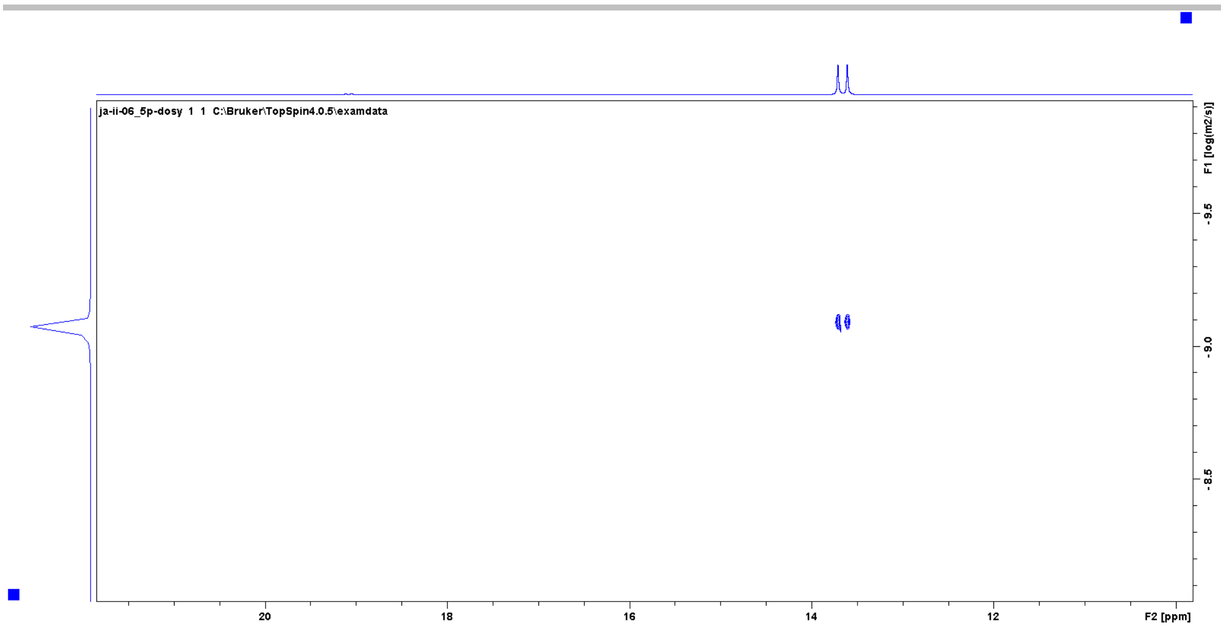

Figure S19. $^{31}P\{^{1}H\}$-DOSY NMR spectrum of complex **2** in THF-d_8 at room temperature (D = 8.1 x 10^{-10} $m^2 \cdot s^{-1}$, r_0 = 5.9 Å).

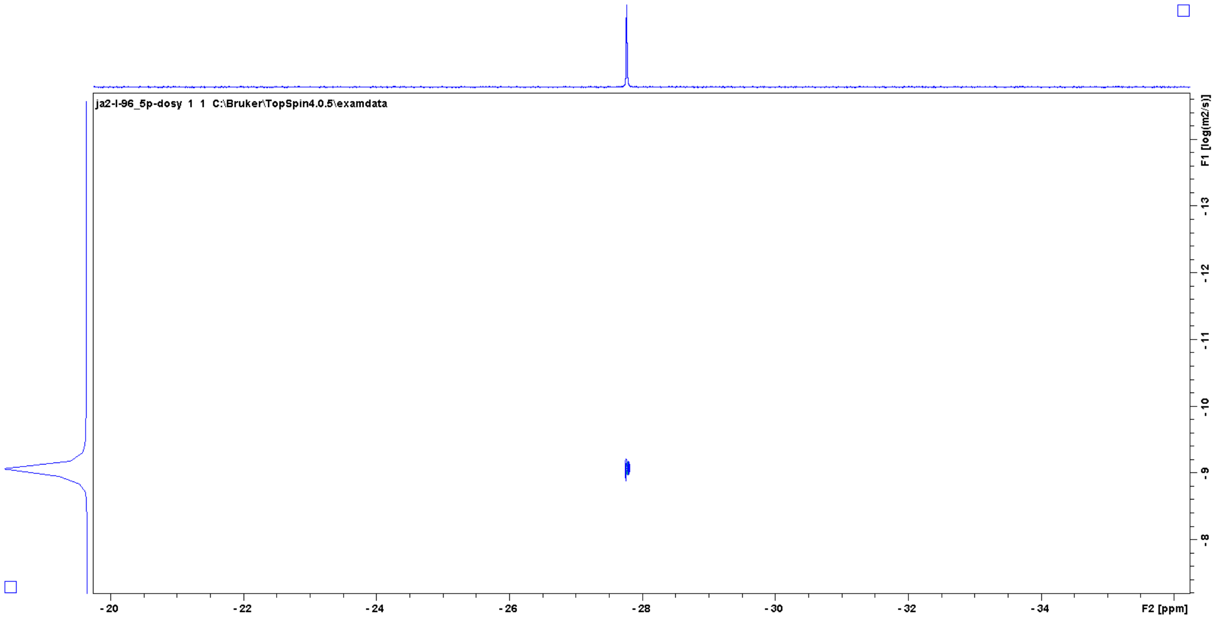

Figure S20. $^{31}P\{^{1}H\}$-DOSY NMR spectrum of [ReI(κ^2-N^1,N^3-PyrPz)(PNP)] in C_6D_6 at room temperature (D = 8.4 x 10^{-10} $m^2 \cdot s^{-1}$, r_0 = 4.3 Å).

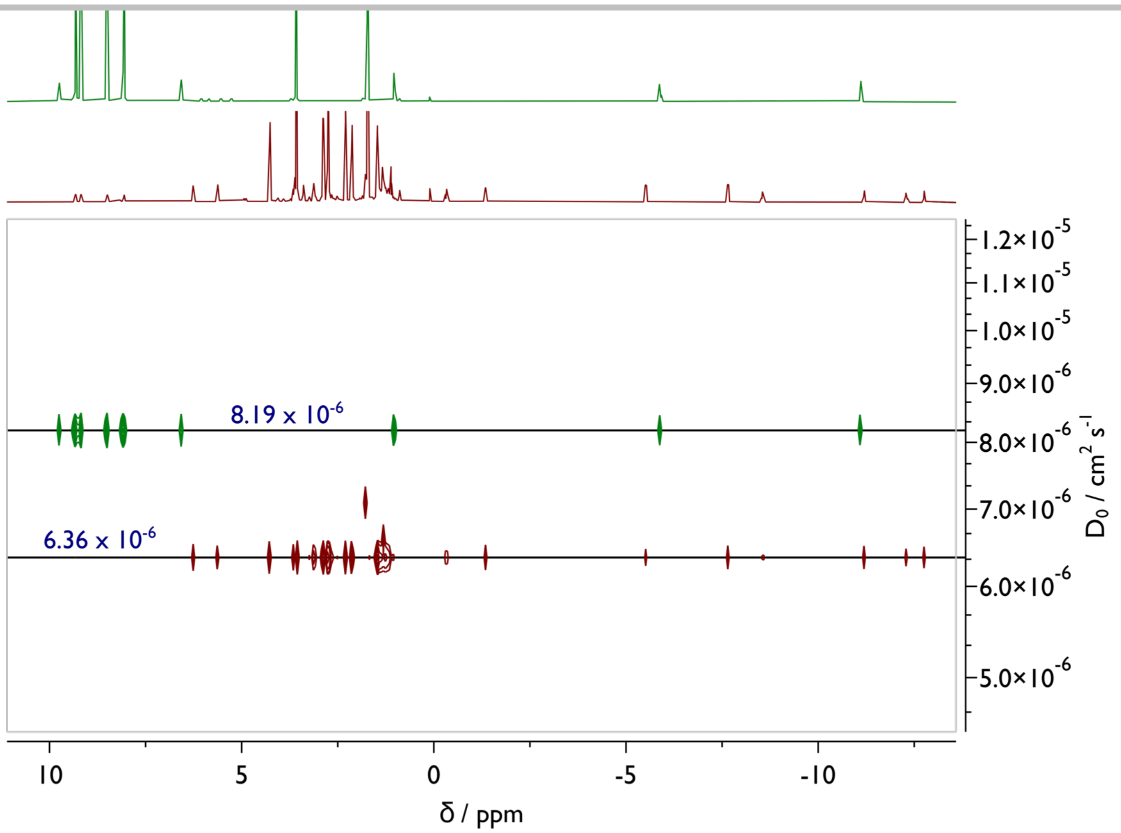

Figure S21. $^1H\{^{31}P\}$-DOSY NMR spectra of [ReCl$_3$(HPNP')] (*green*) and [(µ-N$_2$)(ReCl$_2$(HPNP'))$_2$] (*red*) in THF-d_8 at room temperature.

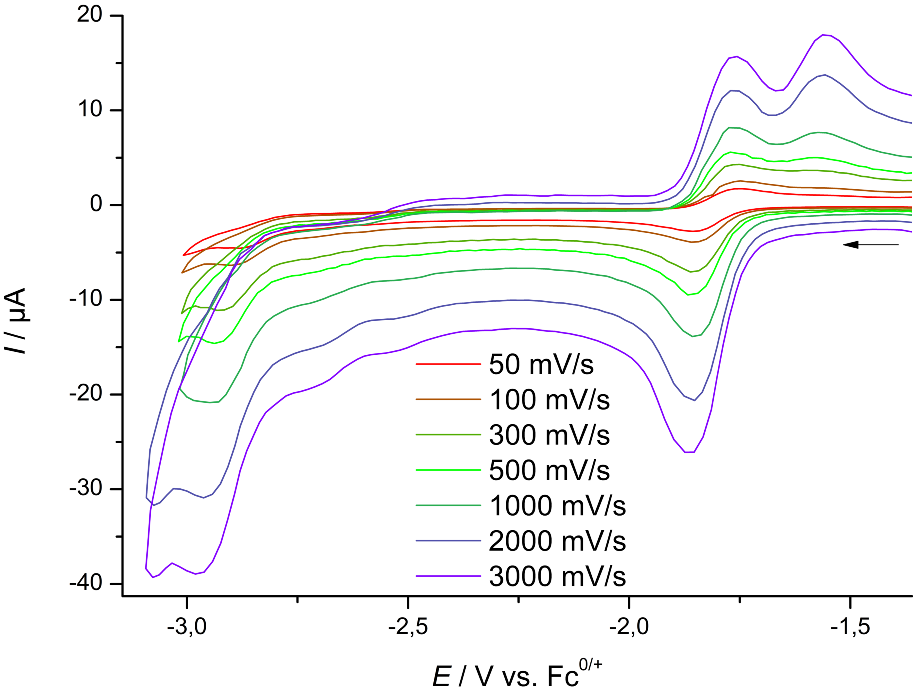

Figure S22. Cyclic voltammogram of complex **2** (1.0 mM, 0.2 M [NBu$_4$][PF$_6$], THF, room temperature).

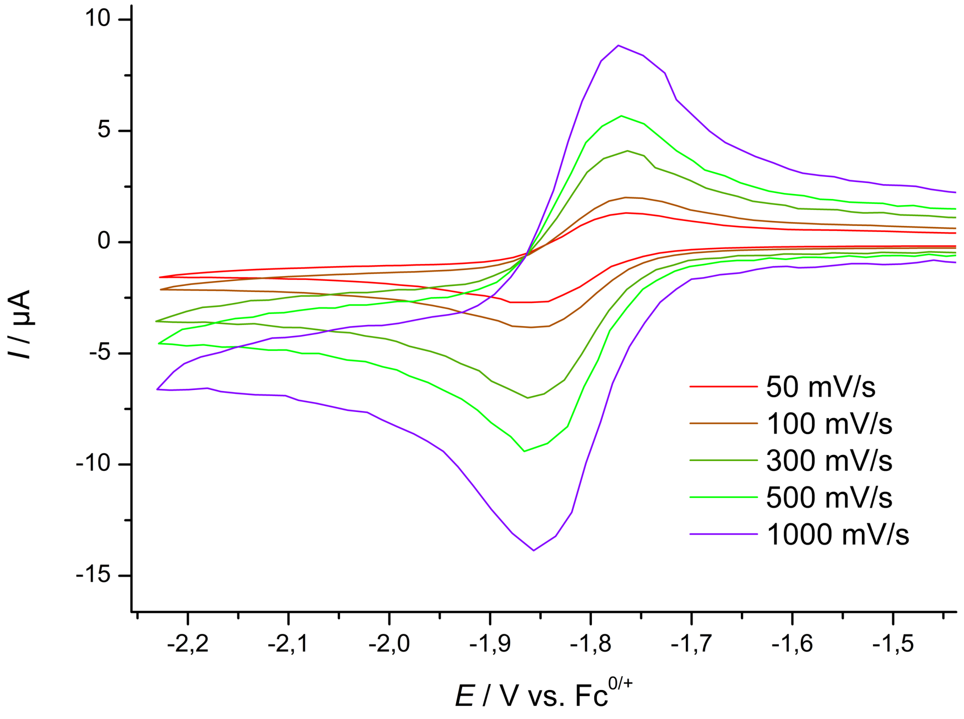

Figure S23. Cyclic voltammogram of complex **2** (first reductive event; 1.0 mM, 0.2 M $[NBu_4][PF_6]$, THF, room temperature).

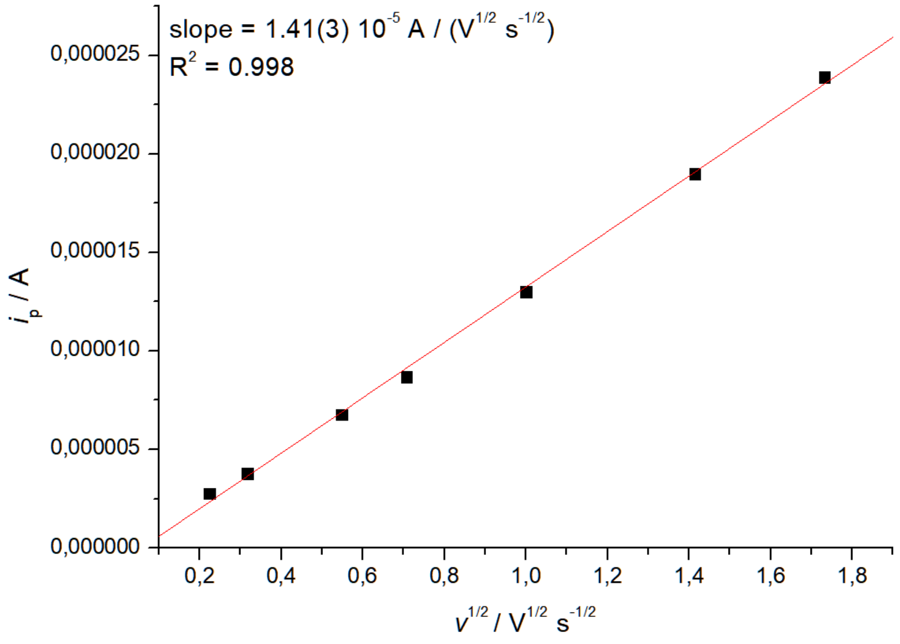

Figure S24. Randles-Sevcik-plot (i_p vs. $v^{1/2}$) with linear fit (red line) of the first reductive event of complex **2**.

The diffusion constant D_0 of complex 2 was derived from the Randles-Sevcik equation (at 25°C):

$$i_p = 2.69 \times 10^5\, n^{3/2}\, A\, D^{1/2}\, C\, v^{1/2}$$

With n as the number of transferred electrons (n = 1), A as the electrode surface (A = 0.02 cm^2), C as the concentration of **2** (C = 1 mM), D as the diffusion constant and v as the scan rate, D was calculated from the linear fit of the experimental data (m = 1.41· 10^{-5} $A \cdot s^{1/2} \cdot V^{-1/2}$):

$$i_p = m \cdot v^{1/2}$$

$$m = 2.69 \cdot 10^5 \cdot n^{3/2} \cdot A \cdot D^{1/2} \cdot C \cdot v^{1/2}$$

$$D = (m \cdot (2.69 \cdot 10^5 \cdot n^{3/2} \cdot A \cdot C)^{-1})^2$$

$$D = 6.9\ 10^{-10}\ m^2 \cdot s^{-1}.$$

Controlled potential electrolysis of 2

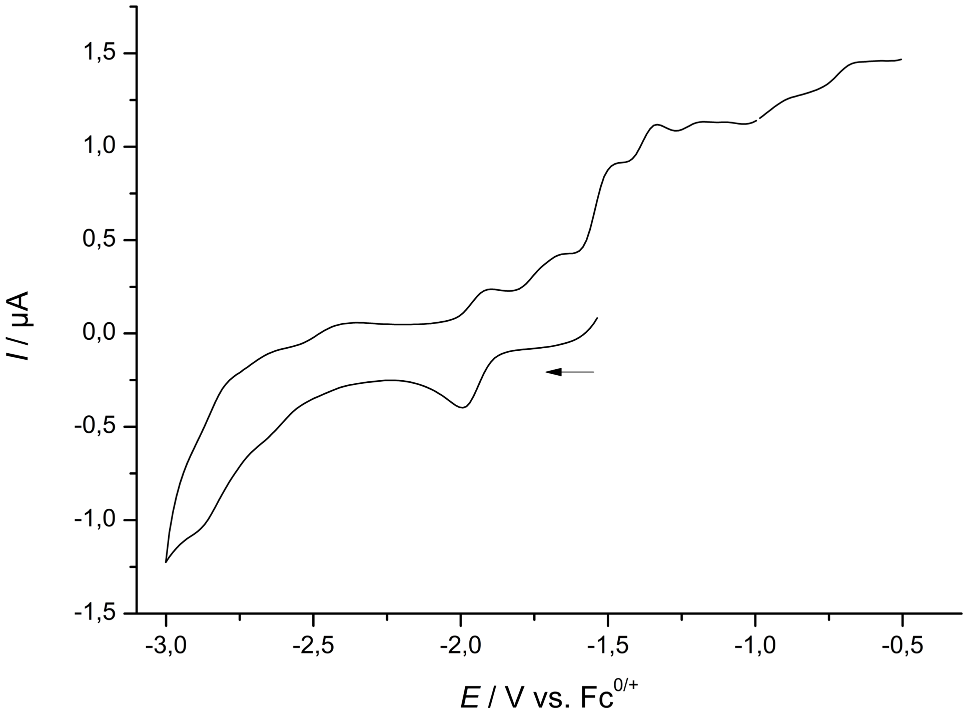

Figure S25. CV of complex **2** after electrolysis; initial concentration of **2** c_0 = 1.06 mM (0.1 v s^{-1}, 1 M $[NBu_4][PF_6]$, THF, room temperature).

A 1.06 mM solution of **2** (3 mL) was electrolyzed for 16 h in THF. A small residual amount of **2** was detected *via* cyclic voltammetry after electrolysis and calculated to correspond to a concentration of 0.12 µM.

The number of transferred electrons per Re was calculated by:

$$z = \frac{Q}{n * F}$$

Q: electric charge (C), F: Faraday constant (C mol^{-1}) , n: amount of substance (mol).

	Q / mC
$Q_{expected}$ after 16 h and 88 % conversion	276
$Q_{measured}$ after 16 h	236

Therefore, z equals 0.86 e⁻.

Crystallographic Details

Suitable single crystals for X-ray structure determination were selected from the mother liquor under an inert gas atmosphere and transferred in protective perfluoro polyether oil on a microscope slide. The selected and mounted crystals were transferred to the cold gas stream on the diffractometer. The diffraction data were obtained at 100 K on a Bruker D8 three-circle diffractometer, equipped with a PHOTON 100 CMOS detector and an INCOATEC microfocus source with Quazar mirror optics (Mo-Kα radiation, λ = 0.71073 Å).
The data obtained were integrated with SAINT and a semi-empirical absorption correction from equivalents with SADABS was applied. The structure was solved and refined using the Bruker SHELX 2014 software package.[5] All non-hydrogen atoms were refined with anisotropic displacement parameters. All C–H hydrogen atoms were refined isotropically on calculated positions by using a riding model with their U_{iso} values constrained to 1.5 U_{eq} of their pivot atoms for terminal sp^3 carbon atoms and 1.2 times for all other atoms.

X-ray Single-Crystal Structure Analysis of 2_2

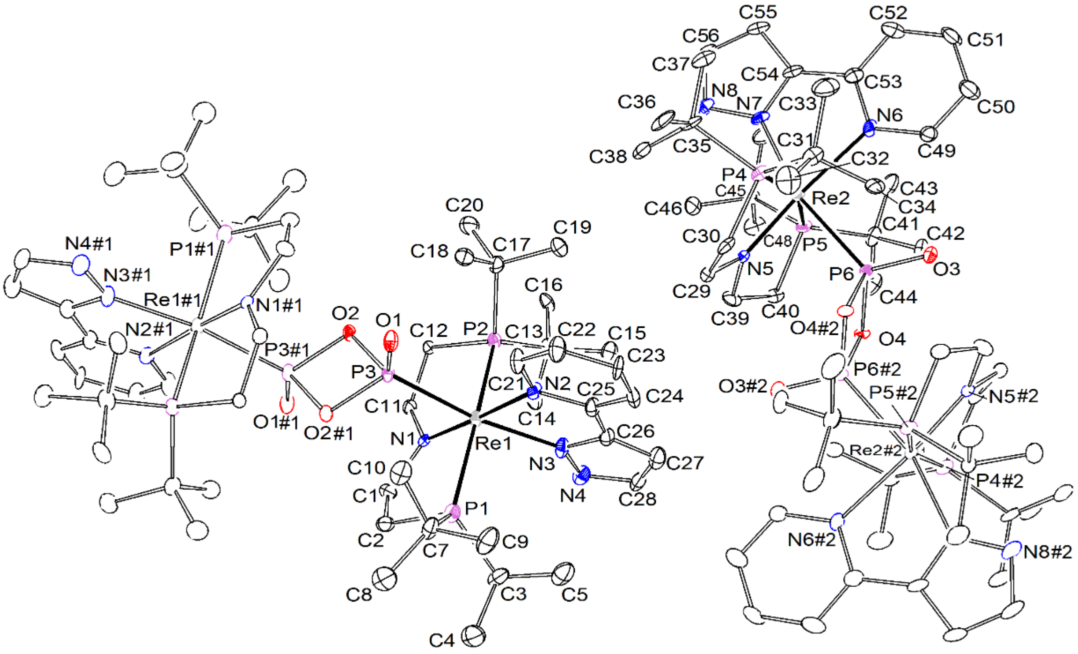

Figure S26. Thermal ellipsoid plot of 2_2 with the anisotropic displacement parameters drawn at the 50% probability level. The asymmetric unit contains two half complex molecules and was refined using some restraints and constraints (RIGU, EADP). The SQUEEZE algorithm[6] was used to omit three heavily disordered Et_2O solvent molecules.

Table S1. Crystal data and structure refinement for 2_2.

CCDC	2024567	
Empirical formula	$C_{56}H_{100}N_8O_4P_6Re_2$ (+ 3 Et_2O solvent molecules)	
Formula weight	1507.65	
Temperature	101(2) K	
Wavelength	0.71073 Å	
Crystal system	Triclinic	
Space group	P-1	
Unit cell dimensions	a = 15.3409(15) Å	α = 113.168(2)°
	b = 15.4611(14) Å	β = 91.184(3)°
	c = 17.6625(15) Å	γ = 103.670(3)°
Volume	3712.0(6) $Å^3$	
Z	2	
Density (calculated)	1.349 Mg/m^3	
Absorption coefficient	3.430 mm^{-1}	
F(000)	1528	
Crystal size	0.296 x 0.165 x 0.150 mm^3	

Crystal shape and color	Block, clear intense green/brown
Theta range for data collection	2.193 to 25.407°
Index ranges	-18<=h<=18, -18<=k<=18, -20<=l<=21
Reflections collected	97135
Independent reflections	13625 [R(int) = 0.1572]
Completeness to theta = 25.242°	99.9 %
Refinement method	Full-matrix least-squares on F^2
Data / restraints / parameters	13625 / 732 / 697
Goodness-of-fit on F^2	1.026
Final R indices [I>2sigma(I)]	R1 = 0.0432, wR2 = 0.0907
R indices (all data)	R1 = 0.0816, wR2 = 0.1031
Largest diff. peak and hole	1.852 and -1.154 eÅ^{-3}

Table S2. Bond lengths [Å] and angles [°] for **2_2**.

Re(1)-N(1)	1.900(5)	C(1)-C(2)	1.537(10)
Re(1)-N(3)	2.143(6)	C(3)-C(6)	1.522(10)
Re(1)-N(2)	2.263(5)	C(3)-C(4)	1.541(10)
Re(1)-P(1)	2.4457(19)	C(3)-C(5)	1.545(11)
Re(1)-P(2)	2.4528(18)	C(7)-C(10)	1.525(10)
Re(1)-P(3)	2.4564(18)	C(7)-C(8)	1.531(10)
Re(2)-N(5)	1.900(5)	C(7)-C(9)	1.540(10)
Re(2)-N(7)	2.143(5)	C(11)-C(12)	1.522(9)
Re(2)-N(6)	2.268(6)	C(13)-C(14)	1.531(9)
Re(2)-P(4)	2.4437(19)	C(13)-C(15)	1.537(9)
Re(2)-P(5)	2.4521(18)	C(13)-C(16)	1.539(9)
Re(2)-P(6)	2.4556(17)	C(17)-C(20)	1.517(10)
P(1)-C(2)	1.824(7)	C(17)-C(18)	1.527(10)
P(1)-C(7)	1.913(8)	C(17)-C(19)	1.535(9)
P(1)-C(3)	1.918(7)	C(21)-C(22)	1.385(10)
P(2)-C(12)	1.839(6)	C(22)-C(23)	1.394(9)
P(2)-C(13)	1.908(7)	C(23)-C(24)	1.357(10)
P(2)-C(17)	1.911(7)	C(24)-C(25)	1.385(9)
P(3)-O(1)	1.484(5)	C(25)-C(26)	1.441(10)
P(3)-O(2)	1.685(5)	C(26)-C(27)	1.386(10)
P(3)-O(2)#1	1.685(5)	C(27)-C(28)	1.374(11)
P(3)-P(3)#1	2.528(3)	C(29)-C(30)	1.523(9)
P(4)-C(30)	1.840(7)	C(31)-C(34)	1.524(9)
P(4)-C(31)	1.910(7)	C(31)-C(32)	1.530(10)
P(4)-C(35)	1.911(7)	C(31)-C(33)	1.547(10)
P(5)-C(40)	1.819(7)	C(35)-C(36)	1.540(10)
P(5)-C(41)	1.899(7)	C(35)-C(37)	1.541(10)
P(5)-C(45)	1.906(7)	C(35)-C(38)	1.543(10)
P(6)-O(3)	1.485(5)	C(39)-C(40)	1.517(10)
P(6)-O(4)	1.671(4)	C(41)-C(43)	1.518(10)
P(6)-O(4)#2	1.679(5)	C(41)-C(42)	1.526(9)
P(6)-P(6)#2	2.515(3)	C(41)-C(44)	1.539(10)
O(2)-P(3)#1	1.685(5)	C(45)-C(48)	1.535(9)
O(4)-P(6)#2	1.679(5)	C(45)-C(46)	1.536(10)
N(1)-C(1)	1.490(8)	C(45)-C(47)	1.545(9)
N(1)-C(11)	1.496(8)	C(49)-C(50)	1.379(10)
N(2)-C(21)	1.362(9)	C(50)-C(51)	1.389(10)
N(2)-C(25)	1.365(8)	C(51)-C(52)	1.376(10)
N(3)-N(4)	1.347(8)	C(52)-C(53)	1.390(10)
N(3)-C(26)	1.365(9)	C(53)-C(54)	1.445(10)
N(4)-C(28)	1.352(9)	C(54)-C(55)	1.397(9)
N(5)-C(29)	1.486(8)	C(55)-C(56)	1.366(10)
N(5)-C(39)	1.500(8)		
N(6)-C(53)	1.360(8)	N(1)-Re(1)-N(3)	111.4(2)
N(6)-C(49)	1.363(8)	N(1)-Re(1)-N(2)	175.5(2)
N(7)-C(54)	1.336(9)	N(3)-Re(1)-N(2)	72.8(2)
N(7)-N(8)	1.354(8)	N(1)-Re(1)-P(1)	83.30(17)
N(8)-C(56)	1.347(9)	N(3)-Re(1)-P(1)	89.35(16)

N(2)-Re(1)-P(1)	95.39(15)	O(4)-P(6)-Re(2)	117.04(17)
N(1)-Re(1)-P(2)	82.78(17)	O(4)#2-P(6)-Re(2)	117.26(17)
N(3)-Re(1)-P(2)	88.10(16)	O(3)-P(6)-P(6)#2	114.7(2)
N(2)-Re(1)-P(2)	99.08(15)	O(4)-P(6)-P(6)#2	41.46(16)
P(1)-Re(1)-P(2)	163.84(6)	O(4)#2-P(6)-P(6)#2	41.22(15)
N(1)-Re(1)-P(3)	82.75(16)	Re(2)-P(6)-P(6)#2	127.43(11)
N(3)-Re(1)-P(3)	165.84(16)	P(3)-O(2)-P(3)#1	97.2(2)
N(2)-Re(1)-P(3)	93.10(15)	P(6)-O(4)-P(6)#2	97.3(2)
P(1)-Re(1)-P(3)	93.48(6)	C(1)-N(1)-C(11)	107.9(5)
P(2)-Re(1)-P(3)	92.88(6)	C(1)-N(1)-Re(1)	125.9(4)
N(5)-Re(2)-N(7)	113.1(2)	C(11)-N(1)-Re(1)	126.0(4)
N(5)-Re(2)-N(6)	174.7(2)	C(21)-N(2)-C(25)	116.5(6)
N(7)-Re(2)-N(6)	72.1(2)	C(21)-N(2)-Re(1)	127.0(4)
N(5)-Re(2)-P(4)	82.42(17)	C(25)-N(2)-Re(1)	116.4(5)
N(7)-Re(2)-P(4)	88.82(16)	N(4)-N(3)-C(26)	110.2(6)
N(6)-Re(2)-P(4)	97.59(15)	N(4)-N(3)-Re(1)	130.1(4)
N(5)-Re(2)-P(5)	82.76(17)	C(26)-N(3)-Re(1)	119.7(5)
N(7)-Re(2)-P(5)	88.48(16)	N(3)-N(4)-C(28)	105.4(6)
N(6)-Re(2)-P(5)	98.03(15)	C(29)-N(5)-C(39)	107.2(5)
P(4)-Re(2)-P(5)	162.49(6)	C(29)-N(5)-Re(2)	126.7(4)
N(5)-Re(2)-P(6)	81.89(16)	C(39)-N(5)-Re(2)	125.8(4)
N(7)-Re(2)-P(6)	165.00(16)	C(53)-N(6)-C(49)	115.8(6)
N(6)-Re(2)-P(6)	92.85(14)	C(53)-N(6)-Re(2)	116.6(4)
P(4)-Re(2)-P(6)	93.30(6)	C(49)-N(6)-Re(2)	127.6(4)
P(5)-Re(2)-P(6)	93.78(6)	C(54)-N(7)-N(8)	109.9(5)
C(2)-P(1)-C(7)	104.9(3)	C(54)-N(7)-Re(2)	120.5(5)
C(2)-P(1)-C(3)	101.4(3)	N(8)-N(7)-Re(2)	129.6(4)
C(7)-P(1)-C(3)	106.3(3)	C(56)-N(8)-N(7)	105.1(6)
C(2)-P(1)-Re(1)	97.6(2)	N(1)-C(1)-C(2)	113.8(6)
C(7)-P(1)-Re(1)	123.6(2)	C(1)-C(2)-P(1)	111.3(5)
C(3)-P(1)-Re(1)	118.9(2)	C(6)-C(3)-C(4)	106.0(6)
C(12)-P(2)-C(13)	100.5(3)	C(6)-C(3)-C(5)	109.6(6)
C(12)-P(2)-C(17)	105.1(3)	C(4)-C(3)-C(5)	107.9(6)
C(13)-P(2)-C(17)	106.7(3)	C(6)-C(3)-P(1)	106.8(5)
C(12)-P(2)-Re(1)	97.2(2)	C(4)-C(3)-P(1)	113.7(5)
C(13)-P(2)-Re(1)	120.7(2)	C(5)-C(3)-P(1)	112.6(5)
C(17)-P(2)-Re(1)	122.3(2)	C(10)-C(7)-C(8)	107.0(6)
O(1)-P(3)-O(2)	107.4(3)	C(10)-C(7)-C(9)	106.4(6)
O(1)-P(3)-O(2)#1	108.3(3)	C(8)-C(7)-C(9)	110.6(6)
O(2)-P(3)-O(2)#1	82.8(2)	C(10)-C(7)-P(1)	109.0(5)
O(1)-P(3)-Re(1)	119.2(2)	C(8)-C(7)-P(1)	113.5(5)
O(2)-P(3)-Re(1)	116.99(18)	C(9)-C(7)-P(1)	109.9(5)
O(2)#1-P(3)-Re(1)	116.20(17)	N(1)-C(11)-C(12)	113.7(5)
O(1)-P(3)-P(3)#1	114.1(2)	C(11)-C(12)-P(2)	110.5(4)
O(2)-P(3)-P(3)#1	41.41(16)	C(14)-C(13)-C(15)	109.6(6)
O(2)#1-P(3)-P(3)#1	41.39(16)	C(14)-C(13)-C(16)	105.6(6)
Re(1)-P(3)-P(3)#1	126.64(11)	C(15)-C(13)-C(16)	106.9(6)
C(30)-P(4)-C(31)	104.9(3)	C(14)-C(13)-P(2)	107.7(5)
C(30)-P(4)-C(35)	100.5(3)	C(15)-C(13)-P(2)	112.4(5)
C(31)-P(4)-C(35)	106.3(3)	C(16)-C(13)-P(2)	114.4(5)
C(30)-P(4)-Re(2)	98.1(2)	C(20)-C(17)-C(18)	107.4(6)
C(31)-P(4)-Re(2)	122.9(2)	C(20)-C(17)-C(19)	110.7(6)
C(35)-P(4)-Re(2)	119.9(2)	C(18)-C(17)-C(19)	105.8(6)
C(40)-P(5)-C(41)	105.2(3)	C(20)-C(17)-P(2)	113.9(5)
C(40)-P(5)-C(45)	101.0(3)	C(18)-C(17)-P(2)	109.4(5)
C(41)-P(5)-C(45)	106.5(3)	C(19)-C(17)-P(2)	109.4(5)
C(40)-P(5)-Re(2)	97.4(2)	N(2)-C(21)-C(22)	122.6(7)
C(41)-P(5)-Re(2)	122.9(2)	C(21)-C(22)-C(23)	120.4(7)
C(45)-P(5)-Re(2)	119.7(2)	C(24)-C(23)-C(22)	116.7(7)
O(3)-P(6)-O(4)	108.5(2)	C(23)-C(24)-C(25)	121.9(7)
O(3)-P(6)-O(4)#2	108.0(3)	N(2)-C(25)-C(24)	121.9(7)
O(4)-P(6)-O(4)#2	82.7(2)	N(2)-C(25)-C(26)	114.5(6)
O(3)-P(6)-Re(2)	117.9(2)	C(24)-C(25)-C(26)	123.5(7)

N(3)-C(26)-C(27)	108.1(7)	C(42)-C(41)-P(5)	109.4(5)
N(3)-C(26)-C(25)	116.4(6)	C(44)-C(41)-P(5)	113.7(5)
C(27)-C(26)-C(25)	135.4(7)	C(48)-C(45)-C(46)	106.5(6)
C(28)-C(27)-C(26)	104.1(7)	C(48)-C(45)-C(47)	106.5(6)
N(4)-C(28)-C(27)	112.2(7)	C(46)-C(45)-C(47)	109.7(6)
N(5)-C(29)-C(30)	114.2(6)	C(48)-C(45)-P(5)	114.4(5)
C(29)-C(30)-P(4)	110.3(5)	C(46)-C(45)-P(5)	106.7(5)
C(34)-C(31)-C(32)	106.9(6)	C(47)-C(45)-P(5)	112.8(5)
C(34)-C(31)-C(33)	106.5(6)	N(6)-C(49)-C(50)	123.5(7)
C(32)-C(31)-C(33)	111.2(6)	C(49)-C(50)-C(51)	119.9(7)
C(34)-C(31)-P(4)	109.0(5)	C(52)-C(51)-C(50)	117.5(7)
C(32)-C(31)-P(4)	113.4(5)	C(51)-C(52)-C(53)	120.2(7)
C(33)-C(31)-P(4)	109.5(5)	N(6)-C(53)-C(52)	123.1(7)
C(36)-C(35)-C(37)	107.1(6)	N(6)-C(53)-C(54)	114.0(6)
C(36)-C(35)-C(38)	105.7(6)	C(52)-C(53)-C(54)	123.0(7)
C(37)-C(35)-C(38)	109.8(6)	N(7)-C(54)-C(55)	109.4(6)
C(36)-C(35)-P(4)	114.1(5)	N(7)-C(54)-C(53)	116.8(6)
C(37)-C(35)-P(4)	113.3(5)	C(55)-C(54)-C(53)	133.8(7)
C(38)-C(35)-P(4)	106.7(5)	C(56)-C(55)-C(54)	102.8(6)
N(5)-C(39)-C(40)	113.7(5)	N(8)-C(56)-C(55)	112.8(6)
C(39)-C(40)-P(5)	111.5(5)		
C(43)-C(41)-C(42)	107.7(6)		
C(43)-C(41)-C(44)	109.5(6)		
C(42)-C(41)-C(44)	106.2(6)		
C(43)-C(41)-P(5)	110.1(5)		

Symmetry transformations used to generate equivalent atoms:
#1 -x+2,-y+1,-z   #2 -x+1,-y+1,-z+1

Table S3. Torsion angles [°] for **2**.

O(1)-P(3)-O(2)-P(3)#1	-107.0(3)	C(21)-C(22)-C(23)-C(24)	-0.6(11)
O(2)#1-P(3)-O(2)-P(3)#1	0.000(2)	C(22)-C(23)-C(24)-C(25)	1.2(11)
Re(1)-P(3)-O(2)-P(3)#1	115.79(19)	C(21)-N(2)-C(25)-C(24)	3.1(10)
O(3)-P(6)-O(4)-P(6)#2	106.6(3)	Re(1)-N(2)-C(25)-C(24)	-174.0(5)
O(4)#2-P(6)-O(4)-P(6)#2	0.000(1)	C(21)-N(2)-C(25)-C(26)	-179.5(6)
Re(2)-P(6)-O(4)-P(6)#2	-116.93(19)	Re(1)-N(2)-C(25)-C(26)	3.5(8)
C(26)-N(3)-N(4)-C(28)	0.3(8)	C(23)-C(24)-C(25)-N(2)	-2.6(11)
Re(1)-N(3)-N(4)-C(28)	178.1(5)	C(23)-C(24)-C(25)-C(26)	-179.8(7)
N(7)-Re(2)-N(5)-C(29)	-92.8(5)	N(4)-N(3)-C(26)-C(27)	0.3(8)
P(4)-Re(2)-N(5)-C(29)	-7.3(5)	Re(1)-N(3)-C(26)-C(27)	-177.8(5)
P(5)-Re(2)-N(5)-C(29)	-178.0(5)	N(4)-N(3)-C(26)-C(25)	-178.8(6)
P(6)-Re(2)-N(5)-C(29)	87.1(5)	Re(1)-N(3)-C(26)-C(25)	3.1(8)
N(7)-Re(2)-N(5)-C(39)	93.7(5)	N(2)-C(25)-C(26)-N(3)	-4.3(9)
P(4)-Re(2)-N(5)-C(39)	179.1(5)	C(24)-C(25)-C(26)-N(3)	173.1(7)
P(5)-Re(2)-N(5)-C(39)	8.5(5)	N(2)-C(25)-C(26)-C(27)	176.9(8)
P(6)-Re(2)-N(5)-C(39)	-86.4(5)	C(24)-C(25)-C(26)-C(27)	-5.7(13)
C(54)-N(7)-N(8)-C(56)	-0.2(8)	N(3)-C(26)-C(27)-C(28)	-0.8(8)
Re(2)-N(7)-N(8)-C(56)	-179.6(5)	C(25)-C(26)-C(27)-C(28)	178.1(8)
C(11)-N(1)-C(1)-C(2)	-164.2(6)	N(3)-N(4)-C(28)-C(27)	-0.8(8)
Re(1)-N(1)-C(1)-C(2)	10.7(8)	C(26)-C(27)-C(28)-N(4)	1.0(8)
N(1)-C(1)-C(2)-P(1)	-28.0(7)	C(39)-N(5)-C(29)-C(30)	163.6(5)
C(7)-P(1)-C(2)-C(1)	156.3(5)	Re(2)-N(5)-C(29)-C(30)	-10.9(8)
C(3)-P(1)-C(2)-C(1)	-93.2(5)	N(5)-C(29)-C(30)-P(4)	28.2(7)
Re(1)-P(1)-C(2)-C(1)	28.4(5)	C(31)-P(4)-C(30)-C(29)	-156.1(5)
C(1)-N(1)-C(11)-C(12)	162.3(6)	C(35)-P(4)-C(30)-C(29)	93.7(5)
Re(1)-N(1)-C(11)-C(12)	-12.5(8)	Re(2)-P(4)-C(30)-C(29)	-28.8(5)
N(1)-C(11)-C(12)-P(2)	31.0(7)	C(29)-N(5)-C(39)-C(40)	-164.7(6)
C(13)-P(2)-C(12)-C(11)	92.0(5)	Re(2)-N(5)-C(39)-C(40)	9.9(8)
C(17)-P(2)-C(12)-C(11)	-157.4(5)	N(5)-C(39)-C(40)-P(5)	-28.8(7)
Re(1)-P(2)-C(12)-C(11)	-31.2(5)	C(41)-P(5)-C(40)-C(39)	157.0(5)
C(25)-N(2)-C(21)-C(22)	-2.5(11)	C(45)-P(5)-C(40)-C(39)	-92.3(5)
Re(1)-N(2)-C(21)-C(22)	174.2(6)	Re(2)-P(5)-C(40)-C(39)	30.0(5)
N(2)-C(21)-C(22)-C(23)	1.3(12)	C(40)-P(5)-C(41)-C(43)	157.3(5)

C(45)-P(5)-C(41)-C(43)	50.7(6)
Re(2)-P(5)-C(41)-C(43)	-93.2(5)
C(40)-P(5)-C(41)-C(42)	-84.6(5)
C(45)-P(5)-C(41)-C(42)	168.8(5)
Re(2)-P(5)-C(41)-C(42)	25.0(6)
C(40)-P(5)-C(41)-C(44)	33.9(6)
C(45)-P(5)-C(41)-C(44)	-72.7(6)
Re(2)-P(5)-C(41)-C(44)	143.4(4)
C(53)-N(6)-C(49)-C(50)	-0.4(10)
Re(2)-N(6)-C(49)-C(50)	-179.4(6)
N(6)-C(49)-C(50)-C(51)	0.6(12)
C(49)-C(50)-C(51)-C(52)	-0.9(12)
C(50)-C(51)-C(52)-C(53)	1.1(12)
C(49)-N(6)-C(53)-C(52)	0.6(10)
Re(2)-N(6)-C(53)-C(52)	179.7(6)
C(49)-N(6)-C(53)-C(54)	-179.6(6)
Re(2)-N(6)-C(53)-C(54)	-0.5(8)
C(51)-C(52)-C(53)-N(6)	-1.0(12)
C(51)-C(52)-C(53)-C(54)	179.2(7)
N(8)-N(7)-C(54)-C(55)	0.5(8)
Re(2)-N(7)-C(54)-C(55)	179.9(4)
N(8)-N(7)-C(54)-C(53)	179.0(6)
Re(2)-N(7)-C(54)-C(53)	-1.6(9)
N(6)-C(53)-C(54)-N(7)	1.3(9)
C(52)-C(53)-C(54)-N(7)	-178.9(7)
N(6)-C(53)-C(54)-C(55)	179.4(7)
C(52)-C(53)-C(54)-C(55)	-0.8(13)
N(7)-C(54)-C(55)-C(56)	-0.5(8)
C(53)-C(54)-C(55)-C(56)	-178.7(8)
N(7)-N(8)-C(56)-C(55)	-0.2(8)
C(54)-C(55)-C(56)-N(8)	0.5(8)

Symmetry transformations used to generate equivalent atoms:
#1 -x+2,-y+1,-z   #2 -x+1,-y+1,-z+1

X-ray Single-Crystal Structure Analysis of 3^K

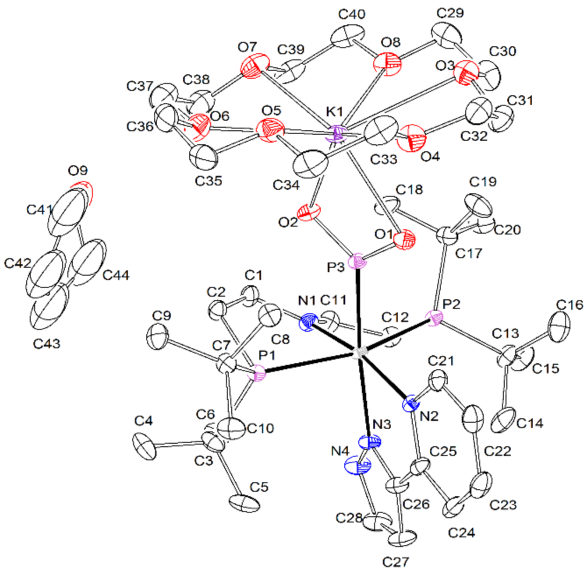

Figure S27. Thermal ellipsoid plot of 3^K with the anisotropic displacement parameters drawn at the 50% probability level. The asymmetric unit contains one complex molecule and a disordered THF molecule. The disordered THF molecule was set to a population of 0.5 and refined using some restraints and constraints (SADI, EADP).

Table S4. Crystal data and structure refinement for 3^K.

CCDC	2024610	
Empirical formula	$C_{40}H_{73}KN_4O_8P_3Re$ x 0.5 (C_4H_8O)	
Formula weight	1092.28	
Temperature	100(2) K	
Wavelength	0.71073 Å	
Crystal system	Monoclinic	
Space group	$P2_1/c$	
Unit cell dimensions	a = 12.8339(4) Å	α = 90°.
	b = 23.5174(8) Å	β = 100.086(2)°.
	c = 16.6688(5) Å	γ = 90°.
Volume	4953.2(3) $Å^3$	
Z	4	
Density (calculated)	1.465 Mg/m^3	
Absorption coefficient	2.686 mm^{-1}	

F(000)	2256
Crystal size	0.454 x 0.162 x 0.100 mm^3
Crystal shape and color	Plate, clear intense purple
Theta range for data collection	2.364 to 28.369°.
Index ranges	-17<=h<=17, -31<=k<=31, -22<=l<=22
Reflections collected	215272
Independent reflections	12383 [R(int) = 0.0992]
Completeness to theta = 25.242°	99.9 %
Refinement method	Full-matrix least-squares on F^2
Data / restraints / parameters	12383 / 4 / 558
Goodness-of-fit on F^2	1.048
Final R indices [I>2sigma(I)]	R1 = 0.0291, wR2 = 0.0519.
R indices (all data)	R1 = 0.0454, wR2 = 0.0566.
Largest diff. peak and hole	1.743 and -0.934 $eÅ^{-3}$

Table S5. Bond lengths [Å] and angles [°] for **3^{K}**.

Re(1)-N(1)	2.082(2)	C(3)-C(5)	1.538(4)
Re(1)-N(2)	2.141(2)	C(3)-C(6)	1.540(4)
Re(1)-N(3)	2.162(2)	C(7)-C(10)	1.534(4)
Re(1)-P(3)	2.2545(7)	C(7)-C(8)	1.536(4)
Re(1)-P(1)	2.3977(7)	C(7)-C(9)	1.540(4)
Re(1)-P(2)	2.4332(7)	C(11)-C(12)	1.486(4)
K(1)-O(1)	2.730(2)	C(13)-C(14)	1.525(4)
K(1)-O(2)	2.731(2)	C(13)-C(15)	1.533(4)
K(1)-O(8)	2.806(2)	C(13)-C(16)	1.541(5)
K(1)-O(4)	2.866(2)	C(15)-K(1)#2	3.257(3)
K(1)-O(6)	2.893(2)	C(15)-H(15A)	1.02(4)
K(1)-O(3)	2.949(2)	C(15)-H(15B)	0.98(4)
K(1)-O(7)	2.979(2)	C(15)-H(15C)	1.00(4)
K(1)-O(5)	2.980(2)	C(17)-C(19)	1.526(4)
K(1)-C(15)#1	3.257(3)	C(17)-C(20)	1.540(4)
K(1)-P(3)	3.2830(9)	C(17)-C(18)	1.547(4)
K(1)-C(31)	3.490(3)	C(21)-C(22)	1.380(4)
P(1)-C(2)	1.844(3)	C(22)-C(23)	1.385(5)
P(1)-C(7)	1.919(3)	C(23)-C(24)	1.366(4)
P(1)-C(3)	1.921(3)	C(24)-C(25)	1.398(4)
P(2)-C(12)	1.855(3)	C(25)-C(26)	1.428(4)
P(2)-C(13)	1.905(3)	C(26)-C(27)	1.400(4)
P(2)-C(17)	1.910(3)	C(27)-C(28)	1.381(5)
P(3)-O(2)	1.512(2)	C(29)-C(30)	1.494(5)
P(3)-O(1)	1.517(2)	C(31)-C(32)	1.495(4)
O(3)-C(30)	1.419(4)	C(33)-C(34)	1.497(5)
O(3)-C(31)	1.422(4)	C(35)-C(36)	1.486(5)
O(4)-C(33)	1.411(4)	C(37)-C(38)	1.491(5)
O(4)-C(32)	1.424(4)	C(39)-C(40)	1.489(5)
O(5)-C(34)	1.416(4)	C(41)-C(42)	1.464(13)
O(5)-C(35)	1.418(4)	C(41)-O(9)	1.661(16)
O(6)-C(37)	1.411(4)	O(9)-C(44)	1.433(14)
O(6)-C(36)	1.420(4)	C(42)-C(43)	1.555(14)
O(7)-C(39)	1.421(4)	C(43)-C(44)	1.410(14)
O(7)-C(38)	1.423(4)		
O(8)-C(29)	1.411(4)	N(1)-Re(1)-N(2)	168.83(9)
O(8)-C(40)	1.419(4)	N(1)-Re(1)-N(3)	94.31(9)
N(1)-C(11)	1.327(4)	N(2)-Re(1)-N(3)	74.85(9)
N(1)-C(1)	1.468(4)	N(1)-Re(1)-P(3)	94.69(7)
N(2)-C(21)	1.362(4)	N(2)-Re(1)-P(3)	96.17(6)
N(2)-C(25)	1.381(3)	N(3)-Re(1)-P(3)	171.00(6)
N(3)-N(4)	1.354(3)	N(1)-Re(1)-P(1)	78.51(7)
N(3)-C(26)	1.358(4)	N(2)-Re(1)-P(1)	99.06(6)
N(4)-C(28)	1.351(4)	N(3)-Re(1)-P(1)	92.97(7)
C(1)-C(2)	1.526(4)	P(3)-Re(1)-P(1)	88.98(2)
C(3)-C(4)	1.532(4)	N(1)-Re(1)-P(2)	77.19(7)

N(2)-Re(1)-P(2)	104.79(6)	C(7)-P(1)-Re(1)	123.85(9)
N(3)-Re(1)-P(2)	88.86(6)	C(3)-P(1)-Re(1)	120.21(10)
P(3)-Re(1)-P(2)	92.97(2)	C(12)-P(2)-C(13)	102.69(13)
P(1)-Re(1)-P(2)	155.70(2)	C(12)-P(2)-C(17)	101.81(13)
O(1)-K(1)-O(2)	54.14(6)	C(13)-P(2)-C(17)	106.14(14)
O(1)-K(1)-O(8)	91.93(7)	C(12)-P(2)-Re(1)	96.03(10)
O(2)-K(1)-O(8)	81.27(6)	C(13)-P(2)-Re(1)	124.55(10)
O(1)-K(1)-O(4)	86.42(6)	C(17)-P(2)-Re(1)	120.35(9)
O(2)-K(1)-O(4)	138.01(6)	O(2)-P(3)-O(1)	110.26(12)
O(8)-K(1)-O(4)	117.68(7)	O(2)-P(3)-Re(1)	124.53(9)
O(1)-K(1)-O(6)	125.71(7)	O(1)-P(3)-Re(1)	125.18(8)
O(2)-K(1)-O(6)	84.26(7)	O(2)-P(3)-K(1)	55.63(8)
O(8)-K(1)-O(6)	116.92(7)	O(1)-P(3)-K(1)	55.62(8)
O(4)-K(1)-O(6)	113.68(7)	Re(1)-P(3)-K(1)	169.26(3)
O(1)-K(1)-O(3)	84.17(6)	P(3)-O(1)-K(1)	97.09(10)
O(2)-K(1)-O(3)	121.35(6)	P(3)-O(2)-K(1)	97.18(10)
O(8)-K(1)-O(3)	58.64(6)	C(30)-O(3)-C(31)	111.1(2)
O(4)-K(1)-O(3)	59.23(6)	C(30)-O(3)-K(1)	104.87(18)
O(6)-K(1)-O(3)	149.92(7)	C(31)-O(3)-K(1)	100.01(18)
O(1)-K(1)-O(7)	122.15(6)	C(33)-O(4)-C(32)	112.2(2)
O(2)-K(1)-O(7)	71.59(6)	C(33)-O(4)-K(1)	122.51(19)
O(8)-K(1)-O(7)	58.90(7)	C(32)-O(4)-K(1)	116.84(17)
O(4)-K(1)-O(7)	150.34(6)	C(34)-O(5)-C(35)	111.6(3)
O(6)-K(1)-O(7)	58.24(7)	C(34)-O(5)-K(1)	111.42(18)
O(3)-K(1)-O(7)	111.77(7)	C(35)-O(5)-K(1)	111.59(18)
O(1)-K(1)-O(5)	119.01(6)	C(37)-O(6)-C(36)	112.2(3)
O(2)-K(1)-O(5)	126.65(7)	C(37)-O(6)-K(1)	117.9(2)
O(8)-K(1)-O(5)	146.29(6)	C(36)-O(6)-K(1)	120.01(19)
O(4)-K(1)-O(5)	56.70(6)	C(39)-O(7)-C(38)	111.3(3)
O(6)-K(1)-O(5)	56.99(7)	C(39)-O(7)-K(1)	103.07(17)
O(3)-K(1)-O(5)	108.40(6)	C(38)-O(7)-K(1)	103.36(18)
O(7)-K(1)-O(5)	108.05(6)	C(29)-O(8)-C(40)	113.8(3)
O(1)-K(1)-C(15)#1	156.45(9)	C(29)-O(8)-K(1)	120.47(18)
O(2)-K(1)-C(15)#1	138.73(8)	C(40)-O(8)-K(1)	120.3(2)
O(8)-K(1)-C(15)#1	74.51(8)	C(11)-N(1)-C(1)	115.0(2)
O(4)-K(1)-C(15)#1	83.21(8)	C(11)-N(1)-Re(1)	122.9(2)
O(6)-K(1)-C(15)#1	77.84(9)	C(1)-N(1)-Re(1)	121.88(18)
O(3)-K(1)-C(15)#1	72.35(9)	C(21)-N(2)-C(25)	115.9(2)
O(7)-K(1)-C(15)#1	67.33(8)	C(21)-N(2)-Re(1)	126.86(18)
O(5)-K(1)-C(15)#1	71.82(8)	C(25)-N(2)-Re(1)	117.22(17)
O(1)-K(1)-P(3)	27.30(4)	N(4)-N(3)-C(26)	109.5(2)
O(2)-K(1)-P(3)	27.20(4)	N(4)-N(3)-Re(1)	134.15(18)
O(8)-K(1)-P(3)	89.32(5)	C(26)-N(3)-Re(1)	116.24(18)
O(4)-K(1)-P(3)	111.57(5)	C(28)-N(4)-N(3)	105.9(2)
O(6)-K(1)-P(3)	103.71(5)	N(1)-C(1)-C(2)	114.1(2)
O(3)-K(1)-P(3)	105.90(5)	C(1)-C(2)-P(1)	111.42(19)
O(7)-K(1)-P(3)	98.02(5)	C(4)-C(3)-C(5)	108.8(3)
O(5)-K(1)-P(3)	124.26(5)	C(4)-C(3)-C(6)	106.6(3)
C(15)#1-K(1)-P(3)	162.15(7)	C(5)-C(3)-C(6)	107.5(2)
O(1)-K(1)-C(31)	70.06(7)	C(4)-C(3)-P(1)	115.3(2)
O(2)-K(1)-C(31)	119.58(7)	C(5)-C(3)-P(1)	111.1(2)
O(8)-K(1)-C(31)	79.18(8)	C(6)-C(3)-P(1)	107.2(2)
O(4)-K(1)-C(31)	42.14(7)	C(10)-C(7)-C(8)	106.2(2)
O(6)-K(1)-C(31)	154.13(7)	C(10)-C(7)-C(9)	110.5(2)
O(3)-K(1)-C(31)	23.67(7)	C(8)-C(7)-C(9)	105.9(2)
O(7)-K(1)-C(31)	135.27(8)	C(10)-C(7)-P(1)	110.26(19)
O(5)-K(1)-C(31)	98.09(7)	C(8)-C(7)-P(1)	109.46(19)
C(15)#1-K(1)-C(31)	88.26(9)	C(9)-C(7)-P(1)	114.2(2)
P(3)-K(1)-C(31)	96.20(6)	N(1)-C(11)-C(12)	118.9(3)
C(2)-P(1)-C(7)	102.49(13)	C(11)-C(12)-P(2)	107.32(19)
C(2)-P(1)-C(3)	100.43(14)	C(14)-C(13)-C(15)	107.5(3)
C(7)-P(1)-C(3)	105.57(13)	C(14)-C(13)-C(16)	106.3(3)
C(2)-P(1)-Re(1)	99.48(9)	C(15)-C(13)-C(16)	109.6(3)

C(14)-C(13)-P(2)	107.7(2)
C(15)-C(13)-P(2)	114.8(2)
C(16)-C(13)-P(2)	110.5(2)
C(13)-C(15)-K(1)#2	160.4(2)
C(13)-C(15)-H(15A)	107(2)
K(1)#2-C(15)-H(15A)	86(2)
C(13)-C(15)-H(15B)	106(2)
K(1)#2-C(15)-H(15B)	55(2)
H(15A)-C(15)-H(15B)	111(3)
C(13)-C(15)-H(15C)	114(2)
K(1)#2-C(15)-H(15C)	74(2)
H(15A)-C(15)-H(15C)	112(3)
H(15B)-C(15)-H(15C)	108(3)
C(19)-C(17)-C(20)	108.7(3)
C(19)-C(17)-C(18)	108.1(3)
C(20)-C(17)-C(18)	105.0(2)
C(19)-C(17)-P(2)	111.4(2)
C(20)-C(17)-P(2)	114.6(2)
C(18)-C(17)-P(2)	108.8(2)
N(2)-C(21)-C(22)	123.2(3)
C(21)-C(22)-C(23)	120.4(3)
C(24)-C(23)-C(22)	117.8(3)
C(23)-C(24)-C(25)	120.6(3)
N(2)-C(25)-C(24)	122.1(3)
N(2)-C(25)-C(26)	114.6(2)
C(24)-C(25)-C(26)	123.2(3)
N(3)-C(26)-C(27)	109.1(3)
N(3)-C(26)-C(25)	116.8(2)
C(27)-C(26)-C(25)	133.8(3)
C(28)-C(27)-C(26)	103.1(3)
N(4)-C(28)-C(27)	112.4(3)
O(8)-C(29)-C(30)	107.5(3)
O(3)-C(30)-C(29)	109.2(3)
O(3)-C(31)-C(32)	108.7(3)
O(3)-C(31)-K(1)	56.32(15)
C(32)-C(31)-K(1)	87.30(19)
O(4)-C(32)-C(31)	107.7(3)
O(4)-C(33)-C(34)	108.3(3)
O(5)-C(34)-C(33)	109.7(3)
O(5)-C(35)-C(36)	108.9(3)
O(6)-C(36)-C(35)	109.1(3)
O(6)-C(37)-C(38)	109.7(3)
O(7)-C(38)-C(37)	108.7(3)
O(7)-C(39)-C(40)	110.2(3)
O(8)-C(40)-C(39)	108.5(3)
C(42)-C(41)-O(9)	86.8(11)
C(44)-O(9)-C(41)	106.2(12)
C(41)-C(42)-C(43)	126.3(14)
C(44)-C(43)-C(42)	91.4(13)
C(43)-C(44)-O(9)	111.4(13)

Symmetry transformations used to generate equivalent atoms:
#1 -x+1,y-1/2,-z+3/2 #2 -x+1,y+1/2,-z+3/2

Table S6. Torsion angles [°] for **3**^{K}.

O(2)-P(3)-O(1)-K(1)	10.95(12)
Re(1)-P(3)-O(1)-K(1)	-166.95(4)
O(1)-P(3)-O(2)-K(1)	-10.95(13)
Re(1)-P(3)-O(2)-K(1)	166.97(4)
C(26)-N(3)-N(4)-C(28)	-0.3(3)
Re(1)-N(3)-N(4)-C(28)	176.4(2)
C(11)-N(1)-C(1)-C(2)	165.4(3)
Re(1)-N(1)-C(1)-C(2)	-19.4(3)
N(1)-C(1)-C(2)-P(1)	-12.9(3)
C(7)-P(1)-C(2)-C(1)	159.8(2)
C(3)-P(1)-C(2)-C(1)	-91.6(2)
Re(1)-P(1)-C(2)-C(1)	31.8(2)
C(1)-N(1)-C(11)-C(12)	169.1(3)
Re(1)-N(1)-C(11)-C(12)	-6.1(4)
N(1)-C(11)-C(12)-P(2)	-27.4(3)
C(13)-P(2)-C(12)-C(11)	167.1(2)
C(17)-P(2)-C(12)-C(11)	-83.1(2)
Re(1)-P(2)-C(12)-C(11)	39.6(2)
C(14)-C(13)-C(15)-K(1)#2	-69.3(8)
C(16)-C(13)-C(15)-K(1)#2	45.9(8)
P(2)-C(13)-C(15)-K(1)#2	170.9(6)
C(25)-N(2)-C(21)-C(22)	-1.1(4)
Re(1)-N(2)-C(21)-C(22)	178.8(2)
N(2)-C(21)-C(22)-C(23)	0.1(5)
C(21)-C(22)-C(23)-C(24)	0.6(5)
C(22)-C(23)-C(24)-C(25)	-0.1(4)
C(21)-N(2)-C(25)-C(24)	1.6(4)
Re(1)-N(2)-C(25)-C(24)	-178.4(2)
C(21)-N(2)-C(25)-C(26)	-174.9(2)
Re(1)-N(2)-C(25)-C(26)	5.2(3)
C(23)-C(24)-C(25)-N(2)	-1.0(4)
C(23)-C(24)-C(25)-C(26)	175.1(3)
N(4)-N(3)-C(26)-C(27)	0.0(3)
Re(1)-N(3)-C(26)-C(27)	-177.4(2)
N(4)-N(3)-C(26)-C(25)	174.2(3)
Re(1)-N(3)-C(26)-C(25)	-3.2(3)
N(2)-C(25)-C(26)-N(3)	-1.3(4)
C(24)-C(25)-C(26)-N(3)	-177.7(3)
N(2)-C(25)-C(26)-C(27)	171.2(3)
C(24)-C(25)-C(26)-C(27)	-5.2(5)
N(3)-C(26)-C(27)-C(28)	0.3(4)
C(25)-C(26)-C(27)-C(28)	-172.5(3)
N(3)-N(4)-C(28)-C(27)	0.5(4)
C(26)-C(27)-C(28)-N(4)	-0.5(4)
C(40)-O(8)-C(29)-C(30)	-173.3(3)
K(1)-O(8)-C(29)-C(30)	32.6(3)
C(31)-O(3)-C(30)-C(29)	172.6(3)
K(1)-O(3)-C(30)-C(29)	65.4(3)
O(8)-C(29)-C(30)-O(3)	-68.1(3)
C(30)-O(3)-C(31)-C(32)	176.2(3)
K(1)-O(3)-C(31)-C(32)	-73.4(3)
C(30)-O(3)-C(31)-K(1)	-110.3(2)
C(33)-O(4)-C(32)-C(31)	-176.5(3)
K(1)-O(4)-C(32)-C(31)	-27.5(3)
O(3)-C(31)-C(32)-O(4)	72.8(3)
K(1)-C(31)-C(32)-O(4)	19.8(2)
C(32)-O(4)-C(33)-C(34)	-177.5(3)
K(1)-O(4)-C(33)-C(34)	35.5(3)
C(35)-O(5)-C(34)-C(33)	-176.9(3)
K(1)-O(5)-C(34)-C(33)	57.6(3)

O(4)-C(33)-C(34)-O(5)	-61.7(3)
C(34)-O(5)-C(35)-C(36)	176.6(3)
K(1)-O(5)-C(35)-C(36)	-58.0(3)
C(37)-O(6)-C(36)-C(35)	176.2(3)
K(1)-O(6)-C(36)-C(35)	-38.8(3)
O(5)-C(35)-C(36)-O(6)	64.6(3)
C(36)-O(6)-C(37)-C(38)	174.0(3)
K(1)-O(6)-C(37)-C(38)	28.2(4)
C(39)-O(7)-C(38)-C(37)	179.1(3)
K(1)-O(7)-C(38)-C(37)	69.1(3)
O(6)-C(37)-C(38)-O(7)	-69.1(4)
C(38)-O(7)-C(39)-C(40)	-176.4(3)
K(1)-O(7)-C(39)-C(40)	-66.2(3)
C(29)-O(8)-C(40)-C(39)	177.2(3)
K(1)-O(8)-C(40)-C(39)	-28.6(3)
O(7)-C(39)-C(40)-O(8)	67.3(3)
C(42)-C(41)-O(9)-C(44)	-31.4(13)
O(9)-C(41)-C(42)-C(43)	13(3)
C(41)-C(42)-C(43)-C(44)	11(3)
C(42)-C(43)-C(44)-O(9)	-34(2)
C(41)-O(9)-C(44)-C(43)	47.6(17)

Symmetry transformations used to generate equivalent atoms:
#1 -x+1,y-1/2,-z+3/2   #2 -x+1,y+1/2,-z+3/2

Table S7. Hydrogen bonds for **3^{K}** [Å and °].

D-H...A	d(D-H)	d(H...A)	d(D...A)	<(DHA)
C(15)-H(15B)...K(1)#2	0.98(4)	2.81(4)	3.257(3)	108(2)

Symmetry transformations used to generate equivalent atoms:
#1 -x+1,y-1/2,-z+3/2   #2 -x+1,y+1/2,-z+3/2

DFT Calculations

Computational Details

Structure optimizations and single point calculations including electronic structure analyses were performed within the ORCA 4.2.1 program suite.[7] The thermodynamics of the dimerization equilibrium (2 **2** ⇌ $\mathbf{2_2}$) were evaluated by three methods employing different DFT functionals:

The molecular structure was either optimized applying the PBE functional[8] with the RI*J* approximation[9] to minimize computational costs and Ahlrichs' revised def2-SVP basis sets in combination with the corresponding auxiliary basis sets, which include an all electron basis for all elements but Re, for which a Stuttgart-Dresden 60 electron core potential replaces the inner shell 1s-4f orbitals.[10] To increase numerical accuracy and facilitate optimization of the molecular structure of $\mathbf{2_2}$, tight convergence criteria in the SCF procedure and a fine integration grid (Grid6 and GridX6) were applied. Additionally, single point calculations were conducted with the same method but without RI*J* or RIJCOSX approximation, using Ahlrichs' def2-TZVP basis for all atoms, again replacing the 60 core electrons of Re with the SD(60,MWB) effective core potential. The influence of the solvent (THF) was accounted for by Truhlar's SMD solvation model.[11] Thermodynamic corrections were evaluated by means of analytical vibrational analyses at the same level of theory as the geometry optimization applying Grimme's quasi-RRHO approach which treats low energy frequencies below 35 cm^{-1} as free rotors instead of harmonic vibrations for the vibrational partition function.[12]

In a second approach, the PBE0[13] functional was employed in both the optimizations and single point calculations. As PBE0 includes 25 % Hartree-Fock exchange, the RIJCOSX[14] integral evaluation routine was used instead of RI*J*. Otherwise the procedure was identical with method 1.

In a third method, Truhlar's strongly parametrized M06L density functional[15] was used for the structure optimizations to evaluate the influence of Grimme's dispersion correction, applying the RI*J* approximation and Ahlrich's def2-SVP basis. The thermodynamics are slightly biased by two low negative vibrational modes of $\mathbf{2_2}$ which could not be eliminated, even in repeated optimizations. Finally, single point energies were calculated with the M06 functional,[16] Ahlrichs' def2-TZVP basis set and the SMD solvation model.

The free energies were corrected for the difference between ideal gas standard conditions (1 atm, 298.15 K) and standard solution conditions (1 mol/L, 298.15 K), effectively reducing the calculated ΔG value of the equilibrium by 1.89 kcal·mol^{-1}:

$$G_{sol} = G_{gas} + RTln\frac{RT}{p}$$
$$G_{sol} = G_{gas} + RTln(24.47)$$
$$G_{sol} = G_{gas} + 1.89 \text{ kcal/mol}$$

NBO analyses were conducted using the D3BJ-RIJCOSX-PBE0/def2-TZVP single point calculations with the NBO 6.0 software of Landis and Weinhold.[17] In order to ensure comparability between **2**, $\mathbf{2}^{-}$ and $\mathbf{2}^{\mathbf{NO2}}$, all analyzed structures exhibit the same backbone conformation. In case of **2**, the lowest energy conformation deviates but NBO analyses of both conformers are very similar. The NBO Lewis structures of the PO_2/NO_2 groups were enforced with the CHOOSE command (see figure). The strength of π-backdonation was examined by investigating the interaction of the filled Re lone pairs and the P=O or N=O π^*-orbital by second order perturbation theory.

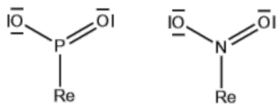

The structures obtained from method 2 were used to calculate the ^{31}P NMR shifts of **2** and $\mathbf{2_2}$, using the PBE0 functional, the RIJCOSX approximation and the GIAOs (Gauge Including Atomic Orbitals) approach.[18] As in the single point calculations, the def2-TZVP basis sets in combination with the def/2 auxiliary basis were employed except for the phosphorous and oxygen atoms, for which Jensen's special pcSseg-4 basis sets were utilized which were specifically developed for nuclear magnetic shielding calculations.[19] Very tight convergence criteria and a fine Grid (Grid6 and GridX6) were applied. The solvent (benzene) was included by means of the conductor-like polarizable continuum model (C-PCM)[20] as implemented in ORCA. As reference, the ^{31}P NMR shift of H_3PO_4 (0 ppm) calculated in water was used. The relative chemical shifts of the two ^{31}P signals computed for **2** ($\Delta\delta_P$) are in excellent agreement with experiment but differ from the absolute values by about 70 ppm, which partially arises from the missing inclusion of relativistic effects. Therefore, ^{31}P NMR chemical shift calculations were also performed with the NMR module[21] of the ADF program,[22] which accounts for relativistic spin-orbit contributions to nuclear magnetic shielding constants by means of the two-component ZORA formalism implemented in ADF.[23] The PBE0 hybrid functional was employed in combination with the TZ2P Slater type orbital basis set.[24] Solvent effects were taken into account with the COSMO model implemented in ADF.[25] The absolute chemical shifts obtained from this method are significantly closer (about 20 ppm) to the experimental results (see below).

Molecular graphics and vibrational spectra were rendered with the programs CylView,[26] Jmol[27] and ChemCraft.[28]

Computed geometries and electronic properties

Table S8. Calculated structural parameters (RI-M06L/def2-SVP; in Å and deg) of the PO_2 complexes in comparison to experiment (for atom captions see figures below).

	**2**	**2**$^{-}$	**2**^{K}	**2**$_{2}$	**2**$_{2}$ **(exp)**	**3**$^{-}$	**3**^{K}	**3**^{K} **(exp)**
Re-P1	2.459	2.442	2.446	2.470	2.4457(19)	2.404	2.422	2.3977(7)
Re-P2	2.463	2.436	2.453	2.472	2.4528(18)	2.441	2.457	2.4332(7)
Re-P3	2.408	2.319	2.266	2.458	2.4564(18)	2.300	2.248	2.2545(7)
Re-N1	1.916	2.052	2.045	1.912	1.900(5)	2.086	2.101	2.082(2)
Re-N2	2.313	2.195	2.200	2.333	2.263(5)	2.169	2.171	2.141(2)
Re-N3	2.140	2.192	2.178	2.155	2.143(6)	2.191	2.178	2.162(2)
P3-O1	1.508	1.528	1.545	1.516	1.484(5)	1.529	1.550	1.517(2)
P3-O2	1.511	1.524	1.542	1.714	1.685(5)	1.531	1.551	1.512(2)
O1-P3-O2	119.8	116.1	110.4			115.1	109.3	110.26(12)
O1,2-K			2.668 / 2.726				2.626 / 2.713	2.730 / 2.731

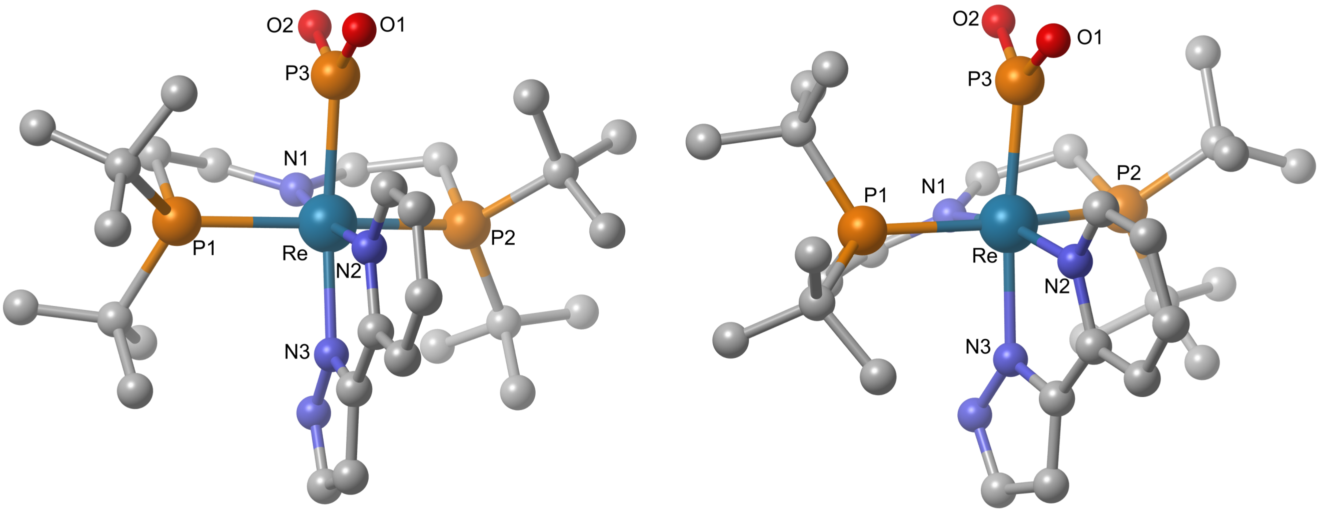

Figure S28. Optimized structures (RI-M06L/def2-SVP) of complexes **2** (left) and **2**$^{-}$ (right). Hydrogen atoms are omitted for clarity.

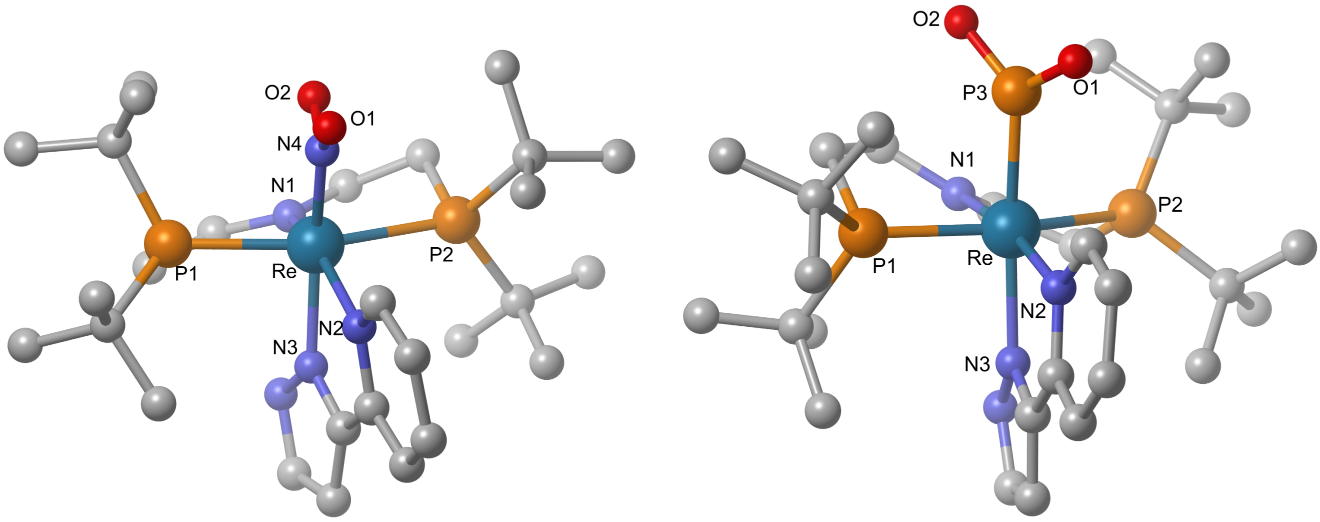

Figure S29. Optimized structures (RI-M06L/def2-SVP) of complexes **2**^{NO2} (left) and **3**$^{-}$ (right). Hydrogen atoms are omitted for clarity.

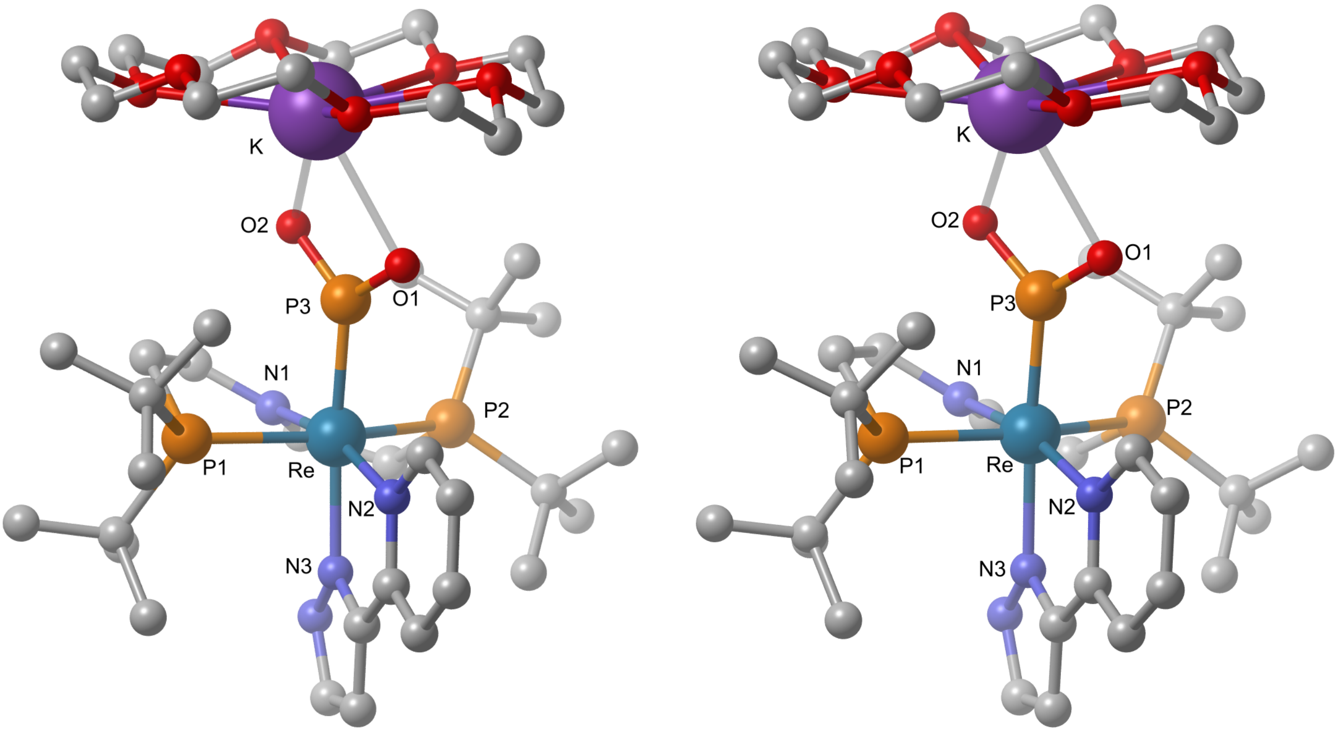

Figure S30. Optimized structures (RI-M06L/def2-SVP) of complexes **2**$^{\mathbf{K}}$ (left) and **3**$^{\mathbf{K}}$ (right). Hydrogen atoms are omitted for clarity.

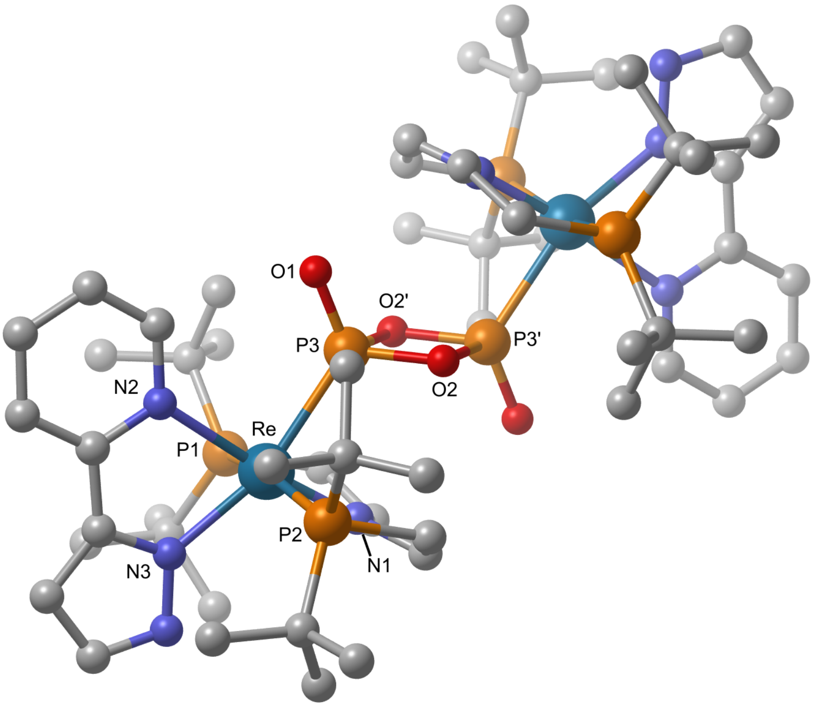

Figure S31. Optimized structures (RI-M06L/def2-SVP) of complex **2**$_{\mathbf{2}}$. Both Re fragments exhibit identical structural parameters. Hydrogen atoms are omitted for clarity.

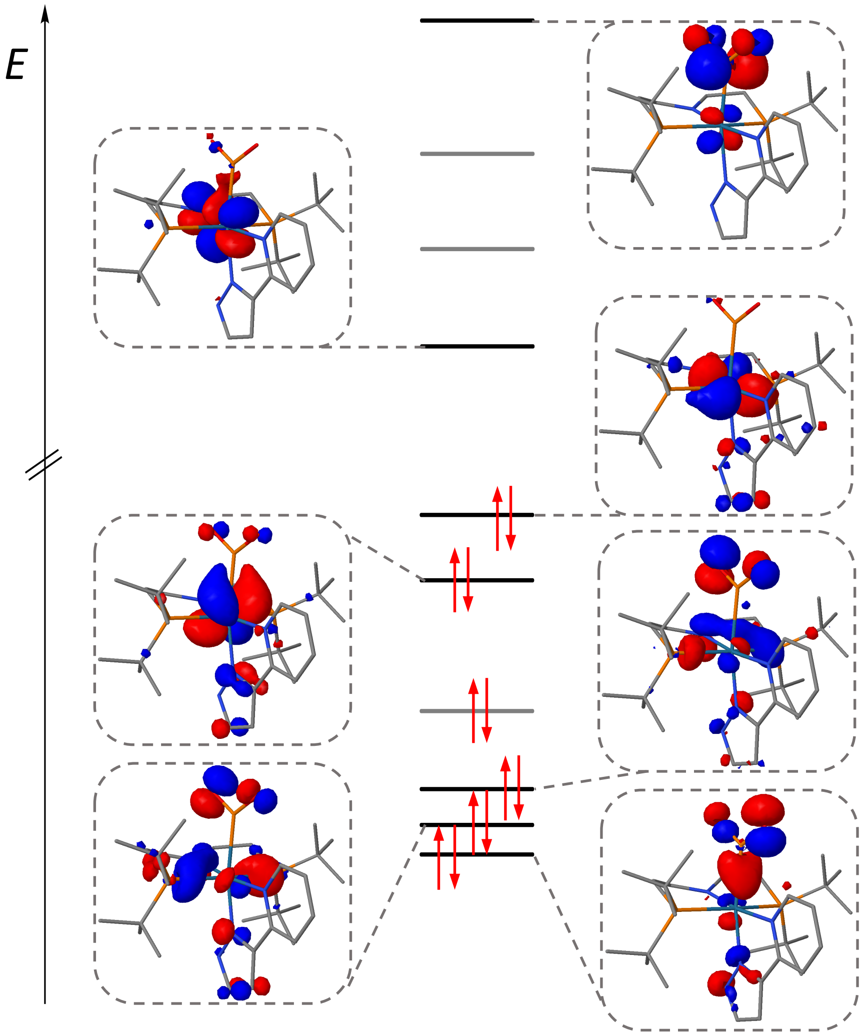

Figure S32. Molecular orbital scheme of complex **2** (D3BJ-PBE0/def2-TZVP). For simplicity, pure pyrazolpyridine ligand base π-type orbitals (in gray) are not included and H atoms are omitted.

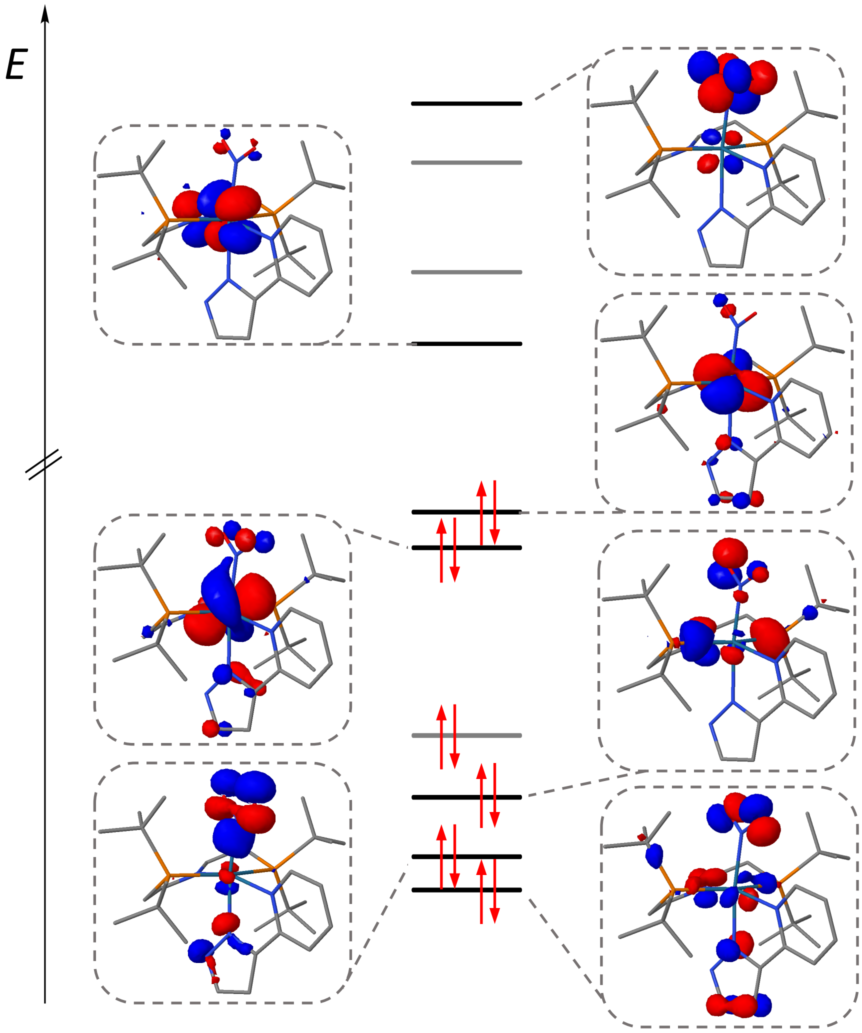

Figure S33. Molecular orbital scheme of complex **2**^{NO2} (D3BJ-PBE0/def2-TZVP). For simplicity, pure pyrazolpyridine ligand base π-type orbitals (in gray) are not included and H atoms are omitted.

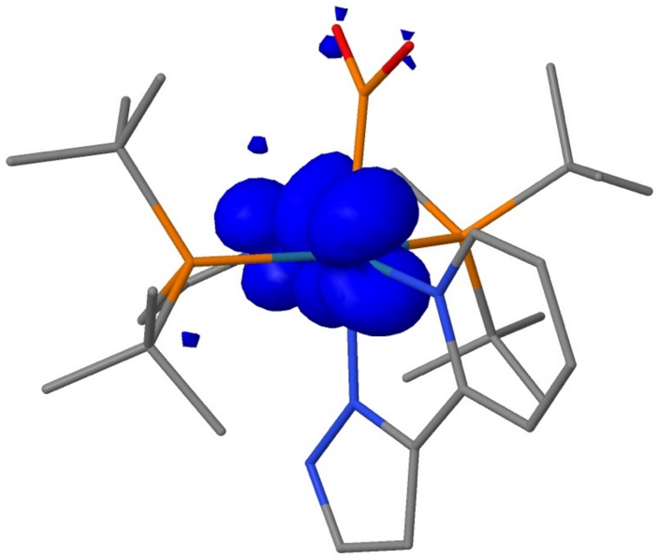

Figure S34. Spin density plot of **2**$^{-}$ (D3BJ-PBE0/def2-TZVP). For simplicity, H atoms are omitted.

Table S9. NLMO analyses of complexes **2**, **2**$^{-}$ and **2**^{NO2}.

**2**	**2**$^{-}$	**2**^{NO2}
Re–P bond<br>Wiberg Bond Index: 0.754<br>NLMO: 36 % Re, 61 % P<br>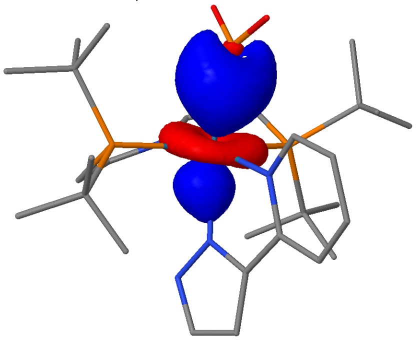	Re–P bond<br>Wiberg Bond Index: 0.817<br>NLMO[a]: 35 % Re, 62 % P<br>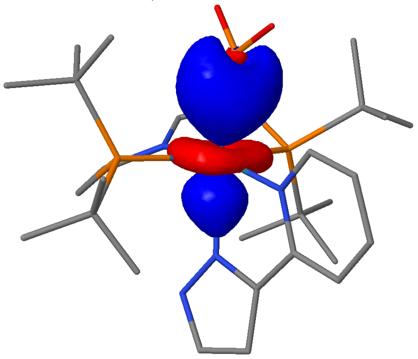	Re–N bond<br>Wiberg Bond Index: 0.598<br>NLMO: 21 % Re, 78 % N<br>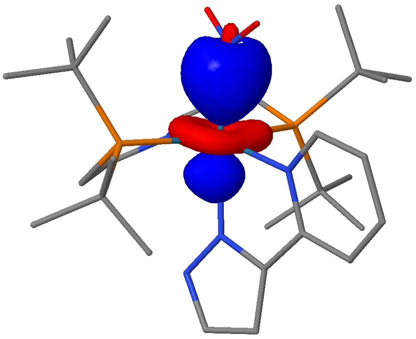
Re Lone Pair 1<br>NLMO: 90 % Re, 1.9 % P (2.5 % PO_2)<br>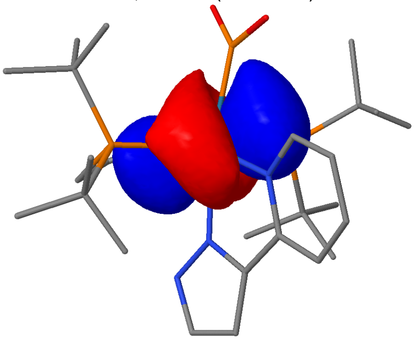	Re Lone Pair 1<br>NLMO[a]: 82 % Re, 0.7 % P (0,9 % PO_2)<br>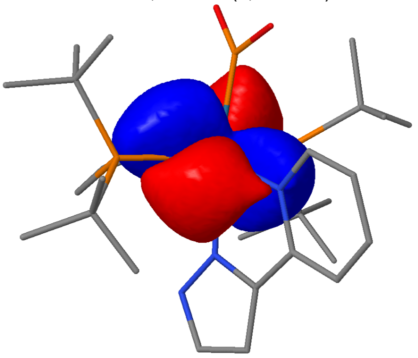	Re Lone Pair 1<br>NLMO: 89 % Re, (1.0 % NO_2)<br>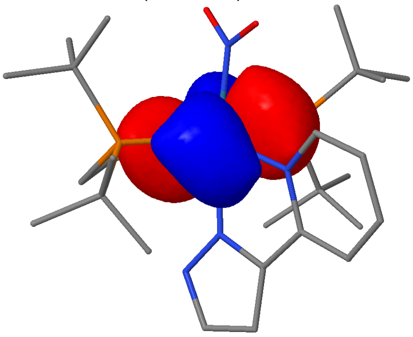
Re Lone Pair 2<br>NLMO: 88 % Re, 3.0 % P (4.0 % PO_2)<br>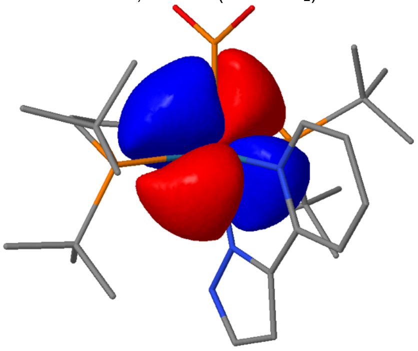	Re Lone Pair 2<br>NLMO[a]: 86 % Re, 5.6 % P (7.5 % PO_2)<br>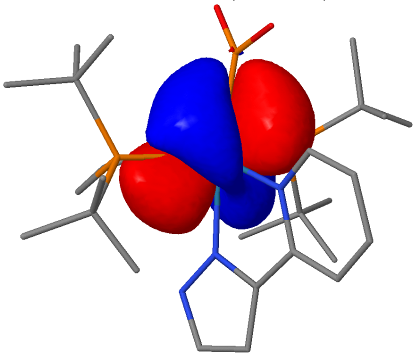	Re Lone Pair 2<br>NLMO: 86 % Re, 3.8 % N (8.9 % NO_2)<br>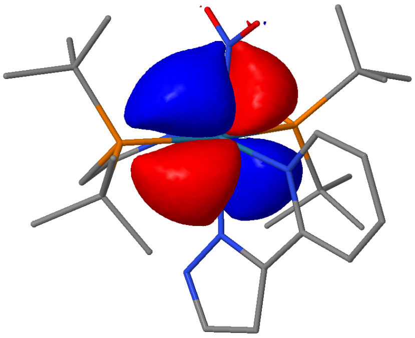
	Re Lone Pair 3[b]<br>NLMO: 94 % Re, 1.4 % P (1.8 % PO_2)<br>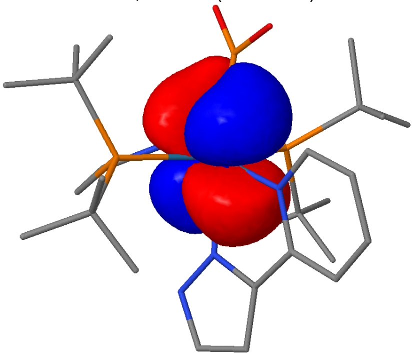	

[a] Averaged values of the α and β spin orbitals. Depicted are always the α spin orbitals. [b] Only occupied in the α space.

Thermodynamics of the equilibrium of 2 and 2_2

The free energy of the equilibrium between **2** and **2_2** is either slightly exergonic, thermoneutral or endergonic depending on the applied density functional. The transition state of the reaction could not be located. Taking into account, that in solution the ^{31}P signal of **2_2** cannot be detected, a ratio of at least 1:40 appears reasonable. This corresponds to a free energy of ΔG = +2.2 kcal·mol^{-1}, which is in good agreement with the computed value of method 3.

Table S10. Computed thermodynamics of the formation of 2_2.

**Method/ functional**	**ΔE (in kcal·mol^{-1})[a]**	**ΔH (kcal·mol^{-1})**	**ΔG (kcal·mol^{-1})**
Method 1: PBE	-16.7	-17.2	0.4
Method 2: PBE0	-19.8	-21.0	-2.1
Method 3: M06L/ M06	-16.4	-18.0	1.7

[a] SCF energies including zero-point energies.

Computed IR and NMR Data of 2, 2_2, 2^K and 3^K

IR

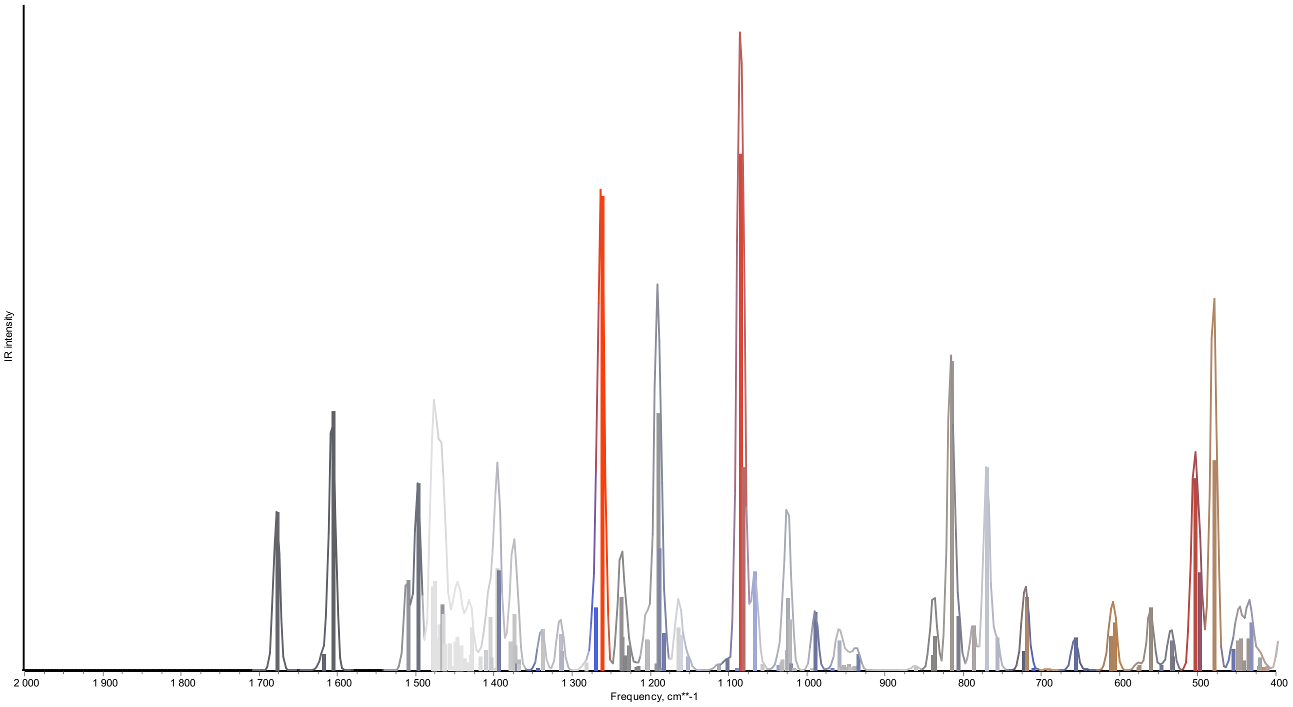

Figure S35. Calculated IR intensities (RI-M06L/def2-SVP) of complex **2**. IR bands are highlighted according to the atom type (square mass-weighted) participating in the vibrational motion: H - light grey, C – dark grey, N – blue, O – red, P – orange.

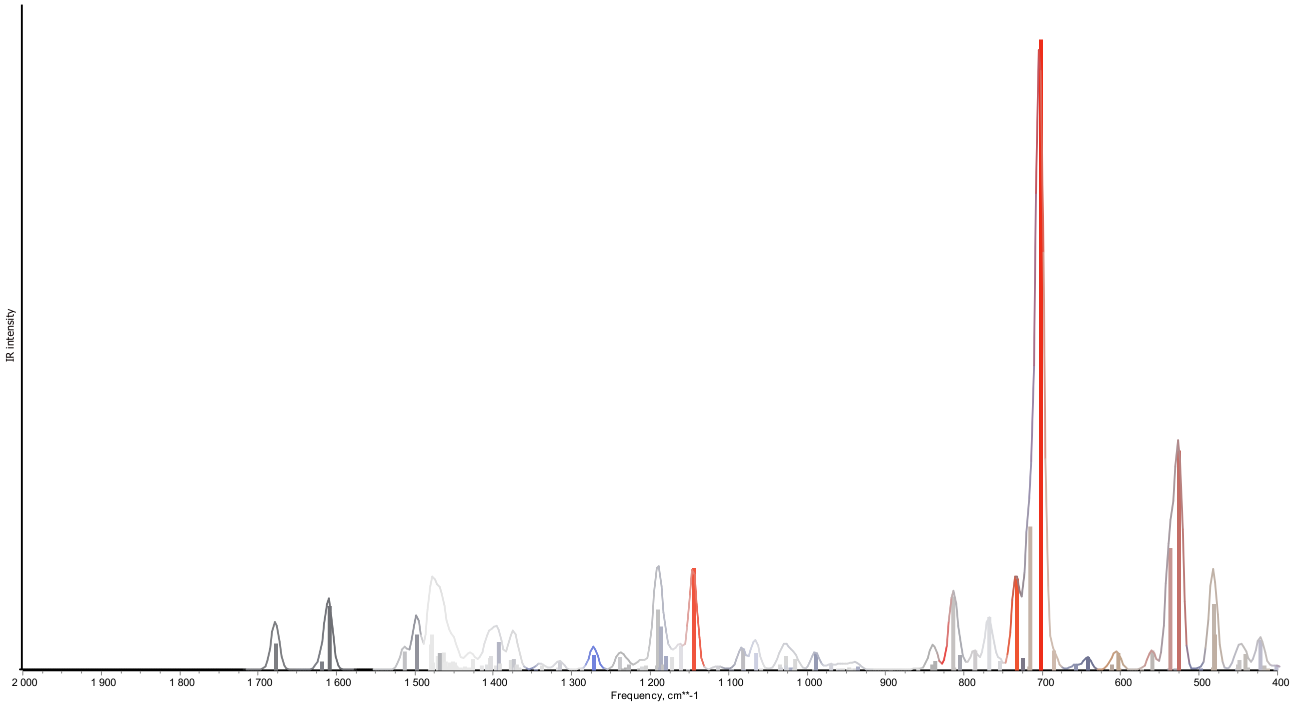

Figure S36. Calculated IR intensities (RI-M06L/def2-SVP) of complex $\mathbf{2_2}$. IR bands are highlighted according to the atom type (square mass-weighted) participating in the vibrational motion: H - light grey, C – dark grey, N – blue, O – red, P – orange.

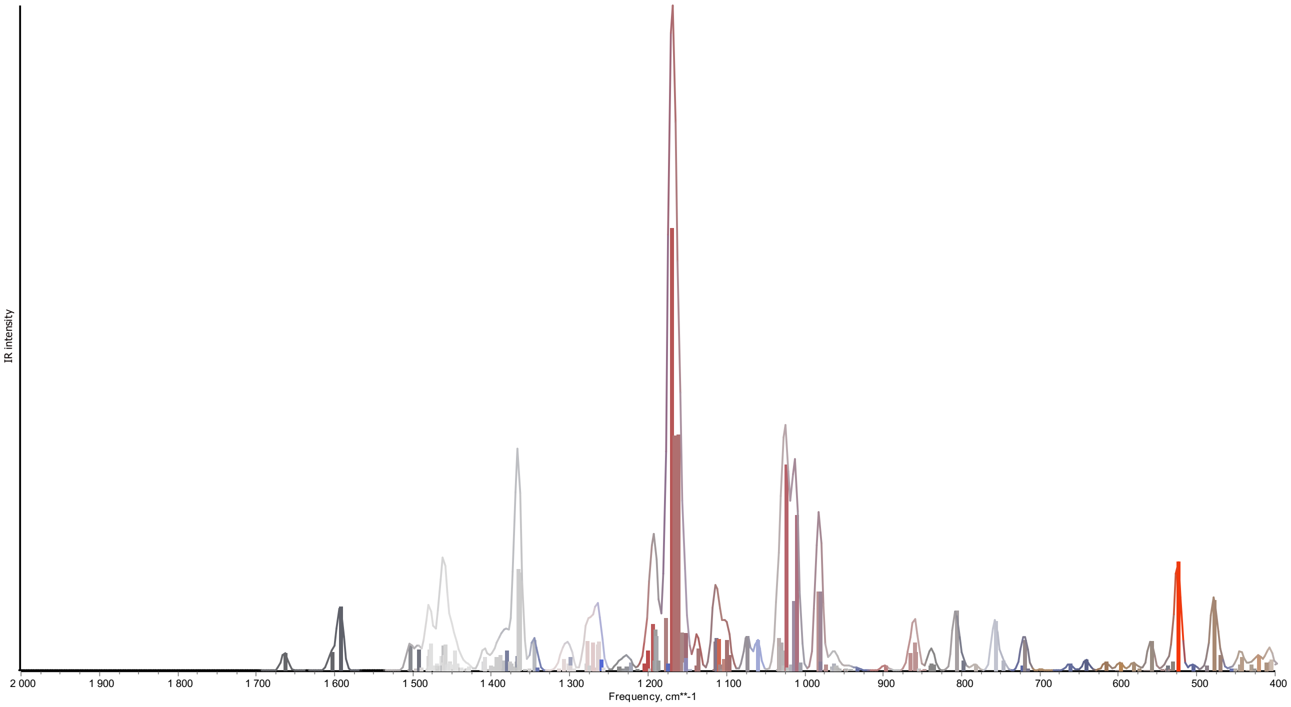

Figure S37. Calculated IR intensities (RI-M06L/def2-SVP) of complex **2**$^{\mathbf{K}}$. IR bands are highlighted according to the atom type (square mass-weighted) participating in the vibrational motion: H - light grey, C – dark grey, N – blue, O – red, P – orange.

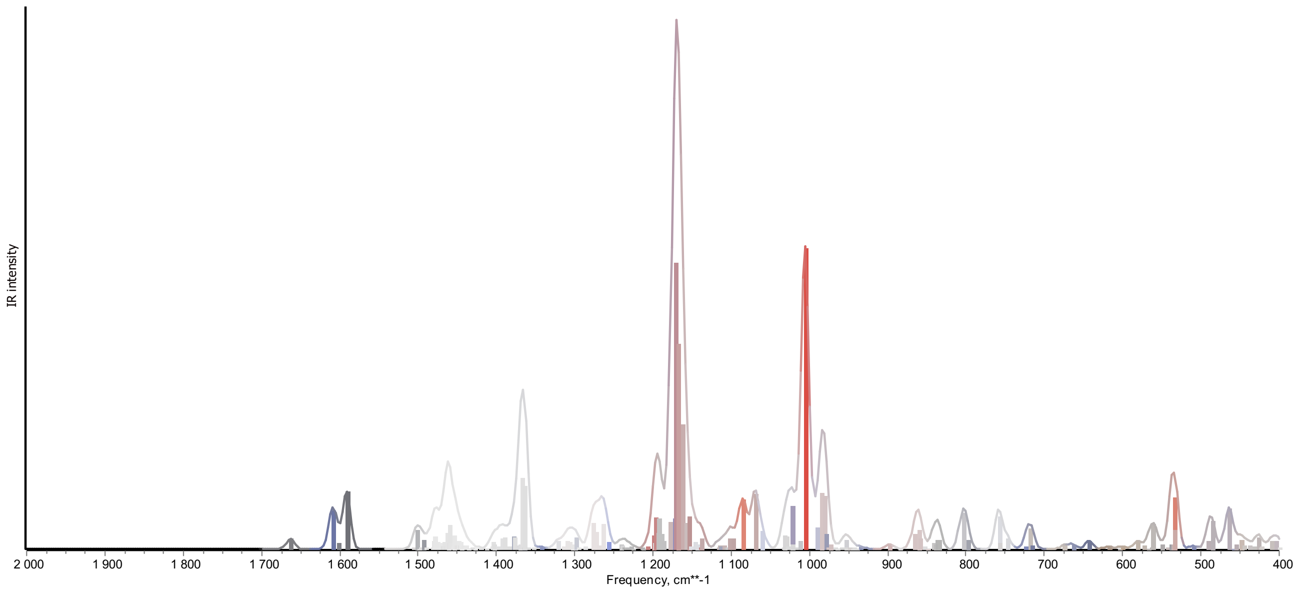

Figure S38. Calculated IR intensities (RI-M06L/def2-SVP) of complex **3**$^{\mathbf{K}}$. IR bands are highlighted according to the atom type (square mass-weighted) participating in the vibrational motion: H - light grey, C – dark grey, N – blue, O – red, P – orange.

Table S11. Experimental (*black*) and calculated (RI-M06L/def2SVP, *red*) calculated vibrations of the Re-PO_2 moiety in **2**, **2**^{K} and **3**^{K}. Analyzed are the symmetric ($\nu_{PO2, sym}$) and asymmetric ($\nu_{PO2, asym}$) stretching as well as the bending mode (δ_{PO2}) of the PO_2 group.

Vibration	**2**	**2**^{K}	**3**^{K}
$\nu_{PO2, asym}$	1245.1 / 1262.8	≈1096 / 1110.8	1060.3 / 1085.4
$\nu_{PO2, sym}$	1078.8 / 1085.7	1017.2 / 1024.8	1014 or 1000 / 1005.6
δ_{PO2}	506.0 / 504.7	538.9 / 524.3	536.8 / 535.0

NMR

Table S12. Computed ^{31}P NMR chemical shifts of **2** and **2**$_2$.

		**Experiment (in ppm)**	**ORCA (in ppm)**	**ADF (in ppm)**
**2**	P_{PNP}	13	78[a]	46
	P_{PO2}	246	311	260[a]
**2**$_2$	P_{PNP}		81[a]	43[a]
	P_{PO2}		187[a]	122[a]

[a] Averaged values.

XYZ coordinates

2

```
88
E(SCF) = -2565.534491, E0 = 0.733269
  Re  12.40741567163910      5.18554917209281      2.35811339318412
  P   12.45485237839379      3.47016854857649      0.59685976246287
  P   12.97714068846732      6.77051040170241      4.13871373171531
  P   12.43338417046729      6.94426304614683      0.72506075643457
  O   11.22479798115326      7.70561280721749      0.25788014738066
  O   13.78026143522448      7.27432871821657      0.14436624889025
  N   14.28283708320672      5.24052334457618      2.04259139347422
  N   10.13656429390617      5.33041931421591      2.51303923131884
  N   11.77693551742503      3.75693241598857      3.79856642740255
  N   12.51408050964475      2.90797483693137      4.49489122043091
  C   15.00454477783222      4.46174567690991      1.02452801039098
  H   15.54689330544706      3.65651459804963      1.54759972094144
  H   15.76500417177868      5.11172139677752      0.56512021677991
  C   14.11360790847981      3.91196120846678     -0.07244215056268
  H   14.60090802136403      3.07665713175687     -0.59702535525869
  H   13.93797328465794      4.71843497903010     -0.80073669127582
  C   12.65736073337851      1.63845157060472      1.10235472301829
  C   12.89555430708179      0.68638880292073     -0.07063906333878
  H   11.99287130541369      0.52405541185419     -0.67411955155959
  H   13.18838363073379     -0.29473679006988      0.33918550986348
  H   13.70899438656258      1.01572435688843     -0.73408509291196
  C   11.43132546885629      1.14591537419843      1.87499368758259
  H   10.53371813384253      1.10236335345447      1.24655965592371
  H   11.20424393492626      1.76384280519936      2.74685187664405
  H   11.62975902913702      0.12323278721978      2.23569456101130
  C   13.89571450753080      1.56282216927408      2.00449162309970
  H   14.82179905022789      1.69615680897719      1.42656118863890
  H   13.93933140600769      0.55655712799408      2.45351251499991
  H   13.86096510322987      2.27739259463210      2.83777879073191
  C   11.33406260947651      3.52799431800027     -0.94943579020256
  C   11.97061989063271      2.90812206039140     -2.19789456256558
  H   11.28722520276392      3.06691836988113     -3.04853616302569
  H   12.14728184334606      1.83171012781075     -2.12030475736110
  H   12.92087663427730      3.39776437223334     -2.45705415818823
  C    9.99731699895866      2.85412065139020     -0.63271832918317
  H    9.27766734962476      3.08503849931979     -1.43532660629119
  H    9.57060074048820      3.22516724875937      0.31240865271960
  H   10.07926759183179      1.76073678855717     -0.57222785913631
  C   11.05310748226802      4.99039746220846     -1.27673350012518
  H   11.96905660647186      5.56281542714949     -1.48941321374979
  H   10.50620035274112      5.50242672001900     -0.47996315710037
  H   10.43317197930231      5.03879416215346     -2.18715709440715
  C   15.22033833011046      6.22804446619917      2.59763161509439
  H   15.74185264174217      6.68842531206174      1.74434708630685
  H   15.97550929410106      5.67525874996522      3.18311243217349
  C   14.57537313793854      7.33241890070115      3.41555566927103
  H   14.31629477273896      8.15702270875805      2.73424586128449
  H   15.27316233691621      7.72697832152884      4.16877602794139
  C   13.49563984769558      6.07083981714366      5.83798000465740
  C   14.57850429650047      5.02131194127393      5.55441014351305
  H   14.25121910463551      4.25447901918290      4.84061636832535
  H   14.81126736614366      4.50004132558581      6.49757609151194
  H   15.51106653204015      5.48869376663080      5.20760926843893
  C   12.31060530586855      5.41450109237951      6.55484826879900
  H   11.70069829694040      4.78807159784668      5.89811425431622
  H   11.65153949447892      6.15898183992763      7.01830207209041
  H   12.69353962209833      4.77057765530272      7.36272958431344
  C   14.10679344088600      7.10932312719277      6.78057565287430
  H   14.90413052723702      7.70474817690188      6.31127699804453
  H   14.55899249918237      6.57675813542222      7.63381629132761
  H   13.35509660546769      7.79372369881748      7.19468275103507
  C   12.01100752780937      8.38688577974797      4.42782392092125
  C   11.49476064693838      8.87287706280669      3.07881374201951
  H   12.30266585778378      9.09714841498397      2.36642054133132
  H   10.93794937412515      9.81225615344287      3.23082129681628
  H   10.81699421577249      8.15751449738953      2.60468005095859
  C   10.80034092524605      8.08915765778316      5.31461537111422
  H   10.11219709962645      8.94993857871343      5.28922903067686
```

```
H   11.07804769402325     7.92308853982598     6.36337919228927
H   10.24467848444281     7.20760824561030     4.95768523165186
C   12.85447973896047     9.51973353115424     5.01874115973859
H   13.73537170545723     9.73982194702079     4.39755485724909
H   13.19205404937969     9.33365279525621     6.04260080995728
H   12.24042488176902    10.43541156652392     5.03734619297287
C   9.32752472177126      6.14911003383344     1.81468836882409
H   9.82412898318318      6.83006725144767     1.11276409705286
C   7.94844056388850      6.16381502496379     1.96538064446180
H   7.36029774959685      6.85621137631882     1.36132587138008
C   7.35172540964873      5.30173836799879     2.88435067931870
H   6.26893105767067      5.29138037977039     3.02849516714441
C   8.17099353013161      4.45674284206287     3.61375239484539
H   7.76451523425103      3.75995666188834     4.34778228636754
C   9.55576067144725      4.48591931748232     3.41022080005840
C   10.46731982015805     3.63126551959052     4.11756132961861
C   10.35296437422363     2.62888382760891     5.09087560274366
H   9.45270350459947      2.26233581392997     5.57695034468157
C   11.67353229155988     2.21477804152874     5.28196340002053
H   12.06119275561162     1.44861134081626     5.95245982566970
```

2⁻

```
88
E(SCF) = -2565.787948, E0 = 0.756371
C  -1.73426224775023     0.10792203644926    -1.50332649296816
C  -1.45560568833113    -1.28661830069234    -0.94831971183341
N  -0.05098478425456    -1.57145381829821    -0.78345742262614
H  -1.92014626617767    -2.05724016191017    -1.59949465834173
H  -1.97164051246093    -1.37915628806346     0.03265901426831
C   0.13201969537867    -2.96530049328320    -0.46267386845311
C   1.56310287211066    -3.31842832437274    -0.06928989154593
H  -0.15877807988592    -3.59420658440306    -1.33320899280939
H  -0.56406269207828    -3.25818001506070     0.35233060751405
P   2.45610763178594    -1.81859933433801     0.53736576887456
H   2.11408475669119    -3.60157459070187    -0.98065704994475
H   1.59218755163144    -4.16289819064029     0.63761904412896
P  -0.26349541317711     0.71522224184477    -2.44221619678041
H  -1.82927618417000     0.79109549886400    -0.64938537441015
H  -2.66549230376810     0.13361427167132    -2.09181191632727
C  -0.45855718244605     2.63842207206181    -2.53319500917268
C  -1.33217608912745     3.14440312756555    -1.38063650676333
H  -1.02769938331204     2.78002480239398    -0.39027899835802
H  -1.27505491643888     4.24651203990319    -1.36043602430303
H  -2.38809497356170     2.86846680559836    -1.52517723438024
C   0.94984617492024     3.21865822836092    -2.38318937798500
H   1.65081060758559     2.80887369504666    -3.12656511733025
H   0.91050288875656     4.31423221848492    -2.51312036796552
H   1.35650133605599     3.01357232865353    -1.38544421720161
C  -1.07042806402475     3.20008120912330    -3.81624194115186
H  -2.11439383249566     2.88896613273380    -3.95335608666345
H  -1.06670240286724     4.30128326236118    -3.74029106898401
H  -0.49899514337638     2.93638194166340    -4.71654622759181
C  -0.56141398426642    -0.00482625582754    -4.19588778958085
C  -0.66053604459288    -1.52657397191422    -4.03147363049418
H  -1.64111474811617    -1.81434329959645    -3.62535072207145
H  -0.57076114468321    -1.99137013518783    -5.02888838019343
H   0.12589065629762    -1.95364763031237    -3.39852032520721
C  -1.85779987163634     0.42662190560447    -4.89356959614638
H  -2.07696497799472    -0.30089790879076    -5.69443364180259
H  -1.79728308669789     1.41239417100219    -5.36546433579075
H  -2.72115678925223     0.42091772444881    -4.20971616033774
C   0.64329894361735     0.33259194005383    -5.07451182345983
H   0.49806504158865    -0.09334518013802    -6.08291006374636
H   1.56884921017808    -0.09042577678819    -4.66398611263406
H   0.78876890121052     1.41774220723882    -5.18914871320577
C   1.96953237221439    -1.68903317377937     2.38855439677153
C   0.43762170374881    -1.70190366623796     2.46574250599816
H   0.03219528582449    -2.68547696138166     2.18963470666291
H  -0.03940807776911    -0.93219147196239     1.84450142494126
H   0.14457124212343    -1.50891859732463     3.51207545752991
C   2.49031007219644    -0.37753955624150     2.98825282881688
H   3.56710930401384    -0.41479068111791     3.19719039276619
H   2.31190115733757     0.49536000221906     2.34481670824154
```

```
H    1.97374882200518   -0.18899633206075    3.94443634673732
C    2.46392768002040   -2.85408414695209    3.24863556463434
H    3.55498220952575   -2.86407204072418    3.37483883503322
H    2.02237784243749   -2.75702927361358    4.25566468297492
H    2.14886795326653   -3.83070731842646    2.85000423756470
C    4.28732932662925   -2.39472341731259    0.49848878674036
C    4.48619864204523   -3.86630432857293    0.87226198828007
H    3.95741341150995   -4.52959457042486    0.17272552159199
H    4.16161606275997   -4.11360476810103    1.88911750989954
H    5.56148161064087   -4.10844990519006    0.79992222950762
C    4.79645555139057   -2.21761253866492   -0.93031451370636
H    5.83888940521749   -2.57847597480984   -0.98055442695879
H    4.79053323444516   -1.16256609496247   -1.22426964524568
H    4.20818060434251   -2.78161784138994   -1.66639339006557
C    5.14157378760939   -1.49330113265549    1.39084586049931
H    6.20833162635280   -1.68312274742620    1.17936742987765
H    4.98296488175510   -1.67424074550502    2.46228538419521
H    4.94585255462183   -0.42923041242863    1.18583423832649
Re   1.47528597572200   -0.24846260431682   -1.03504924303373
N    3.19266376533059    0.92631943057647   -1.64558662470164
C    3.66829871316603    2.02071938598401   -1.00768102558494
C    4.74151883553039    2.76123969165357   -1.47373683516768
H    3.11775124224388    2.30159074431653   -0.09932778124561
C    5.37785311249257    2.38720462894254   -2.66239546129233
H    5.06146392515741    3.63598015437994   -0.90414118506024
C    4.92227297348899    1.24994614287681   -3.30951660828406
H    6.21465278062219    2.96385061954051   -3.06425463886922
C    3.84997384750413    0.52278418275523   -2.77810906033959
H    5.39187436940006    0.88869894298673   -4.22619910092660
N    2.19343789437319   -2.56673667600002   -3.14121942138458
N    2.40140150458253   -1.35399440301422   -2.64314636636408
C    3.06365432533037   -2.73008682921305   -4.15634197029591
C    3.86477892642343   -1.59806329719405   -4.32066009662376
H    3.07066057761438   -3.66109298975393   -4.72496361526673
C    3.40394222860227   -0.73430667823429   -3.31365929610368
H    4.66329139441294   -1.42931073597119   -5.03979805456501
P    0.72807381774369    1.23135929863921    0.59373966560122
O    1.57978194701893    2.40613004164676    1.05146997749143
O   -0.65272988389036    1.13606504363807    1.21684829417839
```

2[K]

```
131
E(SCF) = -4086.877799706045, E0 = 1.13198386
  Re  0.91167679744689    15.18441291202869    11.68905672449186
  K   2.84700809843265     9.99221353571670    11.65458099951130
  P  -1.20897910535841    13.97402300092599    11.82075972207047
  P   2.82687140039036    16.44633033208526    12.54648772801051
  P   1.95334852962608    13.17231304811014    11.79404345330031
  O   2.90945796238007    12.65116471569648    10.70740222847224
  O   1.74027834459336    12.10614370703776    12.88166501133671
  O   5.30131644818734    10.41213350047864    10.14415969468808
  O   3.01884810664947     9.20142311977713     8.88019736809967
  O   1.43699824989456     7.63503997727540    10.57958889167293
  O   1.09736863424421     8.38658202697057    13.24428197270620
  O   3.08953688739583     9.78753975824612    14.64756950035812
  O   5.02168190938623    10.98896560633258    12.93085720990846
  N   0.48822901481891    15.31122378922946    13.66523729239890
  N   1.02563926755457    15.39073289551999     9.52656851087095
  N  -0.04133364200976    17.05790019826422    11.23433733380684
  N  -0.52298499149538    18.02489172793631    12.00151486310090
  C  -0.28060079720157    14.34590485831899    14.41821625496435
  H  -1.00525075742164    14.87595274840044    15.06616528074184
  H   0.39088765733036    13.79583383689686    15.11369200844329
  C  -1.01042154001886    13.32815765265904    13.54346656737721
  H  -1.96899796405795    13.02450174009071    13.99249499474770
  H  -0.37511602713365    12.43804507610081    13.43209064462473
  C  -2.86599267709033    14.93805481121978    11.92295507733856
  C  -4.10826152885953    14.08458655776721    12.19038369370435
  H  -4.38274106192351    13.42927950427310    11.35313925595750
  H  -4.95986372276194    14.76678005490274    12.35595268743345
  H  -4.00942110149842    13.47189058239359    13.09968554004795
  C  -3.09207863891192    15.76083140061672    10.65141429837362
```

H	-3.32021623372134	15.13782108903434	9.77745991483382
H	-2.22640913560282	16.38584774239933	10.40794417872311
H	-3.95240865876708	16.43305982624863	10.80767021933486
C	-2.72245311005737	15.89259331235255	13.11374565629450
H	-1.78428874104355	16.46147709691832	13.09050927518479
H	-2.79925722068723	15.35109765109150	14.06779659094574
H	-3.54823378340665	16.62245939070911	13.08251706038463
C	-1.57934860106398	12.38736532149258	10.79368306747527
C	-0.27570894647740	11.71631126335059	10.36786775708992
H	-0.51551536048039	10.83356375796506	9.74861711620067
H	0.29658865343165	11.37225990809639	11.23790649395094
H	0.35870737052326	12.38005058877541	9.76768932636126
C	-2.36329651071764	11.34692671090993	11.60085502977209
H	-2.60795511402480	10.49380510374193	10.94346334747808
H	-3.30270733310067	11.72364180351485	12.02069166165792
H	-1.75580939438176	10.95624225453819	12.43079398169101
C	-2.30996013300297	12.76340075388516	9.50227680694204
H	-1.76736029203983	13.55262614717022	8.95876333148646
H	-3.34043591405462	13.10334281888805	9.66418330662140
H	-2.35547011517331	11.87748208986492	8.84634528759194
C	0.84348853415871	16.44682563449029	14.48907020693496
H	-0.06058794407659	17.05587041022663	14.69847518039356
C	1.89723704137460	17.35253021856692	13.85596874770629
H	2.54888059786236	17.80891600844134	14.61668540157185
H	1.38136570225098	18.15769093188554	13.31052220527646
C	3.70447190867113	17.85046031117966	11.58540996534629
C	2.71671851867251	18.36332554782217	10.53848635011230
H	3.19601234880376	19.17768417161754	9.96947425190741
H	1.79519559158105	18.76308528691909	10.98147645188696
H	2.44272374829408	17.57027327742044	9.83342011963332
C	4.12050482396772	19.03915508772962	12.45624634760562
H	3.25098975903173	19.51952696502508	12.92662319845706
H	4.60053514226123	19.79532892256863	11.81202523323860
H	4.83833702946009	18.78091822211534	13.24331769535065
C	4.91562181644405	17.30173370490832	10.83172102886430
H	4.65434113996803	16.40436591727223	10.24979763813393
H	5.75496562792685	17.05899924614477	11.49613280069401
H	5.27233976884260	18.06587196937773	10.12141068660982
C	4.18314292065415	15.50687740692213	13.52992661431926
C	3.48701787912772	14.64639851006365	14.58847389969086
H	2.72224443251011	13.98498028368360	14.16126109385681
H	3.01495394283349	15.26076332825322	15.36622554440036
H	4.25197566705002	14.02521065565789	15.08590447720566
C	4.99415842758793	14.57952690555264	12.62034119542917
H	4.35611932968312	13.93267144126578	12.00392288572024
H	5.62235970113818	13.92666836750375	13.25007322564066
H	5.66489704620415	15.12704987639667	11.94904080013186
C	5.14097989891188	16.43886168929786	14.27396827018435
H	5.78999778341138	17.01554495207042	13.60211878119596
H	5.79976844345479	15.82782611924155	14.91509328953749
H	4.60766535011659	17.13985510864211	14.93337224618522
C	1.66985271527374	14.54678481196938	8.69098689423446
H	2.21886314921048	13.73700494506138	9.18323620601647
C	1.66573193967364	14.69472011213996	7.31470298424697
H	2.21522339414155	13.97745210126971	6.70253187766657
C	0.96140439427724	15.75974633598674	6.74123602684135
H	0.93568149951590	15.90499493860094	5.65880941820521
C	0.30870104209608	16.64147870953252	7.58555721306427
H	-0.24382415251248	17.49752437863622	7.19536271193247
C	0.36736718720669	16.45490624392587	8.97306483470898
C	-0.19677590512430	17.37209671178712	9.92324859301844
C	-0.82578930742690	18.62328798113493	9.84179724220157
H	-1.10607985067685	19.18271663951695	8.95269351415873
C	-0.99990607354581	18.97940301116725	11.18119942384061
H	-1.45476707120369	19.87818994720142	11.59860351413945
C	6.22740425322781	11.09790415462855	12.23049316499255
H	6.79094606312146	10.14353927725062	12.26127621115823
H	6.86848223086735	11.87970955621456	12.68194982499002
C	5.92478659150072	11.48537579534871	10.80755416670276
H	5.25066262214362	12.35858584924099	10.81000504257135
H	6.86825942634986	11.76291726966426	10.29438412389174
C	4.79490714768857	10.78884493298243	8.88763728227351
H	5.60746052252432	11.18151143377822	8.24323108940368

H	4.03119782381574	11.57930997970617	9.00809531192610
C	4.19991451882217	9.58070407587143	8.22178612920114
H	3.98861677066400	9.81597502331967	7.15963203090893
H	4.93711823933351	8.75474007001217	8.25331526581835
C	2.55946986502410	7.93102818958415	8.52189382656731
H	3.28252451716840	7.14991642594709	8.83153331272871
H	2.42524386074061	7.84283046973256	7.42517053277136
C	1.23968002775940	7.68426245598786	9.19448905419060
H	0.53210699669477	8.49444122922454	8.92905575519037
H	0.80997781523980	6.73134134380031	8.82520480911915
C	0.25391872704007	7.37400988786625	11.28790809933373
H	-0.23444751788367	6.45483387157352	10.90661976266195
H	-0.46364946673835	8.20929182798750	11.16473463463324
C	0.56750311961837	7.19515867220638	12.74769192808545
H	-0.36905168855703	6.92296902961075	13.27505124956112
H	1.27960083844883	6.35722736290610	12.88558147783401
C	1.14807238115997	8.46484181774987	14.64411521988846
H	1.77078489836404	7.64795046288438	15.05801012066238
H	0.13062929856666	8.36378422922824	15.07150879868101
C	1.73895076716091	9.79447634614133	15.02476138014951
H	1.20177231880638	10.60867113430975	14.50592749801554
H	1.62923859106861	9.94642939764892	16.11845911708985
C	3.75612992791403	10.99239087922913	14.92623432675913
H	3.83858025709792	11.14965892302368	16.02135841905040
H	3.20115000453403	11.83549824036804	14.48247570030832
C	5.13383602062464	10.93234853750738	14.32595790261294
H	5.72836811171243	11.78984948613331	14.69829865916830
H	5.64694265051472	10.00309666008422	14.64606202585106
H	1.19807135003667	16.09049504882191	15.47723623916038

2^{NO2}

88

E(SCF) = -2279.140086, E0 = 0.762415

C	-1.72653142820803	0.00540046000165	-1.63255631411071
C	-1.39938449037053	-1.41245713268975	-1.19316116022875
N	0.00599086205933	-1.61314951569501	-0.83645143488692
H	-1.68249253895070	-2.15217193380265	-1.96118481386605
H	-1.99587288930576	-1.66088191496061	-0.29567954615693
C	0.19717042331832	-3.01273523245740	-0.44462006760098
C	1.59374815870958	-3.34166170436788	0.06001964329471
H	-0.01904711689702	-3.63578110122883	-1.33030205119676
H	-0.56538075044526	-3.25705507501176	0.31450455893933
P	2.46031026070589	-1.81990960751627	0.63589548145526
H	2.19119909349383	-3.69686978954330	-0.79292823849107
H	1.56484181400897	-4.13369177853213	0.82327490079715
P	-0.26451502146979	0.75423201053681	-2.47158900336449
H	-1.88759059449005	0.61907924604515	-0.73702204434535
H	-2.64138706072032	0.02968751122230	-2.24384507357398
C	-0.48562748989815	2.65907182451006	-2.36981164018446
C	-1.25840435169310	3.04966199810707	-1.10449645192332
H	-0.83675324469847	2.64618287794296	-0.17669019409253
H	-1.23981169359765	4.14919050566032	-1.02415850932092
H	-2.31497503109210	2.74807682608062	-1.16514047601916
C	0.92457772388065	3.24795307277378	-2.29398079894245
H	1.56402142509055	2.92226029091334	-3.12862552816016
H	0.86243853466519	4.34837530666736	-2.32502178618895
H	1.40993267238585	2.96555588559469	-1.35352120279504
C	-1.21716279523990	3.30489127278064	-3.54552498805915
H	-2.24574958367590	2.93763299135719	-3.65908064086953
H	-1.27753403211573	4.38769949127357	-3.34671167246380
H	-0.68592970969763	3.18320406357076	-4.49828583172694
C	-0.45906103180459	0.19948580517260	-4.28723605134160
C	-0.49576852285305	-1.33380369709112	-4.28874461147662
H	-1.48508256656019	-1.70330948638720	-3.98214443105740
H	-0.32969387220776	-1.68597859426879	-5.32020517046250
H	0.26531827621942	-1.80363590110417	-3.65754357922742
C	-1.74885076435105	0.64808429232175	-4.98682947393662
H	-1.90171440315470	-0.00063292221817	-5.86507741555403
H	-1.71839086519589	1.67865599528026	-5.35144513152419
H	-2.63753570933630	0.53274153840223	-4.34739571805601
C	0.75932203425272	0.68692904622288	-5.07171845153158
H	0.68058984691763	0.34963734883789	-6.11879545918506
H	1.69031555828705	0.27419198241275	-4.66182438861225

H	0.83906605989936	1.78402418926101	-5.08208358428917
C	1.92124772075075	-1.64951272321380	2.45956823771719
C	0.39386089030307	-1.78441697092593	2.50507690943942
H	0.07273088852038	-2.81738556782815	2.30779951637676
H	-0.11141436001094	-1.09872632635842	1.81277405580600
H	0.05596620168176	-1.53050375790351	3.52329909050605
C	2.31101260357304	-0.27799569764530	3.02245049531703
H	3.39002376905178	-0.08758477319182	2.96825102714179
H	1.79633015771254	0.55147009205997	2.52573057373966
H	2.02653042130947	-0.24822415966552	4.08772588963882
C	2.49744525879201	-2.73997082554609	3.36540465515879
H	3.57844901570789	-2.63675490188991	3.52729939728700
H	2.01324538388408	-2.65952752300825	4.35285218390318
H	2.29485113571572	-3.75382096211594	2.98897310243332
C	4.30187795453562	-2.31151334125251	0.59961032896874
C	4.55586866887822	-3.75430958550993	1.04574969663581
H	4.02332478660391	-4.47445422671771	0.40800992211864
H	4.28358672213354	-3.94932808923005	2.08806927981736
H	5.63312414030425	-3.96731919995182	0.94197089933004
C	4.79109220998384	-2.18466648433043	-0.84214173647274
H	5.84719860924207	-2.49920166604002	-0.88192388074389
H	4.73782368489118	-1.14755207196147	-1.18940427458085
H	4.22843696348751	-2.81633213381163	-1.54281344866432
C	5.11689528514573	-1.33237017943682	1.44638273166941
H	6.18984630122754	-1.51028065861783	1.26494831772017
H	4.94345048389596	-1.44795704319547	2.52429256741921
H	4.90391272805525	-0.28777492197643	1.16990884541692
Re	1.40027867932186	-0.33761110822994	-1.00577977229518
N	3.16836710869804	0.82044490717655	-1.79719513774422
C	3.68254199445639	1.93640027720079	-1.24825327407013
C	4.73683452981512	2.63877142570448	-1.80969758803780
H	3.19783741332268	2.25488459361216	-0.32327204970407
C	5.29468601900196	2.18574913957307	-3.00695610114231
H	5.10436045603562	3.53565335448304	-1.30925639868219
C	4.80258927521762	1.01601464220450	-3.56024957075543
H	6.11185334736408	2.72835625995011	-3.48760474106819
C	3.75802920637936	0.33479730327191	-2.92301186413757
H	5.22312848263619	0.59501105763479	-4.47430091991793
N	2.06859091384172	-2.74879171101225	-2.93565717283610
N	2.31564105244077	-1.50100872382175	-2.55858661289984
C	2.87935795713714	-3.01046380633402	-3.97788568465632
C	3.67769170731284	-1.90667187099138	-4.28368456434317
H	2.84791999108072	-3.98600594757092	-4.46274334486689
C	3.27949615581063	-0.95506422737607	-3.33513540253714
H	4.43915538895172	-1.81444646090827	-5.05416350075645
N	0.82185333406639	1.01848205245059	0.43015442663315
O	1.55825542458278	1.96271676227581	0.74311986812115
O	-0.26216124871491	0.89713533389711	1.00576337293398

2_2

176

E(SCF) = -5131.148049, E0 = 1.470346

Re	12.45700605742991	5.33200130869161	2.27382512081545
P	12.55480827294384	3.59849696349519	0.52301711908509
P	12.98702042832070	6.85332290153214	4.12563784237838
P	12.47037414017897	7.10273599189654	0.61687878530825
O	11.19752486584093	7.81988459125939	0.22229953511901
O	13.69977730323402	8.25080135881741	0.82470197839806
N	14.34108260705285	5.36623705319019	2.02351733573598
N	10.18447301574743	5.53132335663656	2.53681240212730
N	11.82442385026804	3.86007100335831	3.67989958516681
N	12.56131272211275	2.96541041915799	4.31343974593570
C	15.10290477326429	4.55651413376991	1.05574392056107
H	15.60796064475282	3.76609985199046	1.63567295917229
H	15.87433264543832	5.20098140920214	0.60842687234739
C	14.25503613145622	3.99294763900001	-0.06692982688167
H	14.75135054639821	3.13680963694850	-0.54452097481580
H	14.13649949081970	4.78280729508378	-0.81447687804558
C	12.77705460695842	1.76018744718772	1.00211245982505
C	13.11137843887188	0.87111553549645	-0.19885764726973
H	12.27543550426841	0.73125739678465	-0.89439773103943
H	13.39211986465683	-0.12654321598925	0.17898145450913
H	13.97435170458925	1.25042728048079	-0.76653533340901

C	11.53615493453437	1.21681267496244	1.71789040928355
H	10.71048116273622	1.02610934239516	1.02329977455332
H	11.17074774169676	1.89159535831795	2.49938213050074
H	11.78529403966114	0.25771459428571	2.20071068570058
C	13.99632231611346	1.68156791812219	1.93264827448587
H	14.93107215582231	1.82164708242538	1.37147319010004
H	14.03330332013402	0.67092250877930	2.37276114156212
H	13.94212834622364	2.38495114475168	2.77313329483920
C	11.44760850740497	3.62158183745936	-1.05010992284988
C	12.25594984229295	3.36162974627127	-2.32369559511692
H	11.58112019974894	3.43320987546509	-3.19386469679850
H	12.73064862633129	2.37251282838977	-2.35123149332326
H	13.04066937450291	4.11648649899185	-2.46290081916681
C	10.29189650576420	2.62425180913247	-0.93575011013221
H	9.59409309930718	2.80935527598369	-1.76820098268904
H	9.72687861809472	2.76099268192874	-0.00178808143292
H	10.60738123747575	1.57659064633518	-1.00257664326483
C	10.80087490357232	4.99717287419259	-1.17555160948414
H	11.53293742295384	5.78132543546931	-1.37894228871142
H	10.24108701781988	5.26967603096157	-0.27295247506209
H	10.09171422314920	4.98359675626205	-2.02011563297176
C	15.29113262304887	6.27253596297920	2.69197432118390
H	15.89164424402638	6.73856267766318	1.89377729941453
H	15.97026005772377	5.63820206271559	3.28753522212787
C	14.65785848519940	7.35821638717214	3.54427931034693
H	14.49137623733840	8.24127303898284	2.91503592270555
H	15.32317303645869	7.64833220931457	4.37003910677488
C	13.38334868433732	6.09884379310736	5.84021301326829
C	14.48266697498114	5.05676969056898	5.60089243593798
H	14.22484167139813	4.33383957846161	4.81666758407253
H	14.62531165182219	4.47948982243269	6.52950794282266
H	15.44252725574071	5.53956997631251	5.36783775375445
C	12.16477131409004	5.42795394748603	6.47813081849762
H	11.61563292320873	4.78458709236576	5.78768333642400
H	11.45844066957831	6.16201989962271	6.88577673705648
H	12.50365316478771	4.80108792817552	7.31868263734621
C	13.93600400299748	7.11164903158305	6.84322940036353
H	14.75935685821076	7.71703860909278	6.43549057330726
H	14.33673685201725	6.55434412900659	7.70677284336942
H	13.15733888200093	7.78247637910783	7.22920222430663
C	12.06603509417872	8.49687249221840	4.42917767646218
C	11.61468512118954	9.03802500433144	3.08062434827585
H	12.46357709540993	9.24420849580635	2.41975232368491
H	11.07568557936170	9.98700223806156	3.23933528686038
H	10.94291805345416	8.35538769455098	2.55353814479603
C	10.81208272616042	8.22028589191379	5.25820850903495
H	10.16634432518012	9.11388176717052	5.24282824659135
H	11.03767866566647	7.99252037056956	6.30821158028325
H	10.22995587279085	7.38557501289512	4.83702948456401
C	12.93041230705331	9.58429147699848	5.07484134632274
H	13.83206494752371	9.79144698276513	4.48103328587336
H	13.24450961258852	9.36024979984989	6.09851427953623
H	12.34562830598801	10.51914428542053	5.10449377576424
C	9.39008333867756	6.45567779504762	1.96716424829026
H	9.86575853808662	7.08356353622065	1.20477106459915
C	8.06086501448695	6.63843486288979	2.31871474736770
H	7.48677685973804	7.42153967810004	1.82194659664815
C	7.49680588143309	5.82460524400544	3.30019872342910
H	6.46149292357178	5.96267901859204	3.62117557852090
C	8.28737332988058	4.83715817131325	3.86397674537403
H	7.90155459960237	4.16407194751990	4.63051030100003
C	9.62437176584403	4.70841481883288	3.46562893470870
C	10.52301557719953	3.74681251035666	4.03784740673996
C	10.41578176137362	2.69770085770897	4.96221962143076
H	9.52290448372017	2.32790892794282	5.45954546094700
C	11.73195568714380	2.24809994112265	5.09096718713668
H	12.12637933165178	1.44109812021505	5.70748211102883
P	14.55768588045293	7.91982668118527	-0.61914195425673
O	15.83089819880816	7.20300073493244	-0.22522087893656
O	13.32848591311084	6.77148272329314	-0.82675962418450
Re	14.57104163472449	9.69158524113784	-2.27475292185119
P	14.47480793141397	11.42364899246769	-0.52194540464835
P	14.03995453421942	8.17087850739690	-4.12677231458155

N	12.68697605359853	9.65834236095394	-2.02439161935176
N	16.84335476313784	9.49214310626440	-2.53929255776537
N	15.20289529313523	11.16436249295967	-3.68026084467665
N	14.46590574165984	12.06038069031448	-4.31187603378270
C	11.92580989146541	10.46977923229221	-1.05755279031852
H	11.42450621880379	11.26229578851820	-1.63781389548251
H	11.15150035021289	9.82766049070633	-0.61185854863614
C	12.77387172446418	11.02986912483756	0.06672093437415
H	12.27822238672637	11.88536727631654	0.54615891542405
H	12.89117500490680	10.23801968509751	0.81233376998445
C	14.25462405730414	13.26289853402265	-0.99817343876318
C	13.92087993305777	14.15031486462625	0.20424377929627
H	14.75657706585914	14.28839292555828	0.90040263300303
H	13.64096443925113	15.14880275688925	-0.17201661797025
H	13.05740284112714	13.77073170420863	0.77097906206906
C	15.49643970639024	13.80542184770930	-1.71271488720338
H	16.32559531690809	13.98490038112704	-1.01938515117206
H	15.85510768161783	13.13525723972769	-2.50123720570156
H	15.25133329887861	14.77015265693315	-2.18637997377188
C	13.03588099849852	13.34518122137828	-1.92904909640706
H	12.10060729693255	13.20692641418884	-1.36824804673329
H	13.00143198228250	14.35631540309381	-2.36825868160297
H	13.08859075378490	12.64245559347816	-2.77018665913419
C	15.58155802358345	11.39751846748364	1.05178878213107
C	14.77189199615969	11.65428290868242	2.32514343414763
H	15.44590149181384	11.58152651104690	3.19584864884343
H	14.29637162282637	12.64297798394655	2.35416021443161
H	13.98763768860262	10.89855694124191	2.46186576182298
C	16.73742532237898	12.39517010080458	0.94136061825170
H	17.43276538072562	12.20933568607102	1.77572086761881
H	17.30516211410917	12.25897581531773	0.00897630418503
H	16.42197857377553	13.44280315917638	1.00824984564212
C	16.22887691909921	10.02202325237626	1.17461646939774
H	15.49727941171877	9.23705509122202	1.37624618066552
H	16.78910212089255	9.75170210477411	0.27163582055046
H	16.93777060117820	10.03422056699623	2.01942506793791
C	11.73633902946923	8.75216293950430	-2.69207538781909
H	11.13709539603185	8.28579469107529	-1.89308401639859
H	11.05631515563558	9.38653482622909	-3.28658907953266
C	12.36877258209453	7.66685800353367	-3.54540376766897
H	12.53477260313349	6.78311266412181	-2.91698641885730
H	11.70319751849467	7.37777844056462	-4.37130705385005
C	13.64365770925532	8.92610618394995	-5.84124519032908
C	12.54480105192987	9.96853563856200	-5.60164783465595
H	12.80327436950489	10.69142033342498	-4.81756887916391
H	12.40199017193777	10.54577727375670	-6.53027744359292
H	11.58480080337984	9.48621568302985	-5.36816641373295
C	14.86178289657517	9.59720227370812	-6.47957653060121
H	15.40349832958718	10.25018055417962	-5.79244565084987
H	15.57417168498170	8.86366018409451	-6.87731868302434
H	14.52305494439285	10.21465223268907	-7.32721279960489
C	13.09083838691808	7.91330825631111	-6.84417674358775
H	12.26733155264388	7.30822737100329	-6.43632132420044
H	12.69014756144245	8.47054523957557	-7.70777538815213
H	13.86936884930936	7.24218778564914	-7.22999181903147
C	14.95881828506523	6.52603275942475	-4.43087778480183
C	15.41234773334120	5.98505251644095	-3.08298949262024
H	14.56446949332491	5.77965558541101	-2.42057502479466
H	15.95038596416579	5.03564598087771	-3.24246219240164
H	16.08558146111059	6.66735225131037	-2.55733135772583
C	16.21137861941538	6.80001747216590	-5.26286520898149
H	16.85604072265441	5.90564043481353	-5.24744749355878
H	15.98391271988057	7.02644848118357	-6.31277092654116
H	16.79553253202695	7.63451365621831	-4.84406200071404
C	14.09156308138220	5.43921740775061	-5.07371390457679
H	13.19340183176153	5.23064845552347	-4.47512589816126
H	13.77163832776211	5.66465978354261	-6.09526543305541
H	14.67662978699184	4.50469501128107	-5.10791863700962
C	17.63797634655031	8.56739143581740	-1.97063436408424
H	17.16263732043639	7.93916422261339	-1.20833838517019
C	18.96701067149753	8.38479038934604	-2.32298098562654
H	19.54144369238719	7.60153868274038	-1.82683416852852
C	19.53051693180586	9.19906759590763	-3.30441071467825

```
H   20.56567292675420      9.06117229962291     -3.62596779690780
C   18.73964056617307     10.18675709288006     -3.86733761652742
H   19.12504950071976     10.86019071423117     -4.63376818517281
C   17.40290576893892     10.31540853112269     -3.46810873650208
C   16.50406057416251     11.27764088460391     -4.03897107128664
C   16.61109558379709     12.32808683820568     -4.96182667703019
H   17.50378763999553     12.69822344717946     -5.45922499020576
C   15.29501713306541     12.77856067000499     -5.08887407775262
H   14.90048343065004     13.58667612661722     -5.70385018717215
```

3⁻

```
87
E(SCF) = -2565.186614874104; E0 = 0.74467618
Re   0.91312574815897     15.10645341529124     11.63959760509032
P   -1.16797345998363     13.90762939124924     11.76476367844324
P    2.80165694808898     16.38376194662278     12.47315634577233
P    2.04429999615786     13.10316806355504     11.77583196968045
O    3.00357986643794     12.63160095192192     10.68892394606631
O    1.87635285438821     12.13088151019869     12.93949866147705
N    0.55202957111338     15.12896963939461     13.68339721979338
N    1.04013638812549     15.30818513332839      9.51242016128141
N   -0.09369590745362     16.95803220810982     11.19044702428726
N   -0.59150554555496     17.93492244468009     11.94239632606818
C   -0.11860678802804     14.00258382876850     14.35130824729260
H   -0.72607413870751     14.39101511857460     15.18638607825262
H    0.68872096680021     13.37687703380244     14.76164360438199
C   -0.94964539580426     13.12050079301605     13.42265460768198
H   -1.90816387883431     12.83845308972012     13.88567563122836
H   -0.36151393184099     12.21215465756874     13.22443724440351
C   -2.82326670617751     14.86345311654090     12.02154755152131
C   -4.03815155896031     13.97267224848144     12.28260144503956
H   -4.36839558142029     13.43744593225901     11.38136999992320
H   -4.87909725839928     14.61323683250565     12.60275318165517
H   -3.86615884211565     13.23610206142217     13.08222075099373
C   -3.14965152062660     15.77993316637647     10.84129422290102
H   -3.38685139869139     15.22493360165438      9.92468086566785
H   -2.33313759102378     16.47185650315107     10.61677404255658
H   -4.03732100796463     16.38484606998341     11.09737291500822
C   -2.61602492469793     15.74169926269265     13.26097882011913
H   -1.73576522019348     16.39287930127627     13.16890830233927
H   -2.53639064283664     15.14051647760072     14.17857018282661
H   -3.49806392119070     16.39580204669430     13.37632830548420
C   -1.56078647468031     12.41425623408248     10.62216261874106
C   -0.24155390313736     11.74315582642480     10.24138115159847
H   -0.45874390281726     10.89803894647743      9.56430319798551
H    0.29100622355670     11.34016534311033     11.11553951663720
H    0.43765998778867     12.42880571718235      9.72079896254698
C   -2.43417482475834     11.33460172375795     11.26700081924671
H   -2.54543232882918     10.49692906036393     10.55589347144043
H   -3.44073252808307     11.67655508030990     11.52986574307814
H   -1.95996990043464     10.92778361380431     12.17224501217724
C   -2.20298974775271     12.93464160638970      9.33516074544002
H   -1.62206253553685     13.76830411839013      8.90934528272453
H   -3.23766702575026     13.27318595880669      9.48430199464431
H   -2.22557116754545     12.12568913534641      8.58465229709763
C    0.97843648244251     16.07675011659010     14.46110432796982
H    0.74872506214218     16.05117674865189     15.53520756039364
C    1.84293721584782     17.14327785556195     13.89862240653370
H    2.44612479432801     17.64236255399851     14.67394057448539
H    1.19705649562912     17.89552692145066     13.40193789494299
C    3.54595545909073     17.93117094646767     11.61207454058978
C    2.50840323111365     18.45144129614127     10.62042540281885
H    2.92286915780724     19.33641939964136     10.10601977303524
H    1.57257280639773     18.75378937806662     11.10657968708910
H    2.27156135784529     17.69572520968885      9.86216524587105
C    3.87961200130813     19.07475847020924     12.57268284479768
H    2.97544459275737     19.44862552220044     13.07449042522261
H    4.29954703637480     19.91655417104465     11.99358751114878
H    4.61257802443275     18.80493784161083     13.34189229836048
C    4.77808336190394     17.53822760530982     10.79537781371580
H    4.56945324635941     16.66861314348361     10.15349041634706
H    5.65384921513002     17.30768964173152     11.41594810438862
H    5.05298534181562     18.38060309962536     10.13727300862076
```

C	4.26385526594528	15.50562877659078	13.36234184544565
C	3.67874154228762	14.56796214225097	14.42528280977737
H	2.97925893872742	13.82674553113202	14.01423824841491
H	3.17400112975259	15.12397094105629	15.22917758436107
H	4.51246102076495	14.00842533453385	14.88376567475757
C	5.06467495485648	14.66282265363620	12.36067381704907
H	4.42719127415987	14.02535204919751	11.72925049422195
H	5.74478144485321	13.99687009845310	12.91948137053163
H	5.68520993231077	15.27920285022898	11.69813222212198
C	5.20818931250380	16.46180157984656	14.09462967796172
H	5.78540545518935	17.10420531498743	13.41652003407502
H	5.93601588198208	15.86393113101365	14.67034335942109
H	4.67513748687834	17.10345630119651	14.81350217560842
C	1.74093678097346	14.49336891795802	8.68832234886169
H	2.33469256069845	13.72136204982390	9.20043190115344
C	1.71659096307781	14.61507143270284	7.30873821757260
H	2.30085226751970	13.91376497917911	6.70935853434650
C	0.94566644425543	15.62089623630303	6.71374919139672
H	0.89694605244121	15.73022311132974	5.62736538423009
C	0.25549938779014	16.48700592945267	7.54745636393154
H	-0.34304913768012	17.30744774475022	7.14682300310489
C	0.32853048432668	16.33067250450745	8.93682365926806
C	-0.27207805328766	17.24022196157265	9.87511659272968
C	-0.93648546933951	18.47408524370027	9.77108878697745
H	-1.23455545023294	19.00614985289526	8.87051301410911
C	-1.10490256484275	18.85681925875303	11.10335391802932
H	-1.57502217942361	19.75698913058274	11.50178307361244

3^{K}

130
E(SCF) = -4086.28046654; E0 = 1.12040678

Re	0.90898212285784	15.16718130276352	11.67044824385954
K	2.82886571894346	10.01338788710171	11.66425174275758
P	-1.20934018920613	13.99939672411612	11.82886139057061
P	2.82970506773730	16.43123098419631	12.48280636705180
P	1.93989420261738	13.18281558296419	11.81797097317780
O	2.94661467085107	12.62915763371189	10.78524215502237
O	1.68518515729388	12.10763389987580	12.90049084626814
O	5.30046725012306	10.41176150348367	10.16255067602863
O	3.01405618333656	9.23171642316507	8.88052319768582
O	1.42806951206740	7.65499637266143	10.56112084196540
O	1.07539553576210	8.39213148952423	13.23678060839995
O	3.06397372721422	9.78069054813782	14.67159421209924
O	5.00545930571461	10.97149395049141	12.95967980233968
N	0.50537698915985	15.33633761645243	13.71039566185272
N	1.04384609775580	15.33856442808637	9.53131063717789
N	-0.04386591256185	17.03673077083485	11.19530237870579
N	-0.52477306141842	18.02534105769806	11.93769058991614
C	-0.24881648958111	14.30951089383821	14.44448308569597
H	-0.90608779816994	14.80499579567470	15.17705118621481
H	0.49756488634109	13.72615913434935	15.00828644853162
C	-1.03129151966955	13.34917196657289	13.55459661297997
H	-2.00243463700286	13.09277065610875	14.00512812605105
H	-0.43702846549358	12.43204660136502	13.44010057169529
C	-2.86554559971160	14.96919025158598	11.96001083214977
C	-4.10513500530730	14.11892277619439	12.24830154991484
H	-4.39450921599050	13.46777973286335	11.41281739052413
H	-4.95255409949310	14.80192414981998	12.43136324413919
H	-3.99336502891522	13.50132366634649	13.15294668083676
C	-3.11633691305889	15.79543496247349	10.69568409459342
H	-3.37550135152299	15.17391827022101	9.82926372225748
H	-2.25080283573334	16.40998001149778	10.42699505149399
H	-3.96478744867994	16.47696463237481	10.87479416542275
C	-2.69657612885724	15.92249493261953	13.14907755117440
H	-1.77109213776937	16.51113833831110	13.09615637443792
H	-2.73978205870031	15.37913695108421	14.10490238565652
H	-3.53378631926825	16.64006041249331	13.14734132679815
C	-1.60019364029909	12.41049744280819	10.80964965086162
C	-0.30222942767040	11.73433441033720	10.37289402440995
H	-0.55157147555406	10.85556599879988	9.75151842485527
H	0.27504701591227	11.38653764578433	11.23838259351434
H	0.33114606918512	12.39939009121913	9.77286319053025
C	-2.38655407207412	11.37204856621774	11.61651024547954

H	-2.64113475758591	10.52391498612106	10.95646745055898
H	-3.32121373106099	11.75270110742610	12.04356854686170
H	-1.77725317323445	10.97124904001951	12.44037650361925
C	-2.33746849656683	12.80003136953419	9.52605592526151
H	-1.79635217760291	13.59365273946646	8.98730081433394
H	-3.36638836072217	13.14051783615027	9.69782061141645
H	-2.38849016129203	11.92103868143910	8.86130775666578
C	0.91903149359147	16.34627955689311	14.40645532133204
H	0.59729374338269	16.45759229139961	15.45196694004209
C	1.86191826431102	17.32497185296096	13.81261991221150
H	2.46101731651195	17.83632442049895	14.58211173076685
H	1.26981104884039	18.08091590707433	13.26003555685292
C	3.69413750721928	17.86257677291692	11.54967247958231
C	2.71876136891250	18.34756626060856	10.47802132870794
H	3.19543579798570	19.16460565019365	9.91073851370578
H	1.78242560049385	18.73561958038862	10.89947107648467
H	2.47070766499469	17.54263903665863	9.77622786685764
C	4.05801488257987	19.05956737908969	12.43198308410305
H	3.16486254081947	19.52003729917325	12.87805972927952
H	4.53820383784229	19.82802720412464	11.80251903094271
H	4.76006032540033	18.81660947127232	13.23802570562141
C	4.93738191226396	17.34204704189013	10.82773680444379
H	4.71161578378421	16.44143298811125	10.23633140880701
H	5.76388971931614	17.11403120026026	11.51307687348181
H	5.29572310443269	18.11655227076199	10.12981638943871
C	4.18502200375643	15.52990977278872	13.51045581663715
C	3.49348494463273	14.66832482691887	14.57163761754222
H	2.76806905130284	13.96919546755304	14.13411113080815
H	2.97754376056911	15.27685157547505	15.32771645224597
H	4.26632291615525	14.08084026599176	15.09699322949602
C	5.01079504980160	14.60046743704452	12.61471730010420
H	4.37849524867243	13.93866992042716	12.00717116472181
H	5.64768804202123	13.96378021378003	13.25293459305553
H	5.67463841125350	15.15038611638782	11.93819971311296
C	5.12390511540698	16.48002991508351	14.25535693849467
H	5.76535830250287	17.06733840349831	13.58581600720319
H	5.79116354839974	15.88064704190690	14.89866281486279
H	4.57732805127128	17.17231903126394	14.91335185616735
C	1.71471824907743	14.49279244892632	8.71806381499671
H	2.26887875457764	13.70046481874511	9.23338394511861
C	1.72489184814450	14.61626038133007	7.33899003229438
H	2.29188036948077	13.89589467793697	6.74648294303299
C	1.01297901122012	15.66003220412043	6.73742266040726
H	0.99889704632904	15.78549964025909	5.65235951314107
C	0.33866890717824	16.54825038449297	7.55900122950401
H	-0.21832572213965	17.39128460916026	7.14720060216736
C	0.38122772749138	16.38645905913132	8.94945700541761
C	-0.19613182732212	17.32022839581901	9.87724530991412
C	-0.82453103218666	18.56988270450805	9.76396698774480
H	-1.10335487102954	19.10759182042062	8.86109459911304
C	-0.99986349994830	18.96009626557957	11.09350883607941
H	-1.45263018998820	19.87037635727454	11.48779611934478
C	6.21579688106410	11.06885587188766	12.26571305109978
H	6.76675147103743	10.10694629192401	12.29308111021965
H	6.86533870383822	11.83876287590728	12.72556199981796
C	5.92636975778990	11.47222079692968	10.84407129174202
H	5.25612228056665	12.34827071334351	10.85322252902154
H	6.87568304434866	11.74898447887250	10.34095556109965
C	4.79308176966603	10.81456939348420	8.91461674254276
H	5.60591919360095	11.21719128034034	8.27649501690587
H	4.03270034535852	11.60525244522253	9.05386568351751
C	4.19262366874527	9.62244089917730	8.22493903778409
H	3.97650701305547	9.88301675121457	7.16950718371147
H	4.92787327147332	8.79412499374431	8.23328244528993
C	2.54981052161104	7.96898330804556	8.50449735698419
H	3.27071434454535	7.18051598566457	8.80032460165897
H	2.41147090777468	7.89679956247133	7.40695682610659
C	1.23015010554141	7.71899872553097	9.17663974864871
H	0.52525427339419	8.53444552785288	8.92027726039396
H	0.79675045175439	6.77142569095828	8.79781268775998
C	0.24455770119481	7.39139065844287	11.26695172775513
H	-0.24474747655212	6.47521138580761	10.87925400786965
H	-0.47206753624605	8.22818699111555	11.14847065627712

C	0.55512814142197	7.20220476896732	12.72625237924470
H	-0.38143374026056	6.91885206470975	13.24784969997445
H	1.27187769099866	6.36725454096118	12.85828961491527
C	1.12350787960619	8.45642575304244	14.63676272426303
H	1.74942765063801	7.63864848419813	15.04429683647989
H	0.10577350088338	8.34595283672491	15.06148039232812
C	1.70845457653792	9.78511508888181	15.02988552670585
H	1.17865950395075	10.59947092303457	14.50312308386776
H	1.58384655818544	9.93400438583616	16.12270598818153
C	3.71728488947138	10.99583579073355	14.93703996076219
H	3.78317560094243	11.17389370794285	16.03034027655141
H	3.16045698818132	11.82450946101956	14.46772447618093
C	5.10319272200272	10.93449860518837	14.35551331036408
H	5.68974893071475	11.79972768513203	14.72299731450684
H	5.61586046075013	10.01186610919308	14.69520944024939

References

[1] a) D. Savoia, C. Trombini, A. Umani-Ronchi, *J. Org. Chem.* **1978**, *43*, 2907; b) J. Abbenseth, M. Diefenbach, A. Hinz, C. Würtele, J. M. Goicoechea, M. C. Holthausen, S. Schneider, *Angew. Chem. Int. Ed.* **2019**, *58*, 10966.

[2] I. Noviandri, K. N. Brown, D. S. Fleming, P. T. Gulyas, P A. Lay, A. F. Masters, L. Phillips, *J. Phys. Chem. B* **1999**, *103*, 6713.

[3] Tetrahydrofuran - High Purity Solvents, https://www.sigmaaldrich.com/chemistry/solv ents/tetrahydrofuran-center.html (visited on Mar. 20, 2020).

[4] W.M. Haynes, *CRC Handbook of Chemistry and Physics* **2013-2014***, 94th Edition*. CRC Press LLC, Boca Raton, FL, 6-238.

[5] b) APEX2 v2014.9-0 (SAINT/SADABS/SHELXT/SHELXL), Bruker AXS Inc., Madison, WI, USA, **2014**. c) G. M. Sheldrick, *Acta Cryst.* **2015**, A71, 3. d) G. M. Sheldrick, *Acta Cryst*. **2015**, C71, 3. e) G. M. Sheldrick, *Acta Cryst*. **2008**, A64, 112-122.

[6] A. L. Spek *Acta Cryst.* **2015**, *C7***1**, 9.

[7] (a) F. Neese, *Wiley Interdiscip. Rev. Comput. Mol. Sci.* **2012**, *2*, 73. (b) F. Neese, *Wiley Interdiscip. Rev. Comput. Mol. Sci.* **2017**, e1327.

[8] J.P. Perdew, K. Burke, M. Ernzerhof, *Phys. Rev. Lett.* **1996**, *77*, 3865.

[9] (a) B. I. Dunlap, J. W. D. Connolly and J. R. Sabin, *J. Chem. Phys.* **1979**, *71*, 3396-3402. (b) M. Feyereisen, G. Fitzgerald and A. Komornicki, *Chem. Phys. Lett.* **1993**, *208*, 359-363.

[10] (a) K. Eichkorn, F. Weigend, O. Treutler, R. Ahlrichs, *Theor. Chem. Acc.* **1997**, *97*, 119. (b) D. Andrae, U. Haeussermann, M. Dolg, H. Stoll, H. Preuss, *Theor. Chim. Acta* **1990**, *77*, 123. (c) F. Weigend, R. Ahlrichs, *Phys. Chem. Chem. Phys.* **2005**, *7*, 3297. (d) F. Weigend, M. Häser, H. Patzelt, R. Ahlrichs, *Chem. Phys. Lett.* **1998**, *294*, 143.

[11] A. V. Marenich, C. J. Cramer, D. G. Truhlar, J. *Phys. Chem. B* **2009**, *113*, 6378.

[12] S. Grimme, *Chem. Eur. J.* **2012**, *18*, 9955.

[13] C. Adamo, V. Barone, *J. Chem. Phys.* **1999**, 110*,* 6158–6170.

[14] F. Neese, F. Wennmohs, A. Hansen, U. Becker, *Chem. Phys.* **2009**, *356*, 98.

[15] Y. Zhao, D. G. Truhlar, *J. Chem. Phys.* **2006**, *125*, 194101.

[16] Y. Zhao, D. G. Truhlar, *Theor. Chem. Acc.* **2007**, *120*, 215.

[17] NBO 6.0: E. D. Glendening, J. K. Badenhoop, A. E. Reed, J. E. Carpenter, J. A. Bohmann, C. M. Morales, C. R. Landis and F. Weinhold (Theoretical Chemistry Institute, University of Wisconsin, Madison, WI, 2013); http://nbo6.chem.wisc.edu/.

[18] (a) R. Ditchfield, *J. Chem. Phys.* **1972**, 56, 5688. (b) T. Helgaker, M. Jaszúnski, K. Ruud *Chem. Rev.* **1999**, 99*,* 293

[19] F. Jensen, *J. Chem. Theory Comput.* **2015**, *11*, 132.

[20] V. Barone, M. Cossi, *J. Phys. Chem. A* **1998**, *102* (11), 1995.

[21] (a) G. Schreckenbach, T. Ziegler, *J. Phys. Chem.* **1995**, *99*, 606; (b) J. Autschbach, E. Zurek, *J. Phys. Chem. A* **2003**, *107*, 4967 (c) M. Krykunov, T. Ziegler, E. v. Lenthe, *Int. J. Quant. Chem.* **2009**, *109*, 1676.

[22] G. te Velde, F. M. Bickelhaupt, E. J. Baerends, C. Fonseca Guerra, S. J. A. van Gisbergen, J. G. Snijders and T. Ziegler, *J. Comput. Chem.* **2001,** *22*, 931. ADF 2019, SCM, Theoretical Chemistry, Vrije Universiteit, Amsterdam, The Netherlands, http://www.scm.com.

[23] (a) E. van Lenthe, E. J. Baerends, J. G. Snijders, *J. Chem. Phys.* **1993**, *99*, 4597; (b) E. van Lenthe, E. J. Baerends, J. G. Snijders*, J. Chem. Phys.* **1994**, *101*, 9783; (c) E. van Lenthe, J. G. Snijders, E. J. Baerends, *J. Chem. Phys.* **1996**, *105*, 6505; (d) S. K. Wolff, T. Ziegler, E. van Lenthe, E. J. Baerends, *J. Chem. Phys.* **1999**, *110*, 7689.

[24] E. van Lenthe, E. J. Baerends, *J. Comput. Chem.* **2003**, *24*, 1142.

[25] (a) A. Klamt, G. Schüürmann, *J. Chem. Soc., Perkin Trans. 2* **1993**, *5*, 799-805; (b) C. C. Pye, T. Ziegler, *Theor. Chem. Acc.* **1999**, *101*, 396.

[26] CYLview, 1.0b; Legault, C. Y., Université de Sherbrooke, 2009 (http://www.cylview.org).

[27] Jmol: an open-source Java viewer for chemical structures in 3D. http://www.jmol.org/.

[28] Chemcraft - graphical software for visualization of quantum chemistry computations. https://www.chemcraftprog.com
